# A Multiplexing Activity-Based Protein-Profiling Platform for Dissection of a Native Bacterial Xyloglucan-Degrading System

**DOI:** 10.1021/acscentsci.3c00831

**Published:** 2023-11-24

**Authors:** Nicholas G. S. McGregor, Casper de Boer, Quentin P. O. Foucart, Thomas Beenakker, Wendy A. Offen, Jeroen D. C. Codée, Lianne I. Willems, Herman S. Overkleeft, Gideon J. Davies

**Affiliations:** † Department of Chemistry, The University of York, Heslington, York YO10 5DD, United Kingdom; ‡ Leiden Institute of Chemistry, Leiden University, Einsteinweg 55, 2300 RA, Leiden, The Netherlands; § York Structural Biology Laboratory and York Biomedical Research Institute, Department of Chemistry, University of York, Heslington, York YO10 5DD, United Kingdom

## Abstract

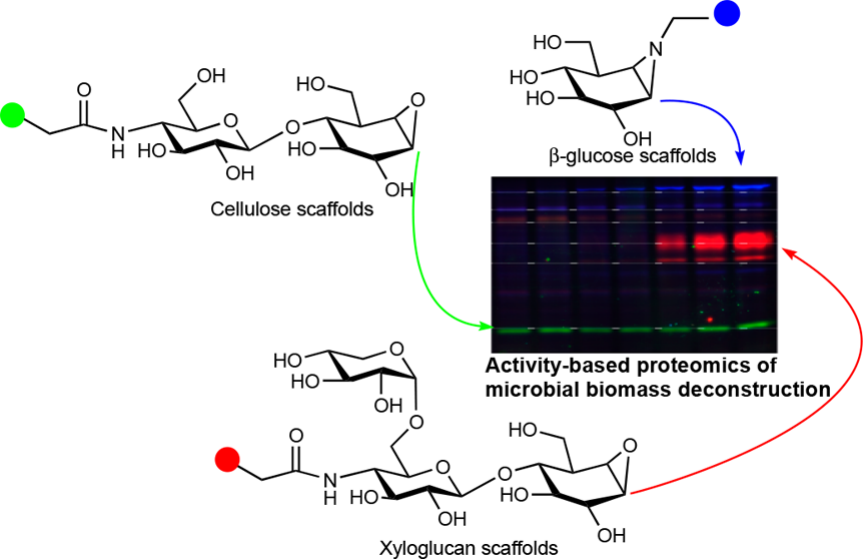

Bacteria and yeasts grow on biomass polysaccharides by expressing and excreting a complex array of glycoside hydrolase (GH) enzymes. Identification and annotation of such GH pools, which are valuable commodities for sustainable energy and chemistries, by conventional means (genomics, proteomics) are complicated, as primary sequence or secondary structure alignment with known active enzymes is not always predictive for new ones. Here we report a “low-tech”, easy-to-use, and sensitive multiplexing activity-based protein-profiling platform to characterize the xyloglucan-degrading GH system excreted by the soil saprophyte, *Cellvibrio japonicus*, when grown on xyloglucan. A suite of activity-based probes bearing orthogonal fluorophores allows for the visualization of accessory *exo*-acting glycosidases, which are then identified using biotin-bearing probes. Substrate specificity of xyloglucanases is directly revealed by imbuing xyloglucan structural elements into bespoke activity-based probes. Our ABPP platform provides a highly useful tool to dissect xyloglucan-degrading systems from various sources and to rapidly select potentially useful ones. The observed specificity of the probes moreover bodes well for the study of other biomass polysaccharide-degrading systems, by modeling probe structures to those of desired substrates.

## Abstract 2

Bespoke activity-based probes are synthesized and applied for the microbial deconstruction of xyloglucan biomass allowing dynamic imaging of complex enzyme mixtures from microbial secretomes.

## Introduction

Natural biodiversity presents a wealth of strategies to enhance fitness by solving complex biological problems. However, when it comes to breaking down biomass, these solutions are not fully understood due to the intricacies of deciphering the roles and behaviors of complex multienzyme systems. Significant effort is currently devoted to obtaining such information as a means to improve functional inference,^1−3^ facilitate biomolecule characterization,^4^ clarify microbial niches,^5,6^ and facilitate the reconstitution of enzyme systems in fermentative workhorse organisms.^7,8^ Comprehensive methods that allow the sensitive and specific detection of several active enzymes simultaneously within a native proteomic background will pave the way to the efficient screening of microbes for biomass-degrading potential. Such methods would, in turn, spotlight vital enzyme components that act on different parts of substrates, aiding in the conversion of biomass polysaccharides into sustainable energy and resources for the chemical sector.

**Figure 1:**
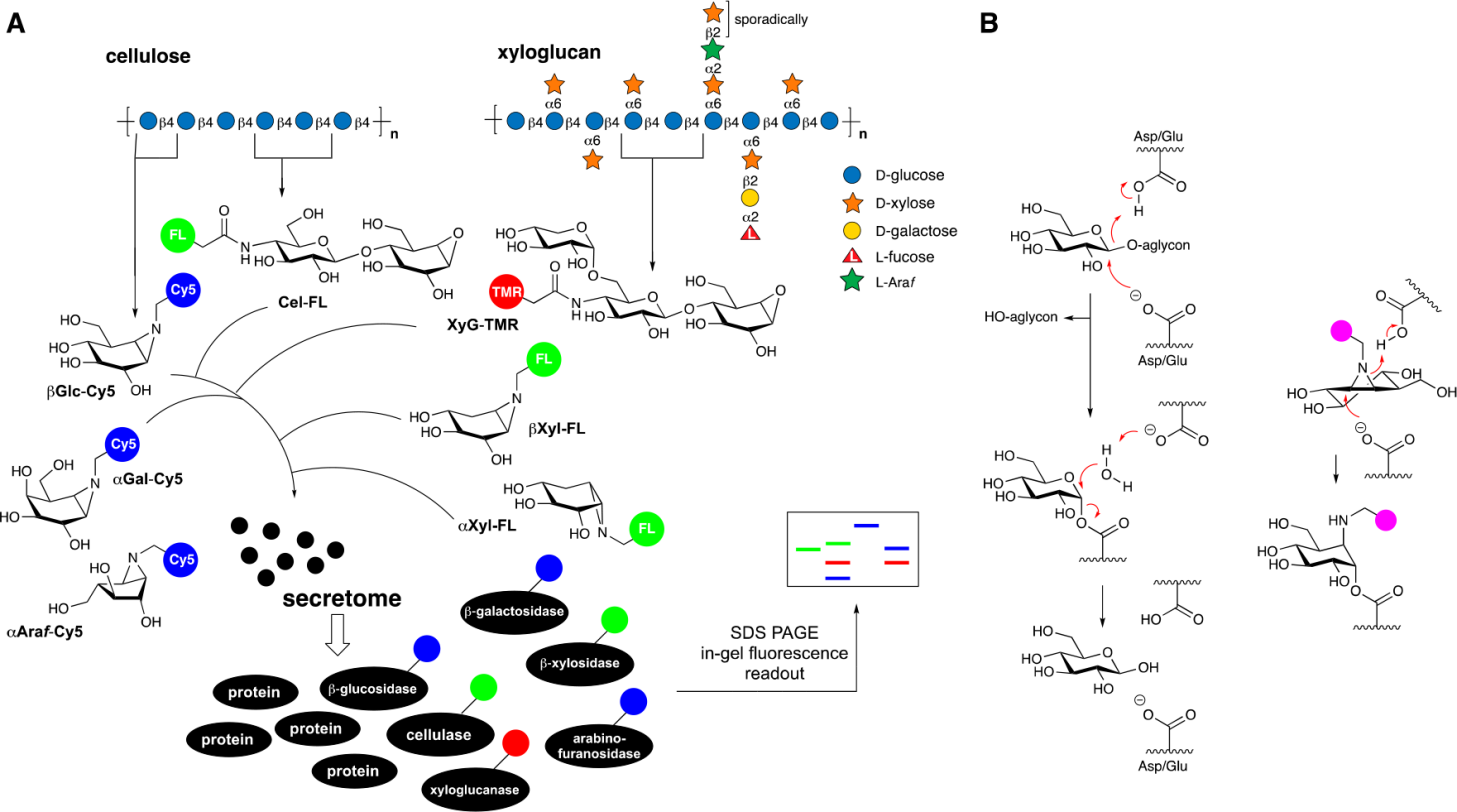
(A) Strategy of the multiplexing activity-based protein profiling (ABPP) platform subject of the here-presented study. Activity-based probes (ABPs) bearing orthogonal fluorophores are designed to emulate xyloglucan/cellulose structural elements. Treatment of secretomes of microbes from various sources grown on xyloglucan followed by SDS-PAGE resolution will yield color-coded fingerprints of both *exo*-acting and *endo*-acting GHs. (B) Mode of action of a retaining β-*exo*-glucosidase and its mechanism-based, covalent, and irreversible inhibition, thereby labeling, by cyclophellitol aziridine ABPs.

One such biomass polysaccharide with major, yet largely unrealized, biotechnological potential is xyloglucan (Figure 1A). Xyloglucan is a ubiquitous cellulose-binding β-(1,4)-glucan with diversified α-(1,6)-xylose branches extending from two to three of each set of four glucose residues in a semiregular species-specific pattern.^9,10^ Xyloglucan branches have been found to contain β-(1,2)-galactopyranose, α-l-(1,2)-fucopyranose, α-l-(1,2)-arabinofuranose, and, in rare circumstances, β-(1,2)-xylopyranose residues,^11^ among others.^9^

Xyloglucan is a predominant hemicellulose in the primary cell walls of many plants.^12^ As a result, it is an important source of dietary fiber that sustains key gut microbiota.^13^ Xyloglucan is important for plant development. Its structure and modifications can dictate the mechanical properties of plant cell walls, playing a crucial role in plant growth and response to environmental stresses. Alterations to the xyloglucan structure in the plant cell wall can render plants less susceptible to pathogenic attack.^14^ Xyloglucan in soil can support the growth of myriad microbial species, sustaining microbial diversity and, by extension, soil health.^15^ The diversity of soil microbes that degrade xyloglucan hints at its importance in soil communities.^16^ Understanding the behaviors of xyloglucan-degrading microbes may yet provide insights into novel biological control strategies for plant diseases.

Effective xyloglucan-degrading GH systems (recently reviewed by Attia and Brumer^17^) contain *exo*-acting glycosidases acting as debranching accessory enzymes and *endo*-acting enzymes (xyloglucanases) that generate short, branched β-(1,4)-glucan oligomers (xyloglucan oligosaccharides).^18−23^*Exo*-acting enzymes are expected to address each of the glycosidic linkages reported in xyloglucan branches, though the reported enzyme diversity falls short of reported branch diversity.^9,24^*Endo*-acting enzymes, taking on both linear and branched β-(1,4)-glucans, are needed to effectively degrade xyloglucan, yet discrimination between their substrate specificities using contemporary methods (primary sequence alignment, structure alignment) is unreliable, rendering comparisons of xyloglucan-degrading systems from different organisms challenging.

*Cellvibrio japonicus* is a saprotrophic bacterium which possesses the remarkable ability to grow in isolation using a variety of hemicellulosic polysaccharides as sole carbon sources.^25−27^ It produces a diverse collection of GHs, assembling enzyme systems that can degrade cellulose, xylans, mannans, and xyloglucans, among others.^28,29^ In contrast to polysaccharide utilization loci found in many gut bacteria,^5^*C. japonicus* polysaccharide-degrading systems are not tightly organized into complete substrate-specific gene clusters, complicating the enumeration of the components of a complete enzyme system. Recent transcriptomic work identified a cluster of four genes within the *C. japonicus* genome, three of which are essential for xyloglucan oligosaccharide (XyGO) saccharification.^28^ However, this gene cluster does not include any apparent xyloglucanase or *exo*-β-glucosidase or any other possible accessory activities. Recombinant production and characterization of homology-selected putative xyloglucanases in the *C. japonicus* genome identified Cel5D, Cel5E, Cel5F, and CjGH74A as specific xyloglucanases, but the roles of each of these remain unclear.^30,31^ Among *exo*-β-glucosidases, Cel3D was found to be xyloglucan oligosaccharide-specific.^27^ Yet, knocking out these genes only generated a mild growth phenotype, suggesting the presence of additional compensating enzymes.

With the aim to annotate these compensating *exo*-glycosidases and to allow for rapid discrimination between cellulose- and xyloglucan-acting *endo*-glycosidases, we developed a multiplexing activity-based protein-profiling (ABPP) assay, the results of which are presented here. ABPP allows for the rapid and sensitive functional annotation of active enzymes in complex biological samples.^32−35^ Key to the predictive value of an activity-based probe (ABP) is its enzyme selectivity, and we have found in the past that fluorescent, configurational, and functional isosteres of the natural retaining β-glucosidase inhibitor, cyclophellitol, are eminently suited to forecast the substrate preference of both *exo*- and *endo*-acting glycosidases.^36−45^ More so than other GH-directed probe designs, which are often limited in activity and/or GH selectivity, cyclophellitol-based ABPs are viable tools to assess polysaccharide-induced microbial secretomes for desirable activities, which can then be selected for further annotation. Besides targeting a single GH within a biological system, ABPP assays can be executed in a multiplexing format,^44,45^ allowing dissection of complex enzyme systems such as that of the xyloglucan degradome of *C. japonicus* studied here.

The work presented here comprises the design and validation of trisaccharidic xyloglucan (“XyG”)-type cyclophellitol probes and their validation as *bona fide*, predictive tools for the identification of xyloglucanase activities and their discrimination from cellulase activities within a xyloglucan-elicited *C. japonicus* degradome. The XyG probes complement our previously described^36−45^ suite of *exo*- and *endo*-GH probes, which we combined to investigate the time-dynamic and substrate concentration-dependent expression of xyloglucanases, cellulases, and retaining β-*exo*-glucosidases in secretomes obtained from *C. japonicus* grown on xyloglucan, xyloglucan oligomers, and other polysaccharide food sources. In this way and using both in-gel detection (with fluorescent probes) and proteomics annotation (with biotinylated probes), we revealed Cel5D and Cel5F to be the exclusive specific retaining xyloglucanases (inverting glycosidases cannot be detected with cyclophellitols), filling distinct functional niches, with Cel5C being a cellulase and Cel3A, Cel3B, and Cel3D acting as β-*exo*-glucosidases. Utilization of α-l-arabinofuranose, β-galactopyranose, β-xylopyranose, and α-xylopyranose configured cyclophellitol probes allowed further in-depth dissection of *C. japonicus* secretomes and lysates. Our results provide a blueprint for designing multiplexing ABPP assays for the rapid profiling of secretomes of microorganisms grown on specific polysaccharide materials and in which the probes are designed to represent structural elements of the carbohydrate source and, therefore, activities of the corresponding retaining GHs.

## Results and Discussion

### Assembly and Validation of the Suite of Activity-Based Probes

Xyloglucan (XyG), the primary carbohydrate source used in this study, contains α-(1,6)-xylose branches at the +2 and/or +3 glucose residues of linear tetraglucose stretches. Other branching sugars include β-(1,2)-galactopyranose, α-l-(1,2)-fucopyranose, α-l-(1,2)-arabinofuranose, and, in rare circumstances, β-(1,2)-xylopyranose.^11^ These structures (with the exception of fucose) are captured in the set of mono-, di-, and trisaccharidic cyclophellitol probes as depicted in Figure 1A. These ABPs react with their target GH in a mechanism-based fashion to form a covalent and irreversible enzyme–inhibitor adduct as depicted for inactivation and tagging of retaining β-*exo*-glucosidases in Figure 1B. Variation in configuration and substitution pattern yields probes designed to target xyloglucanases (denoted as **ABP-XyG**), cellulases (**ABP-Cel**),^45^ retaining β-*exo*-glucosidases (**ABP-βGlc**),^37^ retaining β-*exo*-galactosidases (**ABP-βGal**),^46^ retaining β-*exo*-xylosidases (**ABP-βXyl**),^44^ retaining α-*exo*-xylosidases (**ABP-αXyl**), and retaining α-l-*exo*-arabinofuranosidases (**ABP-αAraf**).^38^ The probes were prepared in fluorescent form bearing either a Cy5 dye (denoted with the extension **Cy5**), a Cy3 dye (**Cy3**), or a Bodipy-FL dye (**FL**) to allow for in-gel multiplexing ABPP detection. For the purpose of kinetic measurements, probes were prepared with simple azide (**N3**) tags. For the purpose of target GH identification by pull-down/mass spectrometry proteomics, all probes were also prepared in biotinylated form (**Bio**). With the exception of the **ABP-XyG** and **ABP-αXyl** probes, the synthesis and labeling efficacy of all probes on recombinant and/or isolated GHs, as well as detection of these in complex biological samples, have been reported previously.^37,38,44−46^ The full structures of all probes and the synthesis of the **ABP-XyG** and **ABP-αXyl** probes are given in the Supporting Information.

**Figure 2:**
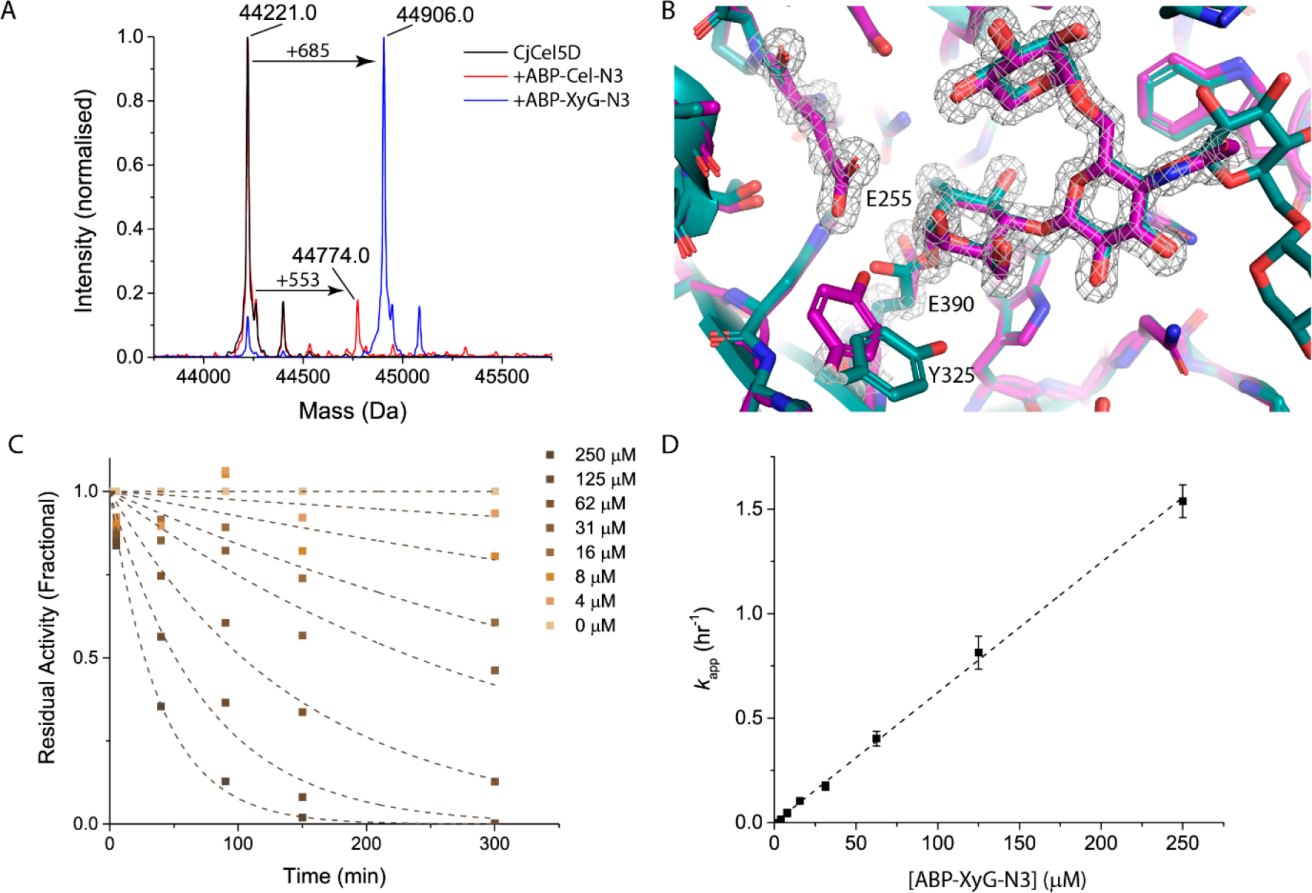
Labeling of xyloglucanases with inhibitors and probes. (A) Intact MS of CjGH5D xyloglucanase treated with 100 μM **ABP-Cel**-**N3**, 100 μM **ABP-XyG**-**N3**, or vehicle control for 1 h. (B) Crystal structure of CjCel5D labeled with **ABP-XyG-N3** (purple). 2F_o_–F_c_ density is shown for the ligand and catalytic residues as a gray mesh contoured at 2σ. The complex between CjCel5D and 2-fluoro-XXXG (PDB 6HAA) is superimposed in teal. (C) Residual activity kinetics of CjCel5D inhibited by different concentrations of **ABP-XyG**-**N3**. The model fits are shown as dashed lines. 6C4MU-XXXG was used as substrate. (D) *k*_app_ vs inhibitor concentration for CjCel5D interacting with **ABP-XyG**-**N3**. The model fit is shown as a dashed line.

In order to validate the **ABP-XyG** probes for profiling xyloglucanases in complex biological samples, we first established the potency and mode of action of **ABP-XyG**-**N3** as inhibitor of various previously characterized recombinant xyloglucanases. Incubation of pure recombinant *Bacteroides ovatus* BoGH5A,^13^*Paenibacillus pabuli* PpXG5,^19^ and *C. japonicus* CjCel5D^30^ with 100 μM **ABP-XyG**-**N3** for 1 h under optimal activity conditions gave near-quantitative labeling as assessed by intact protein mass spectrometry analysis, while identical treatment with **ABP-Cel**-**N3** gave minimal labeling (Figure 2A, Supplemental Figures 2–3). Pretreatment of **ABP-XyG**-**N3** and **ABP-XyG-Cy5** with 0.1 mg/mL BoGH31 α-xylosidase for 1 h at 37 °C caused no significant change in labeling behavior and no detectable formation of a dexylosylated species by LC-MS, indicating that **ABP-XyG**-**Cy5** and **ABP-XyG-N3** are resistant to *exo*-hydrolase activity (Supplemental Figure 4). This is consistent with the known recognition mode of xyloglucan-specific α-xylosidases, requiring an unsubstituted nonreducing chain terminus.^47,48^

X-ray diffraction data of the complex between CjCel5D and **ABP-XyG**-**N3** shows near-perfect mimicry of the known glycosyl enzyme intermediate state in the −1 and −2 subsites (Figure 2B). Irreversible inhibition kinetics, measured using bespoke 4-methylumbelliferyl (4MU) and 6-chloro-4-methylumbelliferyl (6C4MU) XXXG fluorogenic substrates (Figure 2C and D and Supplemental Figures 5–25; see the Supporting Information for synthetic details and Tuomivaara et al. for oligosaccharide nomenclature),^9^ showed probe selectivity values ((*k*_i,XyG-N3_/*K*_I,XyG-N3_)/(*k*_i,Cel-N3_/*K*_I,Cel-N3_)) ranging from >17 for CjCel5D to 0.13 and <0.0026 for HiCel7B^49^ and BaCel5A,^50^ two well-known cellulases, respectively (Table 1).

**Table 1:** Kinetic Parameters for Covalent Inhibition of *endo*-Glucanases by **ABP-Cel-N3** and **ABP-XyG**-**N3**^a^

enzyme	compound	*K*_I_ (μM)	*k*_inact_ (min^–1^)	*k*_inact_/*K*_I_ (M^–1^ s^–1^)	specificity
PpXG5	**ABP-Cel-N3**	ND	ND	<0.1	>7.8
	**ABP-XyG-N3**	>500	>3	0.78 ± 0.08	
BoGH5	**ABP-Cel-N3**	ND	ND	<0.1	ND
	**ABP-XyG-N3**	ND	ND	<0.1	
CjCel5D	**ABP-Cel-N3**	ND	ND	<0.1	>17
	**ABP-XyG-N3**	>500	>1.4	1.7 ± 0.1	
BaCel5A	**ABP-Cel-N3**	>200	>0.5	39 ± 3	<0.0026
	**ABP-XyG-N3**	ND	ND	<0.1	
HiCel7B	**ABP-Cel-N3**	3.9 ± 0.3	0.50 ± 0.04	2100	0.13
	**ABP-XyG-N3**	22 ± 2	0.36 ± 0.03	270	
CjCel5B	**ABP-Cel-N3**	>250	>1	41 ± 3	0.14
	**ABP-XyG-N3**	>250	>0.2	5.6 ± 0.8	
CjCel5C	**ABP-Cel-N3**	11 ± 1	0.116 ± 0.005	97	<0.0010
	**ABP-XyG-N3**	ND	ND	<0.1	

a Where it was not possible to obtain distinct *k*_inact_ and *K*_I_ parameters at the inhibitor concentrations tested, the combined *k*_inact_/*K*_I_ parameter is shown for these cases. ND: not determined. Specificity as determined from the ((*k*_inact,XyG-N3_/*K*_I,ABP-XyG-N3_)/(*k*_inact,Cel-N3_/*K*_I,Cel-N3_) values.

### Dissection of the *C. japonicus* Xyloglucanase Degradomes by Multiplexing ABPP

**Figure 3:**
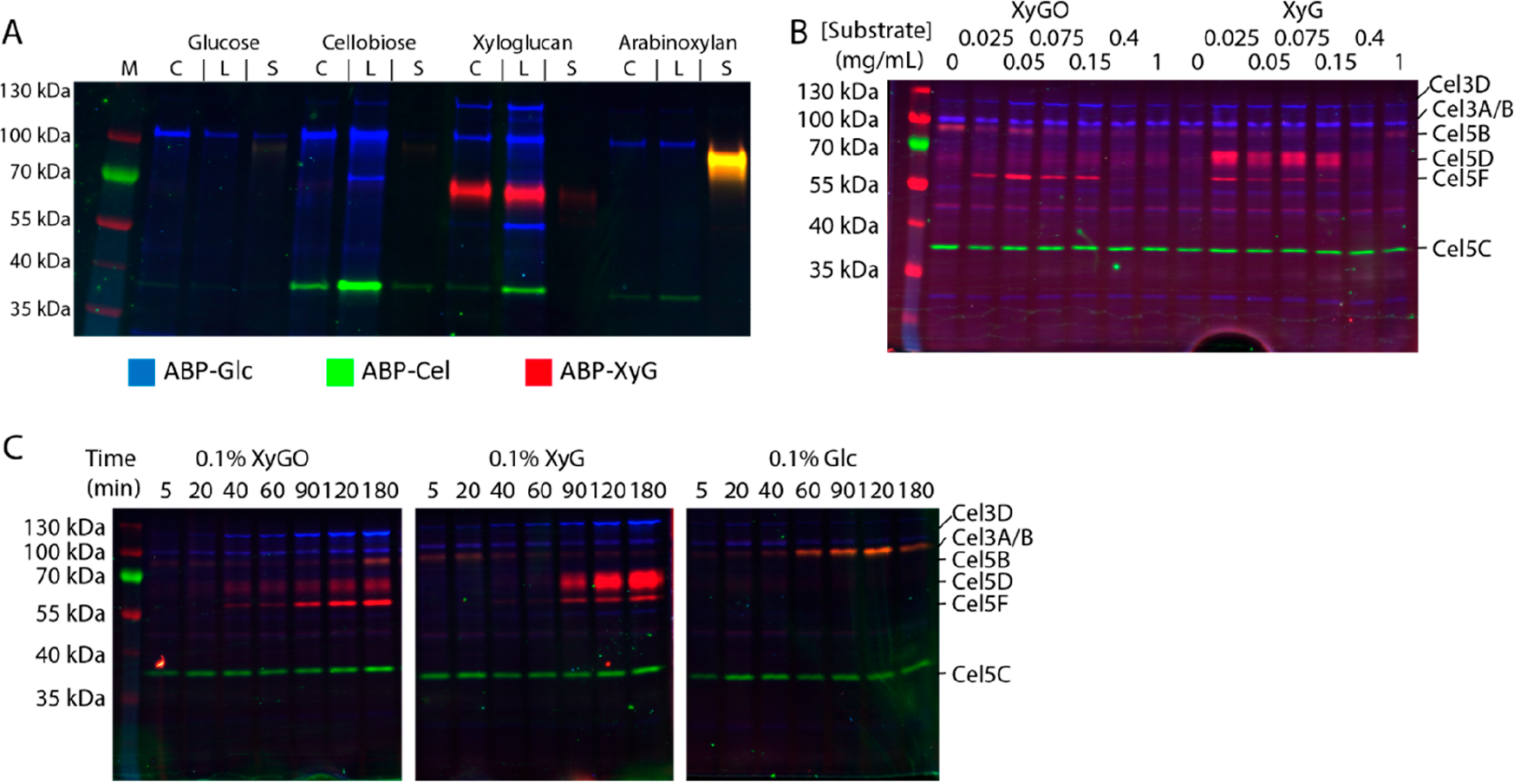
ABPP analysis of *C. japonicus* cultures. (A) Laser-scanning fluorescence image of a 4–20% SDS-PAGE separation of *C. japonicus* proteins following treatment of intact cells (C), lysate (L), or supernatant (S) with a mixture of **ABP-βGlc**-**FL**, **ABP-Cel**-**Cy3**, and **ABP-XyG**-**Cy5**. The carbon source on which the cells were grown is noted above each set of three lanes. (B) Representative gel image from ABPP analysis of *C. japonicus* lysate following 2 h of growth in the presence of increasing concentrations of xyloglucan or xyloglucan oligosaccharides. (C) Representative gel image from ABPP analysis of *C. japonicus* lysate collected over time from growth in the presence of 0.1% xyloglucan oligosaccharides, xyloglucan, or glucose (Glc).

To dissect the native xyloglucan-degrading system of *C. japonicus*, we prepared “primed” cells by growth on glucose to carbon-limited saturation in MOPS minimal medium (see Supporting Information for details). Subsequent dilution into medium containing glucose, cellobiose, tamarind xyloglucan, or wheat arabinoxylan was hypothesized to reveal substrate-specific responses. Secretome, intact cell, and lysate samples from each culture were treated with a triplex probe mixture containing **ABP-βGlc**-**FL**, **ABP-Cel**-**Cy3**, and **ABP-XyG**-**Cy5** (Figure 3A, and Supplemental Figure 26 for Coomassie stain). We observed strong and uniquely xyloglucan-induced production of a ∼65 kDa **ABP-XyG**-**Cy5**-selective outer membrane-associated enzyme. **ABP-βGlc**-**FL** treatment revealed two xyloglucan upregulated glucosidase bands at ∼120 and ∼58 kDa, and **ABP-Cel**-**Cy3** treatment shows a cellulase band at ∼40 kDa also observed in the cellobiose culture. Surprisingly, a major ∼75 kDa secreted band from growth on arabinoxylan reacted with both **ABP-Cel-Cy3** and **ABP-XyG**-**Cy5** (major yellow band). This band was observed at a much lower intensity in samples from growth on glucose and was not observed in samples from growth on xyloglucan.

Pulldowns from saturation cultures using **ABP-βGlc**-**Bio**, **ABP-Cel**-**Bio**, and **ABP-XyG**-**Bio** unambiguously identified Cel5D as the only exclusively **ABP-XyG**-**Bio**-reactive band, Cel5C as the exclusively **ABP-Cel**-**Bio**-reactive band, and Cel3A, Cel3B, and Cel3D as the **ABP-βGlc**-reactive bands (Supplemental File 1). The secreted **ABP-Cel**- and **ABP-XyG**-reactive band in the arabinoxylan secretome (the intense band in Figure 3A, last lane) was identified as Cel5B. Cel5B and Cel5D both ran ∼15 kDa heavier on SDS-PAGE than would be expected from their amino acid sequences. To investigate the origin of this discrepancy, ∼50 μg of native Cel5B was partially purified from 200 mL of secretome collected from growth of *C. japonicus* on arabinoxylan to carbon-limited saturation via ultrafiltration and anion-exchange chromatography (Supplemental Figure 27). SDS-PAGE of the purified protein followed by staining with the Pro-Q Emerald glycoprotein gel stain kit (Invitrogen) yielded a strong glycoprotein signal at the band position of Cel5B (Supplemental Figure 28). Extending this analysis to xyloglucan-grown cell lysate yielded a complex pattern of apparent glycoproteins, including a band at the position of Cel5D. Intact mass of the purified Cel5B measured via denaturing LC-ESI-MS gave a protein peak with a series of deconvoluted mass values from 71 to 75 kDa with spacing of 162 Da, indicating heavy glycosylation with variable hexose content (Supplemantal Figure 29). Acid hydrolysis of the Cel5B sample followed by HPAEC-PAD analysis of the resulting monosaccharides revealed a complex mixture, including peaks that match d-mannose, d-glucose, d-galactose, and l-arabinose standards (Supplemental Figure 30). l-Arabinose and d-xylose may be derived from the arabinoxylan substrate, but glucose, galactose, and mannose must have been synthesized by *C. japonicus*, underscoring the versatility of this species to grow—and derive the building blocks it needs—from such well-defined, single food stocks as used here. Considering the intact MS and monosaccharide composition, we propose that the underlying glycan structure is a galactoglucomannan O-glycan. Peptide LC-MS/MS analysis of native Cel5B digested with ProAlanase (Promega) yielded no detectable peptides from the serine-rich linker between the N-terminal catalytic domain and the C-terminal domain (Supplemental Figure 31).

Having identified the core components of the *C. japonicus* xyloglucan-degrading system, we investigated the time-dynamics and substrate concentration-dependence of xyloglucanase expression. We diluted primed *C. japonicus* cells 10-fold into medium containing either xyloglucan or xyloglucan oligosaccharides. ABPP using **ABP-XyG**-**Cy5** on cells harvested during the early induction with xyloglucan revealed two bands, the lower, sharper band running at the expected molecular weight of Cel5E or Cel5F and a higher, more diffuse Cel5D band (Figure 3B, Supplemental Figure 32). Notably, the lower band was primarily induced by xyloglucan oligosaccharides while Cel5D was primarily induced during growth on xyloglucan. To identify the putative xyloglucanase, primed cells were collected by centrifugation and resuspended in 100 mL of fresh medium containing 150 μg/mL of xyloglucan oligosaccharides. After 2 h of incubation, cells and secretome were separated by centrifugation and tested for **ABP-XyG**-**Cy5**-reactive bands. The band of interest was found exclusively in the secretome while Cel5D was found in the cell fraction, so the secretome was collected and concentrated 50-fold by ultrafiltration prior to pulldown using **ABP-XyG**-**Bio**. This identified Cel5D, Cel5E, Cel5F, and Cel5B (Supplemental Figure 33, Supplemental File 1) as probe-reactive components.

To our surprise, both xyloglucan and xyloglucan oligosaccharides induced xyloglucanase expression more efficiently at low (0.05–0.15 mg/mL) concentrations (Figure 3B, Supplemental Figure 32). This may suggest that *C. japonicus* can adapt to grow on persistently low levels of xyloglucan, such as those reported in soil samples near root tips.^51^ Sampling cultures grown in 0.1% xyloglucan or xyloglucan oligosaccharides over 3 h showed that induction by xyloglucan oligosaccharides occurs rapidly, with a xyloglucanase band detectable after only 30 min (Figure 3C, Supplemental Figure 32). We also observed that growth on glucose resulted in low-level expression of Cel5B, while growth on xyloglucan resulted in expression of Cel5D correlating with a decrease in observed Cel5B activity, suggesting that Cel5B is acting as a “sensing” enzyme that is repressed by the detection of xyloglucan. Cel3A/B and Cel5C showed no change in expression under any condition tested, indicating that these are constitutively expressed. Interestingly, Cel5D was more strongly expressed in the presence of xyloglucan than xyloglucan oligosaccharides, but expression of Cel5D in the presence of xyloglucan occurred with a lag. This may be explained by a period of time required to generate small, inducing fragments from large xyloglucan molecules. Cel5F/Cel5E and Cel3D expression appeared to be driven primarily by xyloglucan oligosaccharides, indicating that they are differentially regulated from Cel5D. Thus, in spite of being secreted, Cel5F/Cel5E do not appear to be “sensing” enzymes since their expression is dependent on induction by xyloglucan fragments. We speculate that it is instead acting as a “booster” enzyme, aiding the solubilization of xyloglucan.

Not having been previously functionally or structurally characterized in detail, we produced and purified Cel5B and Cel5C recombinantly in *E. coli* to assess further the correlation between probe reactivity and enzyme specificity. We found that Cel5B and Cel5C were both cellulases, efficiently degrading carboxymethylcellulose and mixed-linkage β-glucan (Supplemental Table 3). Cel5B showed only weak activity toward tamarind xyloglucan while Cel5C had weak activity toward carob galactomannan and no detectable xyloglucanase activity. Measurements of irreversible inhibition kinetics showed strong selectivity of Cel5C for **ABP-Cel-N3** over **ABP-XyG**-**N3** and only weak selectivity of Cel5B for **ABP-Cel**-**N3** over **ABP-XyG**-**N3**, matching in-gel fluorescence results (Table 1).

To determine the molecular basis for the reactivity of **ABP-XyG**-**Cy5** with CjCel5B but not CjCel5C, we crystallized both enzymes and solved their structures by molecular replacement in both unliganded and ABP-bound forms (Supplemental Figure 34). **Cel** inhibitor bound to CjCel5C displaying torsion angles (Φ,Ψ) of (−83°, 94°) between the nonreducing β-d-glucose and cyclophellitol moiety in **ABP-Cel** (Supplemental Figure 34F). O6′ is recognized in the −2 position by both H87 and Y137, and O2′ is recognized by the backbone carbonyl of S311. In contrast, CjCel5B recognizes **ABP-XyG**-**N3** with (−83°, 133°) torsion angles (Supplemental Figure 34C). The consequent twist in the glucose backbone positions the α-(1,6)-xylose residue above W28 and W33, forming a hydrogen bond between O4 and D64. Notably, the active site cleft of CjCel5B is significantly more open beyond the −2 subsite, so we hypothesized that cellulase-specificity in Cel5B is dictated primarily by an inability to accommodate α-(1,6)-xylose residues in the positive subsites. To test this, we synthesized 4MU-XXXG and 6C4MU-XXXG as fluorogenic xyloglucanase substrates (see the Supporting Information for synthetic details). Kinetics for the hydrolysis of 4MU-XXXG and commercially available 4MU-cellotetraose were measured to isolate contributions to specificity from the negative subsites. Cel5B showed a ∼14-fold preference for 4MU-GGGG over 4MU-XXXG (Supplemental Table 2), roughly in line with its sevenfold preference for **ABP-Cel**-**N3** over **ABP-XyG**-**N3** (Table 1) but highly divergent from its 3000-fold specificity toward carboxymethyl cellulose (CMC) over xyloglucan (Supplemental Table 3), supporting our hypothesis.

The detection of the putative β–xylosidase Xyl39A using **ABP-βGlc**-**FL** (the band around 55 kDa in Figure 3A) was particularly interesting, since this enzyme, having 45% identity to the *Xanthomonas citri* XynB,^52^ is adjacent to Cel5D in the genome. Staining *C. japonicus* lysates with the beta-xylose configured probe, **ABP-βXyl**-**FL** and **ABP-βGlc**-**Cy5**, confirmed the xyloglucan-dependent expression of Xyl39A and also revealed it has specificity toward **ABP-βXyl-FL** (Supplemental Figure 36). Chemical proteomics confirmed that Xyl39A was found in xyloglucan-grown cells and could be pulled down with **ABP-βXyl**-**Bio** and **ABP-βGlc**-**Bio** (Supporting Information, Supplemental File 1) To investigate the specificity of CjXyl39A further, we produced the enzyme recombinantly in *E. coli*. Activity measurements against a variety of 4-methylumbelliferyl (4MU) glycosides showed specific recognition of β-d-xylose over other glycosides (Supplemental Table 4). Functionalization of α-(1,6)-xylose branches with β-(1,2)-xylose has been reported in xyloglucan extracted from the leaves^11^ (but not fruit^53^) of argan trees, and a recent report has identified xyloglucan β-xylopyranosyltransferase from *Vaccinium corymbosum*.^54^ We speculate that the coexpression of Xyl39A and Cel5D during growth on xyloglucan indicates an evolved adaptation of *C. japonicus* toward degradation of β-xylosylated xyloglucan; however, we were not able to obtain a suitable sample of β-xylosylated xyloglucan for testing.

Having dissected the *endo*-β-glucanase, *exo*-β-glucosidase, and *exo*-β-xylosidase components of the native *C. japonicus* xyloglucan-degrading system, we turned to the essential *exo*-α-xylosidase, *exo*-α-l-arabinofuranosidase, and *exo*-β-galactosidase activities as potential handles for characterizing xyloglucan-degrading systems using ABPP. Staining with **ABP-αAraf**-**Cy5** showed the presence of Abf51A in *C. japonicus* under all growth conditions. Abf51A staining was less intense in samples grown on glucose and more intense in samples grown on arabinoxylan (Supplemental Figure 35). We conclude from this that Abf51A displays similar regulatory logic to the *E. coliaraBAD* operon^55^ and is not coregulated with xyloglucan-degrading machinery.

Using **ABP-αXyl**-**Cy5** to detect CjXyl31A in the xyloglucan-grown *C. japonicus* lysate during induction by xyloglucan oligosaccharides revealed the emergence of a band at the expected ∼115 kDa, but identification of the band was hindered by weak reactivity and significant nonspecific labeling (Supplemental Figure 37). We attribute the poor potency and selectivity of this probe to a lack of binding in the positive subsites known to be important for substrate recognition in this enzyme class.^48^ Indeed, the **ABP-αXyl**-**Cy5** probe performs well on purified recombinant CjXyl31A but is less effective in doped lysates, consistent with its poor performance on *C. japonicus* lysates (Supplemental Figure 38). Finally, comparing the reactivity of **ABP-βGal**-**Cy5** and **ABP-βGlc**-**Cy5** in *C. japonicus* cell lysates showed strong, and clearly orthogonal, labeling of putative β-glucosidases and β-galactosidases (Supplemental Figure 39). A pulldown from the xyloglucan-grown lysate using **ABP-βGal**-**Bio** revealed the presence of Bgl35A, the known xyloglucan oligosaccharide-specific β-galactosidase, and Bgl2A, an uncharacterized putative β-galactosidase which was previously reported not to be upregulated in response to growth on xyloglucan (Supporting Information, Supplemental File 1).

## Conclusions

We have developed a platform with which native bacterial xyloglucan-degrading systems can be sensitively detected and functionally interrogated. Dissection of native proteomes derived from *C. japonicus* grown on various polysaccharide food sources using these tools reveals features not previously observed, including the production of β-xylosidase during growth on xyloglucan, low-level secretion of Cel5B which we conclude to be a cellulase during growth on glucose, the occurrence of significant enzyme glycosylation, and the different expression and secretion behaviors of the vanguard xyloglucanases, Cel5D, Cel5E, and Cel5F.

The ability to detect xyloglucanases with such high sensitivity and throughput enabled us to measure the concentration-dependence and time-dependence of xyloglucanase activity traced back to specific enzymes in response to different inducers. This revealed surprisingly sensitive xyloglucan detection by *C. japonicus*. This sensitivity may reflect a low-xyloglucan ecological niche where *C. japonicus* thrives.

Building on these developing capabilities, we envision the assembly of different polysaccharide-specific toolkits to enable the characterization of native component enzymes from diverse microbial polysaccharide-degrading systems. These toolkits will provide a significant boost in speed, data richness, and robustness compared to state-of-the-art carbohydrate zymography techniques.^56^ One significant advantage of cyclophellitol-based probe designs is their specificity, leading to clear, interpretable results as demonstrated here. Indeed, a developing toolkit for the analysis of xylanases and cellulases was recently applied to enzyme discovery from diverse fungal secretomes.^57^ ABPP methods will continue to facilitate the characterization of native component enzymes from a plethora of microbial species, shedding light on the nuanced strategies that microbes employ to degrade and assimilate complex polysaccharides.

Importantly, known, or putative, glycosidase products, which are the result of enzyme recognition and processing of specific polysaccharide substructural stretches, will continue to be imbued in mechanism-based probe designs, extending what has been reported here for linear and branched hemicellulose structures (cellulose versus xyloglucan). This allows the rapid empirical establishment of enzyme specificities in situations where such preferences cannot be gleaned from genomic data alone.

The exquisite specificity of our cyclophellitol-based probe designs compares well to alternative probe designs,^58,59^ leading to relatively simple gel images, with fluorescent bands pointing to probe-reactive proteins that, in all likelihood, feature substrate specifics correlating with that of the probe structure. It should be noted that the suite of probes presented here—indeed probes based on the cyclophellitol scaffold—are reactive toward retaining glycosidases only, excluding inverting glycosidases for identification using our platform. This caveat aside, designing probes targeting *exo*- and *endo*-glycosidases produced to digest different biomass polysaccharides is expected to shed light also in other microbial digestive systems. As well, and as was demonstrated recently, bespoke probes can also be used in machine-learning-assisted, *de novo* glycosidase design.^60^

## Data Availability

The data that support the findings of this study are openly available in the protein databank at https://www.rcsb.org/, reference numbers 8BQA, 8BQB, 8BQC, 8BN7, and 8OZ1.

## Supporting Information Available

Excel sheet of proteomic data (XLSX)
Detailed experimental conditions and methods: enzyme production, structure solution, characterization, biological assays, and organic synthesis (Figures S1–S39 and Tables S1–S4) (PDF)
