# Supplementary material for: A Multiplexing Activity-Based Protein-Profiling Platform for Dissection of a Native Bacterial Xyloglucan-Degrading System
Source: ACS Cent Sci. 2023 Nov 24;9(12):2306–14. doi: 10.1021/acscentsci.3c00831 (PMC10755729; doi:10.1021/acscentsci.3c00831)
Supplement: Supplementary file 2 — oc3c00831_si_002.pdf [file oc3c00831_si_002.pdf]

Supplemental Materials

for

Dissection of a Native Bacterial Xyloglucan-degrading System using Activity-Based Probes

Nicholas G. S. McGregor,[a] Casper de Boer,[b] Quentin P. O. Foucart,[a] Thomas J.M Beenakker,[b] Wendy A Offen,[a] Jeroen D. C. Codée,[b] Lianne I. Willems,[c] Herman S. Overkleeft,*[b] Gideon J. Davies*[a]

[a] York Structural Biology Laboratory, Department of Chemistry, The University of York, Heslington, York, YO10 5DD

[b] Leiden Institute of Chemistry, Leiden University, Einsteinweg 55, 2300 RA Leiden, The Netherlands

[c] York structural Biology Laboratory and York Biomedical Research Institute, Department of Chemistry, University of York, Heslington, York, YO10 5DD, United Kingdom

*Email gideon.davies@york.ac.uk or h.s.overkleeft@lic.leidenuniv.nl

Supplemental Materials and Methods

Recombinant enzyme production

Recombinant forms of BoGH5A, PpXG5, CjCel5D and BoGH31A were produced in and purified from *E. coli* BL21(DE3) as described previously.[1–4] BaCel5A and HiCel7B were produced as described previously.[5–7] Sequences encoding CjCel5C with signal peptide (detected using SignalP[8]) removed and CjCel5B with signal peptide and C-terminal domain removed (amino acids 40-327 in the native sequence) were synthesized by Genscript with codon optimisation for E. coli and were cloned into pET28a between the NcoI and XhoI restriction sites to give a final sequence with a non-cleavable C-terminal 6xHis tag. CjCel5B and CjCel5C were produced in *E. coli* Shuffle T7 Express (NEB) grown in ZYM-505[9] to an OD_{600} of ~0.7 and induced with 0.2 mM IPTG at 25°C overnight. Protein was purified by Histrap purification (GE Healthcare) with an imidazole gradient from 0-500 mM. Protein-bearing elution fractions were pooled, concentrated using a 10 kDa MWCO centrifugal concentrator (Amicon), and purified over a 600x16 mm Superdex 75 column into 20 mM MOPS pH 7.5, 100 mM NaCl (CjCel5B) or 20 mM MOPS pH 7.5, 50 mM NaCl (CjCel5C). Protein was concentrated as above to 10-50 mg/mL. All enzymes were stored frozen at -70°C except CjCel5B which was stored at 4°C due to profound freeze-thaw instability. CjCel5B was fully inactivate by 500 mM ammonium sulfate.

Enzyme crystallisation and diffraction

Crystals were diffracted at Diamond Light Source (Harwell, UK) on beamline I04-1 and automatically processed in space group $P12_11$ (CjCel5D, CjCel5B) or $C222_1$ (CjCel5C) using the Xia2[10] pipeline with DIALS[11]. Computation was carried out using programs from the CCP4 suite[12] unless otherwise stated.

CjCel5D

Initial soaking and co-crystallisation experiments failed to give crystals with clear density in the active site. Thus, CjCel5D was prepared for crystallisation by mixing 21.6 µL of 37 mg/mL enzyme in 50 mM NaPi pH 7.0 with 16.6 µL of ultrapure water and 3.6 µL of 10 mM CB665 in ultrapure water. The reaction was incubated for 3 hours at 30°C to ensure complete labelling. 150 nL of this reaction mixture was then mixed with 150 nL of each well solution from the PACT Premier screen (Molecular Dimensions) and incubated at 4°C. High quality crystals grew from 0.1 M malonate-imidazole-borate buffer (MIB) pH 4.0, 25 % w/v PEG 1500. These were fished and cryo-cooled in LN_2 without cryo-protection.

CjCel5B

Recombinant CjCel5B was concentrated to 46 mg/mL, then diluted to 11.4 mg/mL with ultrapure water to reduce the salinity of the solution just until precipitate was observed. A small amount of precipitate was spun out and the protein solution and the supernatant was mixed with the PEG/Ion and JCSG+ screens at 100:200 nL and 150:150 nL ratios of well solution:protein solution. Plate-cluster crystals grew from 25% PEG3350 with 0.1 M bis-tris 5.5 and from 0.2 M LiCl, 20% PEG3350 at 4°C. The LiCl crystals diffracted best, so were soaked with 2 mM CB665 overnight at RT prior to cryo-cooling in LN_2 without cryo-protection.

CjCel5C

Recombinant CjCel5C (12 mg/mL in 20 mM MOPS pH 7.5, 50 mM NaCl) was mixed with the PEG/Ion and JCSG+ sreens at 100:200 nL and 150:150 nL ratios of well solution:protein solution. Crystals predominantly grew as needle cluster from MPD-containing conditions at 22°C, so an MPD screen (Qiagen) was prepared. Optimal crystals were obtained by mixing 100 nL of diluted seed stock with 600 nL of well solution followed by 900 nL of protein solution. Well solutions containing 4-10% PEG4000, and 25-30% MPD gave high quality crystals. Complexes were obtained by soaking crystals in mother liquor containing 1 mM ligand overnight at 22°C.

Structure solution and refinement

CjCel5D

Diffraction data for CjCel5D bound to CB665 were collected to 1.30 Å. The structure was solved by molecular replacement using Molrep[13] with the known structure (PDBID: 5OYC chain A) as the search model. The resulting solution showed clear density for the bound ligand within the two enzyme active sites in the asymmetric unit. The ligands were built using custom restraints for the linker-modified 4-amino-4-deoxyglucose, and the existing restrains for XYS and YLL with Coot[14], and refined by alternating rounds of manual model building and density refinement using Coot and REFMAC5[15] respectively. All crystal structure figures were generated using Pymol (Schrodinger). Data collection and processing statistics for all structures are given in Supplemental Table 1.

CjCel5B

Diffraction data for unliganded CjCel5B were collected to 1.7 Å and diffraction data for CjCel5B bound to CB665 were collected to 1.7 Å. The structure was solved by molecular replacement using Molrep[13] with the known structure (PDBID: 1TVN chain A) as the search model. The resulting model was refined by alternating rounds of manual model building and density refinement using Coot and REFMAC5[15] respectively.

The CB665-soaked data set was solved by molecular replacement using the refined unliganded structure as a search model. The resulting solution showed clear density for the bound ligand within the two enzyme active sites in the asymmetric unit. The ligands were built as for CjCel5D and refined as above. All crystal structure figures were generated using Pymol (Schrodinger). Data collection and processing statistics for all structures are given in Supplemental Table 1.

CjCel5C

Diffraction data for unliganded CjCel5C were collected to 2.3 Å and diffraction data for CjCel5C bound to CB396 were collected to 2.3 Å. The structure was solved by molecular replacement using Molrep[13] with a homologous structure (PDBID: 4HTY chain A) as the search model. The resulting model was refined by alternating rounds of manual model building and density refinement using Coot and REFMAC5[15] respectively.

The CB396-soaked data set was solved by molecular replacement using the refined unliganded structure as a search model. The resulting solution showed clear density for the bound ligand within the two enzyme active sites in the asymmetric unit. The ligands were built using the existing restrains for BGC and YLL with Coot[14], and refined as above. All crystal structure figures were generated using

Pymol (Schrodinger). Data collection and processing statistics for all structures are given in Supplemental Table 1.

Measuring enzyme activity using fluorogenic substrate

4-methylumbelliferone and 6-chloro-4-methylumbelliferone as well as their derivatives of mono or disaccharides were dissolved at 100 mM in DMSO, then diluted with deionized water to generate working stocks. 4MU-GGGG (Carbosynth), 4MU-XXXG, and 6C4MU-XXXG were dissolved in deionized water at 50 mM and diluted with deionized water. Substrates were stored at -20°C when not in use. Standards of 4-methylumbelliferone and 6-chloro-4-methylumbelliferone (0.1-100 µM) were prepared by dilution using 20 mM HEPES buffer, 100 mM NaCl.

Enzyme samples were diluted with 20 mM HEPES buffer, 100 mM NaCl with 1 mg/mL BSA. Reactions were initiated immediately before reading by the mixing of 20 µL of 2x enzyme solution with 20 µL of 2x substrate solution. Fluorescence was monitored at 25°C using a Clariostar (BMG Labtech) plate reader, measuring in 384-well black plastic plates with a total sample volume of 40 µL, gain settings of 600-1200 V, and $\lambda_{ex}/\lambda_{em}$ of 360nm/450nm. For the determination of k_{cat} and K_M, the final concentration of CjCel5D in each assay was 2.5 ug/mL for 6C4MU-XXXG and 35 ug/mL for 4MU-XXXG and the final concentration of PpXG5 was 1 ug/mL for both substrates. BoGH5 was used at a final concentration of 0.2 ug/mL for 6C4MU-XXXG and 35 ug/mL for 4MU-XXXG.

Measuring irreversible inhibition kinetics

Fast irreversible inhibition kinetics were measured using a continuous assay as described previously.[16] Briefly, solutions of inhibitor at variable concentrations were prepared in 20 mM HEPES pH 7.5 buffer, 100 mM NaCl, 1 mg/mL BSA with 100 µM substrate. Solutions of enzyme (1 µg/mL, 0.4 µg/mL, and 5 ng/mL for BaCel5A, CjCel5B, and Hi Cel7B, respectively) were prepared in 20 mM HEPES pH 7.5 buffer, 100 mM NaCl, 1 mg/mL BSA and 20 µL of enzyme solution was added to each inhibitor+substrate solution. Fluorescence was immediately monitored for 2 hours at 25°C. F vs t plots were fit with an exponential decay model to determine apparent decay constants (k_{app}):

$$F = F_0 + Ce^{k_{app}t}$$

k_{app} values were then plotted against inhibitor concentration and fit with a site-saturation model modified to account for competition from the substrate:

$$k_{app} = \frac{k_i[I]}{K_I\left(1 + \frac{[S]}{K_{M,S}}\right) + [I]}$$

Where no inflection in the k_{app} vs [I] plot was observed, a straight-line fit with forced zero was performed. In this case, the model simplified to:

$$k_{app} = [I]\frac{k_i}{K_I\left(1 + \frac{[S]}{K_{M,S}}\right)}$$

The inhibitor performance constant (k_i/K_I) was subsequently estimated using the independently measured values of [S] and K_M for the substrate-enzyme interaction ($K_{M,S}$).

Slow irreversible inhibition kinetics were measured using a residual activity assay as described previously.[17] Briefly, solutions of enzyme (4, 20, and 40 µg/mL for BoGH5, CjCel5D, and PpXG5, respectively) in pH 7.5 HEPES, 100 mM NaCl, 1 mg/mL BSA were prepared. A series of inhibitor solutions were prepared at 20 µL volume and mixed with 20 µL of enzyme solution. Each reaction was incubated at 30°C in a sealed PCR tube. 5 µL samples were taken at various time points and diluted into 95 µL of assay solution (50 µM substrate in pH 7.5 HEPES buffer, 100 mM NaCl, 1 mg/mL BSA). Activity was immediately monitored for 3 minutes at 25°C. F vs t plots were fit with straight lines to extract rates in F/s. Rates were calibrated to µM/s using a standard curve of the appropriate chromophore leaving group in assay solution. Rates were normalised to a no-inhibitor negative control to account for spontaneous loss of enzyme activity over long incubations. Rate vs. time plots at each concentration of inhibitor were fit to an exponential decay model to extract k_{app} values that were analyzed as above.

Activity-based protein profiling by in-gel fluorescence

Each fluorescent probe was dissolved in DMSO to prepare a 5 mM stock solution which was diluted with ultrapure water. Proteins were separated at 200 V using either a 4-20% Criterion TGX SDS-PAGE gel (Bio-Rad) or 1 mm MiniProtean 10% SDS-PAGE gel. Fluorescence was imaged using a Typhoon 5 laser scanner with the Cy5, Cy3, and Cy2 laser and filter sets. Enzyme molecular weights were estimated using a Pageruler 10-180 kDa pre-stained protein ladder.

To prevent overloading of the SDS-PAGE gel, 1 volume of each lysate sample was diluted with 9 volumes of carbon-free MOPS minimal medium prior to labelling. Staining of diluted lysates and undiluted secretomes with fluorescent ABPs was done using a mixture of 60 µM each of CB644, JJB376, and CB664. 0.2 volumes of the probe mixture were combined with 1 volume of sample and incubated for 30 minutes at 30°C. 0.4 volumes of 4x SDS-PAGE loading dye were then added and the samples were heated to 95°C for 2 minutes, then rapidly cooled to 20°C prior to running SDS-PAGE.

Measuring polysaccharide hydrolysis

Enzyme activity was measured using polysaccharides purchased from Megazyme international (Bray, Ireland) including tamarind xyloglucan (tXyG), carboxymethylcellulose (CMC), low viscosity barley mixed-linkage glucan (bMLG), konjac glucomannan (kGM), low viscosity carob galactomannan (cGM), and low viscosity wheat arabinoxylan (wAX). With the exception of CMC, each polysaccharide was prepared at 1% w/v by initial wetting with ethanol followed by dispersion in deionized water and gentle boiling for 5 minutes to dissolve the material and evaporate ethanol. CMC was prepared at 1% by rapid addition of the powder to vigorously stirred deionized water followed by 30 minutes of stirring at RT.

Typically, a 2x enzyme solution in 100 mM buffer was prepared and 1 volume of this was mixed with 1 volume of 2 mg/mL substrate. The reaction was incubated at 30°C for 15 minutes, then quenched by the addition of 2 volumes of BCA reagent (2.5 mM bicinchoninic acid, 1.25 mM CuSO4, 2.5 mM L-serine, 0.4 M sodium carbonate buffer pH 11.25). Reactions were developed by incubation at 80°C and transferred to a 384-well plate to read A_{563}. Reducing ends were quantified using a glucose

calibration (0-200 µM) following subtraction of an enzyme-free control. For enzyme concentrations above 5 µg/mL, a heat-killed enzyme+substrate blank was prepared to account for measurable background BCA signal generated by the protein.

Culturing *Cellvibrio japonicus*

C. japonicus Ueda107 was ordered from the National Collection of Industrial, Food and Marine Bacteria (NCIMB #10462). The cells were initially resuspended in trypticase soy broth and streaked on trypticase soy agar. Small (<1 mm) isolated colonies were visible on the agar following 2-3 days of growth at 37°C. *C. japonicus* was routinely streaked on minimal MOPS agar containing 0.25% glucose and grown at ambient temperature (~22°C) for 3 days to develop >1 mm colonies, which develop as pits in the agar due to the organism's agarolytic nature. Liquid culturing was performed in minimal MOPS medium supplemented with different carbon sources as previously described.[18] For cell storage, 1 mL of saturated MOPS-glucose culture was mixed with 500 µL of 80% glycerol and frozen at -80°C. Complete MOPS medium was prepared freshly each week as *C. japonicus* growth noticeably slowed (and eventually stopped) as the medium aged at 4°C. All culture handling was performed in a laminar flow hood to prevent contamination.

Minimal MOPS medium was prepared as follows: 5000x micronutrient stock was prepared containing 9 mg ammonium molybdate, 62 mg boric acid, 18 mg cobalt chloride, 6 mg cupric sulphate, 40 mg manganese chloride, 7 mg zinc sulphate in 50 mL of ultrapure water and filter-sterilising. 100x phosphate stock was prepared by dissolving 1.15 g of K_2HPO_4 in 50 mL of water and filter-sterilising. 10x MOPS stock was prepared by combining 41.8 g of MOPS free acid with 3.6 g of tricine 400 mL of ultrapure water. The pH was brought to 7.35-7.45 with solid KOH (4-5 g). To this was added $FeSO_4.7H_2O$ (14 mg), NH_4Cl to 90 mM (2.4 g), K_2SO_4 to 2.75 mM (240 mg), $CaCl_2$ to 0.005 mM (2.5 uL of a 1 M stock), $MgCl_2$ to 5.25 mM (2.62 mL of a 1 M stock), NaCl to 500 mM (14.6 g). This was filter-sterilised and stored at -20°C in aliquots. Carbon sources were prepared at various concentrations in ultrapure water and autoclaved to sterilise.

Complete medium was prepared by combining ultrapure water, freshly thawed 10x MOPS stock, 100x phosphate stock, and 5000x micronutrient stock with the required sterile carbon source in a sterile container. "Primed" cells were prepared by inoculation of 50-100 mL of 0.25% glucose medium in a 250 mL baffled flask with a single colony of *C. japonicus*. This was incubated for ~20 hours at 30°C with 200 RPM shaking to reach a final carbon-limited saturation OD_{600} of between 1.35 and 1.45.

Preparation of *C. japonicus* cells, cell lysates and secretomes from growth on different carbon sources

50 mL *C. japonicus* cultures were prepared in 250 mL baffled flasks supplemented with no carbon, glucose, cellobiose, tXyG, wAX, or cGM. Each culture was inoculated with naïve cells (grown in tryptic soy broth) to an initial OD_{600} of 0.0015 and shaken for 20 hours at 200 RPM to reach saturation. No growth was observed in the no carbon control. The OD_{600} of each culture was found to be between 1.37 and 1.44 with the exception of the wAX culture which had a terminal OD600 of 0.55. Cells were collected by centrifugation at 4000xg for 30 minutes at 4°C. Supernatants were 0.2 µm-filtered to

yield “secretome” samples. Secretome samples were stored at 4°C due to ABPP-revealed freeze-thaw instability of some enzyme activities.

The cells were resuspended in 2 mL of carbon-free MOPS minimal medium, 0.5 mL was set aside, and the remaining 1.5 mL was transferred to an Eppendorf tube. Cells were collected by centrifugation at 12000xg for 2 minutes. The resulting cell pellet was then resuspended in 1.5 mL of lysis reagent (1x BugBuster detergent (Millipore), 125 U/mL Benzonase (Millipore), 0.01 mg/mL lysozyme, 20 mM NaPi pH 7.5 buffer) and incubated for 15 minutes at 30°C. The translucent yellow solution was then spun down at 12000xg for 5 minutes, yielding a small off-white pellet and the supernatant was collected as the “lysate” sample (roughly 10 mg/mL total protein). Lysate samples were used fresh initially for 1 day (stored at 4°C), then stored in 100 µL aliquots at -20°C for follow-up experiments where concordance with fresh observations could be assessed, or enzymatic activity was not relevant (e.g. total sample trypsinization prior to LC-MS/MS).

Glycoside hydrolase induction

For variable [substrate] experiments 10 µL of primed cells were diluted into 90 µL of 1.11x inducing medium (MOPS minimal medium supplemented with 0.001% glucose and different concentrations of other carbon sources) in a 1.5 mL Eppendorf tube. Following incubation for 2 hours at 30°C, 10 µL of culture was mixed with 10 µL of 2x lysis reagent (2x BugBuster, 250 U/mL Benzonase, 0.02 mg/mL lysozyme, 40 mM NaPi pH 7.5) and supplemented with 10 µL of 3x probe mixture. Following 30 minutes of incubation at 30°C, the samples were analysed as described in the “Activity-based protein profiling by in-gel fluorescence” section. For time course experiments, 200 µL cultures were prepared with 0.1% substrate, sampled over time, and processed as above.

Investigating the impact of BoGH31A activity on xyloglucanase labelling

Solutions containing BoGH31 at 100, 1 or 0 µg/mL were prepared in 50 mM NaPi pH 7.0 containing 10 µM of either CB664 and CB477 were added. These reactions were incubated for 1 hour at 37°C. The probe+BoGH31 solutions were then mixed with CjCel5D to a final concentration of 10 µg/mL and incubated for a further 1 hour at 37°C. The resulting protein mixture was prepared for SDS-PAGE as above, then run on a 10% SDS-PAGE gel, and imaged for cy5 fluorescence. Bands were integrated using ImageQuant (GE Healthcare).

LC-MS analysis of ABP-XyG following BoGH31A treatment

Solvents were purchased from Sigma at LC-MS grade. Samples containing 10 µM of CB665 that had been treated with BoGH31A were analyzed using an Acquity iClass UPLC system (Waters) coupled to a MaXis HD (Bruker) QToF mass spectrometer. Separations were performed using a 1x150 mm 3µm particle Hypercarb porous graphitic carbon column (Thermo) operating at 0.1 mL/min at 60°C. Mobile phase A was 0.1% ammonium hydroxide (LC-MS grade, Fluka #4427310) in water and mobile phase B was 0.1% ammonium hydroxide in 80:20 acetonitrile:water. The gradient program was 0-5 min: 2-5% B, 5-20 min: 5-60% B, 20-21.5 min: 100% B, 21.5-22 min: 100-2% B, 22-25 min: 2% B. 1 µL of sample was injected in each run. MS detection was performed in positive mode, scanning from 300-2000 m/z. Analysis was performed to identify peaks corresponding to the m/z values (±0.05) of sodium adducts of CB665 (707.26 m/z) and hydrolyzed CB665 (725.28 m/z), as well as derivatives of these missing a xylose residue (-132.05 m/z), or missing a cyclophellitol residue (-176.05 m/z).

Intact protein mass spectrometry

Intact mass spectrometry (MS) was then performed with online desalting chromatography as described previously.[16]

For covalent labelling experiments, enzyme samples in buffer (100 mM NaPi, pH 7.5) were mixed with inhibitor (0.1 mM) and incubated at 20°C. At various time points, samples were diluted 5-fold into 10% acetonitrile with 1% formic acid to stop the labelling reaction.

Purification of native CjCel5B

100 mL of MOPS medium supplemented with 0.25% wheat arabinoxylan (Megazyme) was inoculated with 1 mL of *C. japonicus* starter culture. The culture was incubated overnight at 30°C with shaking. Cells were removed by centrifugation at 4000xg for 30 minutes at 4°C followed by filtration using a 0.45 µm cut-off filter. The isolated 100 mL of secretome was concentrated down to ~400 µL via centrifugal ultrafiltration with a 30 kDa MWCO PES filter. The protein concentrate was diluted with 2 mL of 20 mM MOPS buffer, pH 7.5 to reduce the ionic strength, and was loaded onto a HiScreen Q HP anion exchange column (GE Healthcare). Following a 1 CV wash, proteins were eluted using a 5 CV gradient from 0-200 mM NaCl. UV-active 2 mL fractions were collected. 10 uL of each fraction was stained with Cy5-ABP-XyG and analysed by SDS-PAGE for overall purity and ABP-XyG-reactive enzyme content. The second protein-bearing fraction was taken forward for additional analysis due to its suitable protein concentration and reasonable purity.

Monosaccharide analysis of CjCel5B

4 ug of CjCel5B in 50 µL of 20 mM MOPS, ~50 mM NaCl, pH 7.5 was supplemented with neat trifluoroacetic acid (TFA) to a final concentration of 2 M and incubated at 95°C for 3 hours to hydrolyze sugars. The sample was then dried in a speedvac at 40°C. The sample was dissolved in 50 µL of deionized water. The HPAEC-PAD system consists of a Dionex DX 600 equipped with an ED50 electrochemical detector with a gold working electrode, GP50 gradient pump, LC30 chromatography oven, and AS40 automated sampler (Dionex Corporation, Sunnyvale, CA). HPAEC-PAD was performed on a CarboPac PA-1 column using 5 µL injections with a column temperature of 30°C. Monosaccharides were separated using an isocratic flow of 20 mM NaOH at 0.5 mL/min. Standards were prepared and analyzed containing 200 µM galactose, glucose, mannose, or arabinose.

Glycoprotein SDS-PAGE analysis

C. japonicus lysates were prepared as above, following growth on glucose, cellobiose, xyloglucan, or xylan. An *E. coli* lysate was prepared in the same manner as negative control. 5 µL of each lysate was diluted into 45 µL of ultrapure water to prevent overloading. Purified Cel5B was not diluted. 15 µL of each sample was mixed with 5 µL of 4x SDS-PAGE loading dye and heated to 95°C for 2 minutes. Following SDS-PAGE separation of the entire 20 µL sample, the gel was stained using the Pro-Q Emerald gel staining kit (Invitrogen) following the manufacturer's instructions. The gel was imaged for Pro-Q stain with an iBright gel imager (ex 280 nm, em 530 nm). The gel was then stained with SYPRO ruby (Invitrogen) and imaged again (ex 450 nm, em 650 nm). The gel was then stained with Coomassie dye and imaged with white light.

CjXyl31A Labelling Experiments Method

For each reaction, 1 µl *Cj*Xyl31A at 10 µM in 10 mM MES pH 6.5, 150 mM NaCl was diluted with 9 µl McIlvaine buffer pH 6, 100 mM NaCl. A 1 mM solution of CB693 in DMSO was diluted with water to 1, 2.5, 5, 10, 20, 40 and 100 µM, and 1 µl of each were added to a 10 µl reaction sample. The reactions were shaken at 300 rpm, at 37 °C for 1 hour. The reactions were stopped by adding 3 ul 4 x SDS PAGE sample buffer and boiling for 5 min. The samples were loaded onto a Biorad 4 – 20% Mini-PROTEAN TGX gel, with New England Biolabs pre-stained broad range markers (catalogue number P7719). The gel was run at 200 V for 45 min, and then imaged gel on an Amersham™ Typhoon™ 5 imager and subsequently stained with Coomassie blue stain. For the pH range labelling, the same procedure was used, but with 1 µl 10 uM *Cj*Xyl31A in 10 mM MES pH 6.5, 150 mM NaCl diluted with 9 µl McIlvaine buffer with 100 mM NaCl, at pHs 4, 5, 6, 7, 8 and 9, and with 1 µl 100 µM CB693 added.

For the *Cj*Xyl31A-doped lysate samples, lysate was prepared as follows: 10 ml Luria broth was inoculated with 5 µl of *E. coli* BL21 (DE3) GOLD cells and incubated overnight at 37 °C whilst shaking at 180 rpm. 3.6 ml of culture were spun down at 13,000 rpm for 5 min, and the following added: 100 µl BugBuster Protein Extraction Reagent (Millipore), 260 µl McIlvaine buffer pH 6 with 100 mM NaCl, 16 µl cOmplete Mini EDTA-free Protease inhibitor cocktail tablet (Roche) dissolved in 1 ml water, and AEBSF to 1 mM. The pellets were vortexed, left for 5 min, and vortexed again. The suspension was centrifuged at 13,000 rpm for 5 min, and the supernatant used for doping with *Cj*Xyl31A as follows: Doped samples were made by combining 1 µl *Cj*Xyl31A at concentrations of 0, 0.1, 0.5, 1,2, 4, 6, 8 and 10 µM (diluted as above), 6 µl lysate supernatant, 3µl McIlvaine buffer pH 6 with 100 mM NaCl and 1 µl 100 µM CB693. The doped samples were analysed as above.

Biotin-streptavidin enzyme enrichment and proteomic analysis

In a 1.5 mL lo-bind eppendorf tube, biotinylated probe was added to 50 µL of *C. japonicus* glucose-grown, cellobiose-grown or xyloglucan-grown lysate (undiluted) or wAX-grown secretome (20-fold concentrated using a 30 kDa MWCO centrifugal concentrator (Vivaspin) to a final concentration of 10 µM. A no probe control was also prepared for each sample. The reactions were incubated for 1 hour at 30°C. Samples were then denatured and reduced by the addition of 5.5 µL of 10x denaturing buffer (40 mM DTT, 2% SDS) and heating to 95°C for 2 minutes. Some precipitation was observed from lysate samples; this was not removed. 10 µL of 0.1 M IAA was then added and the samples were incubated in the dark for 30 minutes at RT. from lysate samples; this was not removed. 10 µL of 0.1 M IAA was then added and the samples were incubated in the dark for 30 minutes at RT. The samples were then diluted to 100 µL total volume with ultrapure water and MeOH-$CHCl_3$ extraction was performed as described previously.[19] Due to the low protein concentration (~0.075 mg/mL by A_{280}) protein was recovered from the wAX-grown secretome via acetone precipitation as described previously.[19] Biotinylated, reduced, alkylated, and extracted protein pellets were dissolved in 100 µL of 10 M urea at RT, then diluted with 900 µL of 0.05% SDS in 20 mM NaPi pH 7.5. 15 µL of resuspended Streptavidin Mag Sepharose beads (GE Healthcare) were added to each sample and incubated at 25°C with vigorous shaking for 1 hour. Beads were then pulled down using a magnetic rack. The beads were washed with 1 mL of 2% SDS at RT, then 1 mL of 2% SDS at 65°C for 10 minutes, then 1 mL of 2 M Urea at RT, then 1 mL of ultrapure water twice. The beads were finally resuspended in 20 µL of 50 mM triethylammonium bicarbonate (TEAB) buffer (pH 8.5) and supplemented with 1

µL of 0.5 µg/µL sequencing grade trypsin (Promega). The bead trypsinization was incubated with shaking overnight at 37°C. Beads were pulled down and the 20 µL peptide-bearing supernatant was transferred to a PCR tube. 2 µL of 20 mg/mL TMT^2-126 labelling reagent in absolute ethanol (ThermoFisher) was added to each no probe control and 2 µL of 20 mg/mL TMT^2-127 in absolute ethanol was added to each probe-labelled peptide solution. Labelling reactions were incubated for 1 hour at RT, then quenched by the addition of 1 µL of 5% hydroxylamine (~65 mM final) and incubated at RT for 15 minutes. 10 µL of labelled peptide solution from the no probe control and the probe-labelled sample were mixed together.

Whole proteome samples were prepared from 20 µL of *C. japonicus* glucose-grown, cellobiose-grown or xyloglucan-grown lysate by MeOH-$CHCl_3$ extraction followed by dissolution in 1 M urea with 50 mM TEAB and trypsinization as described previously.[19] A secretome sample was prepared from 20 µL of concentrated wAX-grown secretome in the same manner, but using acetone precipitation.

Peptide Liquid Chromatography and Mass Spectrometry

Peptides were loaded onto an mClass nanoflow UPLC system (Waters) equipped with a nanoEaze M/Z Symmetry 100 Å C18, 5 µm trap column (180 µm x 20 mm, Waters) and a PepMap, 2 µm, 100 Å, C18 EasyNano nanocapillary column (75 µm x 500 mm, Thermo). The trap wash solvent was aqueous 0.05% (v:v) trifluoroacetic acid and the trapping flow rate was 15 µL/min. The trap was washed for 5 min before switching flow to the capillary column. Separation used gradient elution with two solvents: solvent A, aqueous 1% (v:v) formic acid; solvent B, acetonitrile containing 1% (v:v) formic acid. The flow rate for the capillary column was 300 nL/min and the column temperature was 40°C. The linear multi-step gradient profile was: 3-10% solution B over 7 mins, 10-35% B over 85 mins, 35-99% B over 10 mins and then proceeded to wash with 99% solvent B for 4 min, before returning to 3 % solvent B and equilibrating for 14 mins. The nanoLC system was interfaced with an Orbitrap Fusion hybrid mass spectrometer (Thermo) with an EasyNano ionisation source (Thermo).

Positive ESI-MS, MS^2 and MS^3 spectra were acquired using Xcalibur software (version 4.0, Thermo). Instrument source settings were: ion spray voltage, 1,900 V; sweep gas, 0 Arb; ion transfer tube temperature; 275°C. MS^1 spectra were acquired in the Orbitrap with: 120,000 resolution, scan range: *m/z* 380-1,500; AGC target, 2e^5; max fill time, 50 ms. Data dependent acquisition was performed in top speed mode using a 4 s cycle, selecting the most intense precursors with charge states >1. Dynamic exclusion was performed for 50 s post precursor selection and a minimum threshold for fragmentation was set at 3e^4. MS^2 spectra were acquired in the linear ion trap with: scan rate, turbo; quadrupole isolation, 1.2 *m/z*; activation type, CID; activation energy: 35%; AGC target, 1e^4; first mass, 120 *m/z*; max fill time, 50 ms. MS^3 spectra were acquired in multi notch synchronous precursor mode (SPS^3), selecting the 5 most intense MS^2 fragment ions between 400-1,000 *m/z*. SPS^3 spectra were measured in the Orbitrap mass analyser using: 50,000 resolution, quadrupole isolation, 2 *m/z*; activation type, HCD; collision energy, 65%; scan range: *m/z* 110-500; AGC target, 5e^4; max fill time, 86 ms. Acquisitions were arranged by Xcalibur to inject ions for all available parallelizable time.

Peak lists were converted from centroided .raw to .mgf format using Mascot Distiller (version 2.6.1, Matrix Science) and MS^3 spectra were concatenated into their parent MS^2 spectra for database searching.

Synthesis of cyclophellitol derivatives

General chemical synthesis procedures

All reactions were carried out in oven-dried glassware. Trace amounts of water were removed by co-evaporation with toluene. Reactions were carried out under an atmosphere of nitrogen unless stated otherwise. Tetrahydrofuran (THF), *N*,*N*-dimethylformamide (DMF) dichloromethane (DCM) and toluene were of reagent grade and were stored over molecular sieves before use. Pentane, petroleum ether and diethyl ether used for workup and column chromatography were of technical grade and used as received. Ethyl acetate (EtOAc) was distilled under reduced pressure before use. Unless stated otherwise, solvents were removed by rotary evaporation under reduced pressure at 40°C. All other chemicals (Acros, Sigma-Aldrich, TCI, Carbosynth, Merck, Boom, Honeywell & Biosolve) were used as received. Reactions were monitored by TLC analysis using Merck aluminum sheets (Silica gel 60 F254) with detection by UV absorption (254 nm) and by spraying with a solution of $(NH_4)_6Mo_7O_{24}\cdot 4H_2O$ (25 g/L) and $(NH_4)_4Ce(SO_4)_4\cdot 2H_2O$ (10 g/L) in 10% sulfuric acid or a solution of $KMnO_4$ (20 g/L) and K_2CO_3 (10 g/L) in water, followed by charring at ~150 °C. Silica gel column chromatography was performed on Screening Devices silica gel 60 (particle size of 40 – 63 µm, pore diameter of 60 Å). For reversed-phase HPLC purifications an Agilent Technologies 1200 series instrument equipped with a semi-preparative column (Gemini C18, 250 x 10 mm, 5 µm particle size, Phenomenex) was used. ^1H and ^{13}C NMR spectra were recorded on a 300/75, 400/100, 500/125, 600/150 or 850/200 MHz spectrometer. Chemical shifts (δ) are given in ppm relative to tetramethylsilane or the residual solvent. Coupling constants are given in Hz. High-resolution mass spectrometry (HRMS) analysis was performed with a LTQ Orbitrap mass spectrometer (Thermo Finnigan), equipped with an electrospray ion source in positive mode (source voltage 3.5 kV, sheath gas flow 10 mL/min, capillary temperature 250°C) with resolution R = 60000 at m/z 400 (mass range m/z = 150 – 2000) and dioctyl phthalate (m/z = 391.28428) as a "lock mass". The mass spectrometer was calibrated prior to measurements with a calibration mixture (Thermo Finnigan).

α-Xylose activity-based probes

***N*-8-azidooctyl-2,3-di-*O*-benzyl-D-xylose-cyclophellitol aziridine (28)**

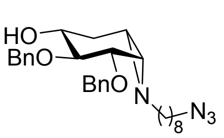

2,3-di-*O*-benzyl-D-xylose-cyclophellitol aziridine[20] **27** (0.15 g, 0.46 mmol) was dissolved in DCM (1.8 ml). The solution was cooled to 0°C and DIPEA (0.12 ml, 0.69 mmol) and freshly prepared 8-azidooctyl trifluoromethanesulfonate[29] (1M in DCM, 0.55 ml, 0.55 mmol) were added. The reaction was slowly warmed to room temperature and stirred for 21 hours. MeOH (2 ml) was added and the mixture was stirred for 2 hours. Toluene was added and the mixture was evaporated to dryness. Column chromatography (DCM/MeOH, 1/0 -> 99/1, v/v) afforded the product as an oil. (0.19 g, 0.40 mmol, 86%)

^1H NMR (500 MHz, $CDCl_3$) δ 7.40 – 7.22 (m, 10H, benzyl), 4.85 (br, 1H, OH), 4.70 (d, *J* = 12.2 Hz, 1H, CH_2Bn), 4.65 (d, *J* = 12.2 Hz, 1H, CH_2Bn), 4.62 (d, *J* = 11.7 Hz, 1H, CH_2Bn), 4.50 (d, *J* = 11.7 Hz, 1H, CH_2Bn), 3.83 (dd, *J* = 4.7, 3.4 Hz, 1H, H2), 3.73 (br, 1H, H4), 3.55 (dd, *J* = 5.6, 3.4 Hz, 1H, H3), 3.21 (t, *J* = 7.0 Hz, 2H, CH_2N_3), 2.39 (dt, *J* = 11.5, 6.8 Hz, 1H, CH_2N aziridine), 2.14 – 2.05 (m, 2H, H5a/CH_2N aziridine), 1.97 (ddd, *J* = 14.3, 5.3, 1.8 Hz, 1H, H5b), 1.88 (dd, *J* = 6.3, 4.7 Hz, 1H, aziridine), 1.82 – 1.76 (m, 1H, aziridine), 1.60 – 1.50 (m, 4H, CH_2 spacer), 1.41 – 1.23 (m, 8H, CH_2 spacer).

^{13}C NMR (126 MHz, $CDCl_3$) δ 138.5, 138.3, 128.4, 128.3, 128.2, 128.1, 127.9, 127.7, 127.6, 127.6, 79.7 (C3), 76.6 (C2), 72.6 (CH_2Bn), 70.7 (CH_2Bn), 68.3 (H4), 60.4 (CH_2N aziridine), 51.4 (CH_2N_3), 39.7 (aziridine), 38.2 (aziridine), 29.5, 29.4, 29.0, 28.8, 27.1, 26.8 (C5), 26.6 (spacer). HRMS (ESI) m/z: $[M+H]^+$ calculated for $C_{28}H_{39}N_4O_3$ 479.3014, found 479.3017.

_N_-8-aminooctyl-2,3-di-_O_-benzyl-D-xylose-cyclophellitol aziridine (S1)

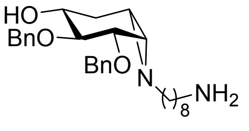

Azide **28** (0.189 g, 0.396 mmol) was dissolved in MeCN (7.9 ml). H_2O (71 µl, 3.96 mmol) and PPh3 polymer bound (3 mmol/g, 0.264 g, 0.792 mmol) were added and the mixture was stirred at 70°C for 13 hours. H_2O (0.5 ml) was added and the mixture was stirred for 4.5 hours at the same temperature. The solids removed by filtration, volatiles were removed under reduced pressure and the product was used and analyzed without further purification. (0.171 mg, 0.378 mmol, 95%)

^{1}H NMR (500 MHz, $CDCl_3$) δ 7.41 – 7.24 (m, 10H), 4.71 (d, *J* = 12.2 Hz, 1H, CH_2Bn), 4.66 (d, *J* = 12.2 Hz, 1H, CH_2Bn), 4.63 (d, *J* = 11.7 Hz, 1H, CH_2Bn), 4.51 (d, *J* = 11.7 Hz, 1H, CH_2Bn), 3.84 (dd, *J* = 4.7, 3.5 Hz, 1H, H2), 3.73 (q, *J* = 5.2 Hz, 1H, H4), 3.55 (dd, *J* = 5.6, 3.5 Hz, 1H, H3), 2.65 (t, *J* = 7.0 Hz, 2H, C**H_2**NH_2), 2.41 (dt, *J* = 11.5, 6.9 Hz, 1H, CH_2N aziridine), 2.13 – 2.05 (m, 2H, CH_2N aziridine/H5a), 2.02 – 1.94 (m, 1H, H5b), 1.88 (dd, J = 6.3, 4.7 Hz, 1H, aziridine), 1.83 – 1.77 (m, 1H, aziridine), 1.60 – 1.52 (m, 2H), 1.46 – 1.22 (m, 10H).

^{13}C NMR (126 MHz, CDCl3) δ 138.6, 138.4, 128.5, 128.4, 127.8, 127.7, 127.6, 79.9 (C3), 76.7 (C2), 72.8 (CH2Bn), 70.9 (CH2Bn), 68.4 (C4), 60.6 (CH2N aziridine), 42.3 (CH2NH2), 39.9 (aziridine), 38.3 (aziridine), 33.8, 29.6, 29.6, 29.4, 27.3, 27.0, 26.9. HRMS (ESI) m/z: [M+H]+ calculated for C28H41N2O3 453.3112, found 453.3112

_N_-8-aminooctyl-D-xylose-cyclophellitol aziridine (29)

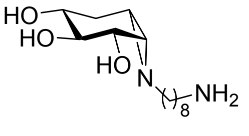

Ammonia (20 ml) was condensed and kept at -60°C. Sodium (0.26 g, 11.5 mmol) was added and stirred for 10 minutes. Benzyl protected **S1** (0.171 g, 0.382 mmol) dissolved in *t*-BuOH (0.35 ml, 3.68 mmol) and THF (5 ml) was slowly added to the blue solution. The color disappeared immediately so more sodium (85 mg, 3.7 mmol) was added. The blue solution was stirred for 35 minutes. Water was slowly added and the mixture was evaporated. The residue was dissolved in water and eluted over a short column of amberlite CG50 (NH_4^+) with 0.5 M NH_4OH. The combined fractions were concentrated under reduced pressure providing the product as an oil. (105 mg, 0.386 mmol quant.)

^{1}H NMR (500 MHz, MeOD) δ 3.70 (dd, *J* = 7.5, 4.0 Hz, 1H, H2), 3.35 – 3.25 (m, 2H, H3/H4), 2.81 – 2.72 (m, 2H, C**H_2**NH_2), 2.35 – 2.24 (m, 2H, H5a/CH_2N aziridine), 2.15 (ddd, *J* = 11.7, 8.5, 6.4 Hz, 1H, CH_2N aziridine), 1.84 (dd, *J* = 6.5, 4.0 Hz, 1H, aziridine), 1.69 (td, *J* = 6.6, 1.2 Hz, 1H, aziridine), 1.67 – 1.53 (m, 5H, H5b/CH_2 spacer), 1.42 – 1.31 (m, 8H, CH_2 spacer).

^{13}C NMR (126 MHz, MeOD) δ 76.2 (H3), 73.6 (H2), 71.3 (H4), 61.9 (CH_2N aziridine), 45.7 (aziridine), 41.6 (CH_2NH_2), 38.3 (aziridine), 32.6 (C5), 31.0, 30.5, 30.5, 30.3, 28.3, 27.6. HRMS (ESI) m/z: $[M+H]^+$ calculated for $C_{14}H_{29}N_2O_3$ 273.2172, found 273.2173.

Biotin-D-xylose-cyclophellitol aziridine (30, ABP-aXyl-Bio)

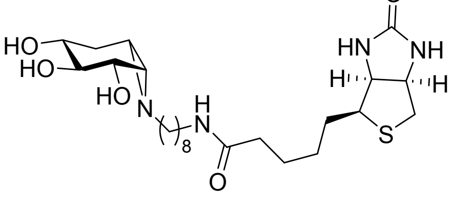

Amine **29** (6.5 mg, 24 µmol) was dissolved in DMF (0.24 ml) and to the solution was added Biotin (6.5 mg , 26 µmol). DIPEA (6.3 µl, 36 µmol), DMAP (cat.) and DIC (6.8 µl ,43 µmol) were added and the mixture was stirred overnight. LC-MS indicated conversion and the product was purified on semi-preparative HPLC eluting with a linear gradient of solution A (MeCN) in solution B (50mM NH_4HCO_3 in H_2O). The fractions were concentrated under reduced pressure, co-evaporated with water, diluted with water and lyophilized to yield the product as a white solid. (2.20 mg, 4.40 µmol, 18%)

^{1}H NMR (500 MHz, MeOD) δ 4.49 (dd, *J* = 7.8, 5.0 Hz, 1H), 4.30 (dd, *J* = 7.9, 4.4 Hz, 1H), 3.70 (dd, *J* = 7.5, 4.0 Hz, 1H), 3.29 – 3.25 (m, 2H), 3.23 – 3.18 (m, 1H), 3.16 (td, *J* = 7.0, 1.9 Hz, 2H), 2.93 (dd, *J* = 12.8, 5.0 Hz, 1H), 2.71 (d, *J* = 12.7 Hz, 1H), 2.33 – 2.25 (m, 2H), 2.22 – 2.12 (m, 3H), 1.84 (dd, *J* = 6.5, 4.0 Hz, 1H), 1.65 (tddd, *J* = 26.9, 21.2, 13.2, 6.4 Hz, 8H), 1.46 (dp, *J* = 23.2, 7.8, 7.4 Hz, 4H), 1.34 (s, 8H).

^{13}C NMR (126 MHz, MeOD) δ 176.0, 76.2, 73.6, 71.3, 63.4, 62.0, 61.6, 57.0, 45.7, 41.1, 40.4, 38.3, 36.8, 32.6, 30.6, 30.5, 30.4, 30.4, 29.8, 29.5, 28.4, 28.0, 27.0. HRMS (ESI) m/z: $[M+H]^+$ calculated for $C_{24}H_{43}N_4O_5S$ 499.2947, found 499.2949.

Cy5-D-xylose-cyclophellitol aziridine (31, ABP-aXyl-Cy5)

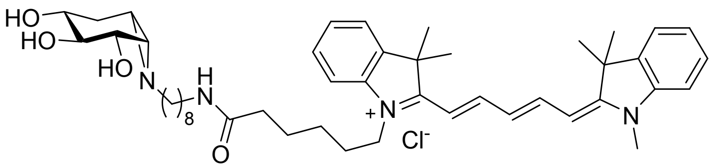

Amine **29** (49 mg, 0.18 mmol) was dissolved in DMF (0.25 ml). DIPEA (76 µl, 0.44 mmol), Cy5COOH (94 mg, 0.18 mmol) and PyBOB (0.10 g, 0.20 mmol) were added and the mixture was stirred overnight. LC-MS indicated conversion and the product was purified on semi-preparative HPLC eluting with a linear gradient of solution A (MeCN) in solution B (50mM NH_4HCO_3 in H_2O). The fractions were concentrated under reduced pressure, co-evaporated with water, diluted with water and lyophilized to yield the product as a blue solid. (17.4 mg, 0.034 mmol, 19%)

^1H NMR (500 MHz, CD_3CN) δ 8.08 (t, *J* = 13.1 Hz, 2H), 7.47 (d, *J* = 7.4 Hz, 2H), 7.43 – 7.37 (m, 2H), 7.28 – 7.21 (m, 4H), 6.65 (d, *J* = 5.8 Hz, 1H), 6.55 (t, *J* = 12.4 Hz, 1H), 6.21 (dd, *J* = 21.1, 13.8 Hz, 1H), 4.00 (t, *J* = 7.5 Hz, 2H), 3.58 (dd, *J* = 7.4, 3.8 Hz, 1H), 3.54 (s, 3H), 3.24 (td, *J* = 9.4, 6.7 Hz, 1H), 3.18 – 3.13 (m, 1H), 3.07 (q, *J* = 6.6 Hz, 2H), 2.20 – 2.07 (m, 4H), 1.77 (p, *J* = 7.4 Hz, 3H), 1.67 (s, 16H), 1.56 – 1.49 (m, 2H), 1.48 – 1.37 (m, 6H), 1.26 (s, 9H).

^{13}C NMR (126 MHz, CD_3CN) δ 174.9, 174.3, 173.4, 154.9, 154.8, 144.1, 143.3, 142.4, 142.3, 129.5, 129.5, 126.0, 125.9, 125.6, 123.2, 123.1, 112.0, 111.8, 104.1, 76.1, 73.3, 70.9, 61.4, 50.2, 50.1, 45.1, 44.9, 39.8, 37.8, 36.6, 32.2, 32.0, 30.4, 30.2, 30.1, 29.8, 27.9, 27.8, 27.8, 27.6, 27.5, 27.0, 26.1. HRMS (ESI) m/z: $[M]^+$ calculated for $C_{46}H_{65}N_4O_4$, 737.4996 found 737.5000.

Tags with TEG spacers

General procedure A| Cy TEG amide couplings

Cy-carboxylic acid was dissolved in dry DCM (0.2 M) and cooled to 0°C. Amine **S6** (1 eq), DMAP (0.05 eq) and DIC (1.2 eq) were added and the mixture was stirred overnight at rt. The mixture was loaded directly on a silica column and purification by flash chromatography.

***t*-Bu-TEG-Cy3 (S9)**

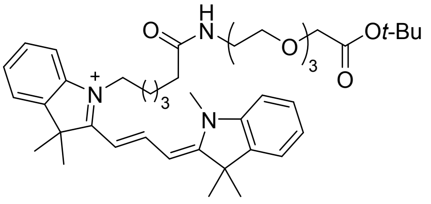

Reaction of **X-S2**[21] (132 mg, 0.50 mmol) with Cy3-carboxylic acid[22] (229 mg, 0.5 mmol) according to general procedure A followed by flash chromatography (DCM/MeOH, 1/0 -> 94.5/5.5 v/v) afforded the title compound as a red solid (0.24 g, 0.33 mmol 66%).

^1H NMR (300 MHz, $CDCl_3$) δ = 8.45 (t, *J*=13.5, 1H), 7.57 – 7.36 (m, 5H), 7.34 – 7.06 (m, 5H), 6.98 (d, *J*=13.4, 1H), 4.17 (t, *J*=7.7, 2H), 4.02 (d, *J*=1.4, 2H), 3.81 (d, *J*=1.5, 3H), 3.76 – 3.55 (m, 10H), 3.46 (dd, *J*=7.8, 3.8, 2H), 2.38 (t, *J*=7.1, 2H), 1.90 (q, *J*=9.3, 8.5, 2H), 1.75 (d, *J*=2.2, 14H), 1.71 – 1.58 (m, 2H), 1.47 (s, 9H).

^{13}C NMR (75 MHz, $CDCl_3$) δ 174.1, 173.5, 173.3, 150.4, 142.3, 141.5, 140.3, 140.1, 128.6, 128.6, 125.2, 122.0, 121.9, 110.7, 110.5, 104.2, 103.7, 70.3, 70.1, 70.1, 69.8, 69.3, 68.6, 48.8, 48.7, 46.1, 44.4, 38.6, 35.8, 31.9, 27.8, 27.8, 26.8, 26.0, 24.9. HRMS (ESI) m/z: $[M]^+$ calculated for $C_{42}H_{60}N_3O_6$ 702.4477, found 702.4473.

COOH-TEG-Cy3 (S10)

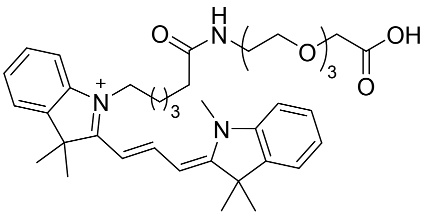

Tert-butyl ester **S9** (121 mg, 0.165 mmol) was dissolved in TFA/DCM (2.42 ml, 0.1 M, 17%) and stirred for 4 hours at rt. The mixture was diluted with toluene (20 ml) and evaporated (3x) to furnish the product as a red solid. (112 mg, 0.164 mmol, quant.)

^{1}H NMR (400 MHz, $CDCl_3$) δ = 8.41 (t, *J*=13.4, 1H), 7.83 (t, *J*=5.6, 1H), 7.48 – 7.34 (m, 4H), 7.34 – 7.23 (m, 3H), 7.16 (dd, *J*=8.0, 6.0, 2H), 6.49 (dd, *J*=19.3, 13.5, 2H), 4.20 (s, 2H), 4.06 (t, *J*=7.8, 2H), 3.80 – 3.72 (m, 2H), 3.73 – 3.55 (m, 11H), 3.52 – 3.43 (m, 2H), 2.42 (t, *J*=7.5, 2H), 1.89 – 1.64 (m, 16H), 1.60 – 1.49 (m, 2H).
^{13}C NMR (101 MHz, $CDCl_3$) δ 176.0, 174.6, 174.2, 172.4, 150.6, 142.6, 141.8, 140.6, 140.4, 129.2, 129.0, 125.8, 125.7, 122.3, 122.2, 111.2, 110.9, 103.6, 103.4, 71.1, 70.5, 70.4, 70.0, 69.4, 68.9, 49.4, 49.2, 46.3, 44.5, 39.8, 35.5, 31.5, 28.1, 28.1, 27.1, 26.3, 25.6. HRMS (ESI) m/z: $[M]^+$ calculated for $C_{38}H_{52}N_3O_6$ 646.38506 found 646.38514

_t_-Bu-TEG-Cy5 (S11)

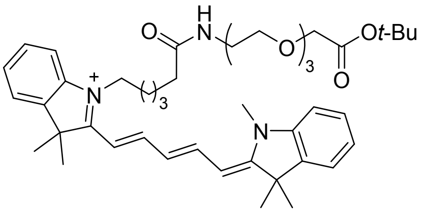

Reaction of **X-S2**[21] (66 mg, 0.25 mmol) with Cy5-carboxylic acid[22] (130 mg, 0.25 mmol) according to general procedure A followed by flash chromatography (DCM/MeOH, 98/2 -> 95/5, v/v) afforded the title compound as a blue solid. (139 mg, 0.183 mmol, 73%)
^{1}H NMR (400 MHz, $CDCl_3$) δ 8.21 (t, *J* = 13.0 Hz, 2H), 7.42 – 7.35 (m, 6H), 7.23 (dt, *J* = 10.6, 5.4 Hz, 2H), 7.14 (t, *J* = 6.8 Hz, 3H), 6.79 (t, *J* = 12.4 Hz, 1H), 6.29 (t, *J* = 14.6 Hz, 2H), 4.10 – 4.05 (m, 2H), 4.02 (s, 2H), 3.78 – 3.62 (m, 12H), 3.60 (t, *J* = 5.6 Hz, 2H), 3.51 – 3.40 (m, 2H), 2.34 (t, *J* = 7.1 Hz, 2H), 1.87 – 1.79 (m, 2H), 1.78 (s, 6H), 1.76 (s, 6H), 1.64 – 1.50 (m, 2H), 1.47 (s, 9H) ppm.
^{13}C NMR (101 MHz, $CDCl_3$) δ 173.2, 173.1, 169.6, 154.0, 153.7, 142.6, 141.8, 141.2, 140.8, 128.5, 128.5, 126.2, 125.1, 124.9, 122.2, 122.1, 110.6, 110.3, 103.7, 103.5, 81.5, 77.5, 70.5, 70.4, 70.4, 70.0, 69.6, 68.9, 49.4, 49.2, 44.2, 38.9, 35.9, 31.8, 28.0, 28.0, 27.9, 27.0, 26.4, 25.1 ppm. HRMS (ESI) m/z: $[M]^+$ calculated for $C_{44}H_{62}N_3O_6$ 728.4633 found 728.4628

COOH-TEG-Cy5 (S12)

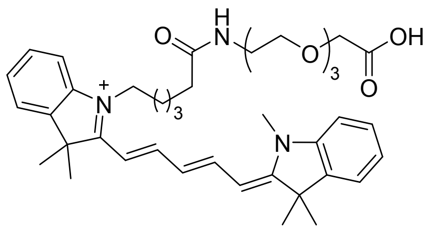

Ester **S11** (139 mg, 0.18 mmol) was dissolved in TFA/DCM (1.8 ml, 0.1 M, 50%) and stirred for 30 minutes at rt. The mixture was diluted with toluene (20 ml) and evaporated (3x) to furnish the title product as a blue solid. (128 mg, 0.18 mmol, quant.)
^{1}H NMR (400 MHz, $CDCl_3$) δ 9.46 (br s, 2H, COOH), 7.93 (td, *J* = 12.9, 6.4 Hz, 2H), 7.56 (m, 1H), 7.42 – 7.31 (m, 4H), 7.31 – 7.19 (m, 3H), 7.12 (dd, *J* = 16.3, 7.9 Hz, 2H), 6.72 (t, *J* = 12.4 Hz, 1H), 6.30 (d, *J* = 13.6 Hz, 1H), 6.21 (d, *J* = 13.5 Hz, 1H), 4.22 (s, 2H), 4.04 (t, *J* = 7.3 Hz, 2H), 3.79 – 3.72 (m, 2H), 3.71 – 3.56 (m, 11H), 3.53 – 3.30 (m, 2H), 2.36 (t, *J* = 7.3 Hz, 2H), 1.87 – 1.59 (m, 16H), 1.59 – 1.44 (m, 2H) ppm.
^{13}C NMR (101 MHz, $CDCl_3$) δ 174.5, 173.3, 172.9, 172.3, 153.7, 153.0, 142.8, 141.9, 141.2, 140.8, 128.9, 128.7, 126.1, 125.5, 125.1, 122.3, 122.2, 111.0, 110.4, 104.1, 103.5, 70.9, 70.5, 70.0, 69.8, 69.0, 49.5, 49.2, 44.4, 39.4, 35.9, 31.5, 31.3, 28.1, 27.1, 26.4, 25.4 ppm. HRMS (ESI) m/z: $[M]^+$ calculated for $C_{40}H_{54}N_3O_6$ 672.4007 found 672.4003

GG probes

General procedure B| Amide coupling reporter tag to warhead PFP method

The appropriate carboxylic acid (25 μmol) was dissolved in DMF (0.5 ml), 2,3,4,5,6-pentafluorophenol (23 mg, 0.13 μmol), Et_3N (10 μl, 0.13 mmol) and DIC (3.9 μl,25 μmol) were added and the mixture was stirred for 90 minutes. Part of the stock solution (1.2 eq acid compared to amine) was added to the amine and stirred overnight. LC-MS indicated full conversion and the product was purified on semi-preparative HPLC eluting with a linear gradient of solution A (MeCN) in solution B (50 mM AcOH in H_2O). The fractions were concentrated under reduced pressure, co-evaporated with water, diluted with water and lyophilized to yield the product.

GG Cy3 probe (50, ABP-Cel-Cy3)

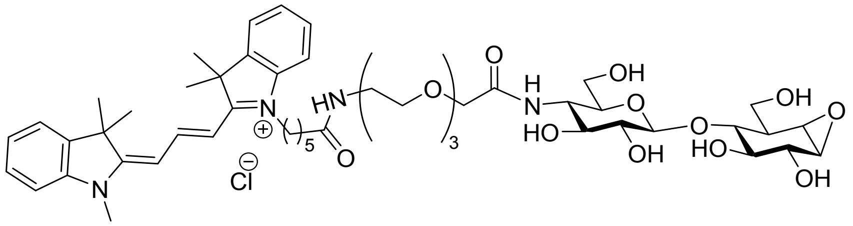

Amine **X-12**[21] (4.7 mg, 14 μmol) was reacted with stock solution TEG Cy3 **S10** (0.34 ml) according to general procedure B. Providing the product as a red solid contaminated with a small amount of an unknown byproduct. (9.7 mg, 9.7 μmol, 69%)

^{1}H NMR (600 MHz, D_2O) δ = 8.48 (t, *J*=13.4, 1H), 7.59 – 7.53 (m, 2H), 7.50 – 7.43 (m, 2H), 7.37 – 7.30 (m, 4H), 6.36 – 6.26 (m, 2H), 4.46 (d, *J*=8.0, 1H), 4.13 – 4.05 (m, 5H), 3.95 – 3.88 (m, 1H), 3.87 – 3.80 (m, 2H), 3.73 – 3.65 (m, 6H), 3.66 – 3.54 (m, 10H), 3.54 – 3.47 (m, 4H), 3.42 – 3.37 (m, 1H), 3.31 – 3.26 (m, 2H), 3.23 (d, *J*=3.8, 1H), 2.33 – 2.27 (m, 1H), 2.28 – 2.22 (m, 2H), 1.90 – 1.82 (m, 2H), 1.73 – 1.70 (m, 12H), 1.69 – 1.62 (m, 2H), 1.41 – 1.32 (m, 2H).

^{13}C NMR (151 MHz, D_2O) δ 177.2, 176.0, 175.5, 173.6, 151.3, 143.3, 142.7, 141.6, 141.5, 129.3, 126.0, 123.0, 122.9, 112.0, 111.7, 103.6, 102.8, 102.7, 78.5, 75.4, 74.4, 73.7, 71.5, 70.8, 70.2, 70.1, 70.0, 69.4, 61.3, 60.5, 57.2, 55.9, 51.7, 49.8, 49.7, 44.3, 43.3, 31.5, 27.9, 27.7, 27.2, 26.0, 25.6. HRMS (ESI) m/z: $[M]^+$ calculated for $C_{51}H_{73}N_4O_{14}$ 965.5118 found 965.5116

XG probes

Phenyl 4-deoxy-4-azido-1-thio-β-D-glucopyranoside (S13)

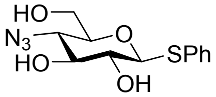

Benzoyl protected building block **X-9**[21] (1.98 g, 3.25 mmol) was dissolved in MeOH (32 ml) and DCM (7.5 ml). NaOMe (0.12 ml, 5.4 M, 0.65 mmol) was added and the mixture was stirred for 20 hours. The reaction was neutralized with AcOH and dry loaded in silica. Silica with the adsorbed material was loaded on a silica column and eluted (pentane/EtOAc, 7/3 → 6/4, v/v) to provide the product as a white solid. (790 mg, 2.63 mmol, 81%)

^{1}H NMR (400 MHz, MeOD) δ 7.56 – 7.52 (m, 2H), 7.33 – 7.22 (m, 3H), 4.59 (d, *J* = 9.8 Hz, 1H, H1), 3.80 (dd, *J* = 12.3, 2.1 Hz, 1H, H6a), 3.69 (dd, *J* = 12.3, 4.5 Hz, 1H, H6b), 3.53 (dd, *J* = 9.6, 8.6 Hz, 1H, H3), 3.41 (t, *J* = 9.8 Hz, 1H, H4), 3.29 – 3.20 (m, 2H, H2/H5).

^{13}C NMR (101 MHz, MeOD) δ 135.0, 132.8, 129.9, 128.4, 89.4 (C1), 80.1 (C5), 78.9 (C3), 73.8 (C2), 63.1 (C4), 62.6 (C6).

Phenyl 6-*O*-tert-butyldimethylsilyl-4-deoxy-4-azido-1-thio-β-D-glucopyranoside (S14)

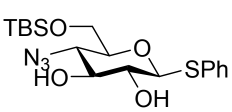

Triol **S13** (780 mg, 2.62 mmol) was dissolved in DMF (13 ml, 0.2 M). Imidazole (267 mg, 3.93 mmol) and TBSCl (475 mg, 3.15 mmol) were added and the mixture was stirred overnight. The reaction was diluted with water and extracted with EtOAc (3x) the combine organic layers were washed with water (3x) and brine, dried over $MgSO_4$ and concentrated under reduced pressure yielding a viscous oil (1.17 g) that was used and analyzed without further purification.

^{1}H NMR (400 MHz, $CDCl_3$) δ 7.62 – 7.57 (m, 2H, SPh), 7.35 – 7.30 (m, 3H, SPh), 4.50 (d, *J* = 9.7 Hz, 1H, H1), 3.96 (dd, *J* = 11.6, 1.7 Hz, 1H, H6a), 3.88 (dd, *J* = 11.6, 3.9 Hz, 1H, H6b), 3.68 – 3.53 (m, 2H, H3/H4), 3.40 (dd, *J* = 9.7, 8.5 Hz, 1H, H2), 3.29 – 3.24 (m, 1H, H5), 0.98 (s, 9H, *t*-Bu), 0.16 (s, 3H, CH_3Si), 0.15 (s, 3H, CH_3Si).

^{13}C NMR (101 MHz, $CDCl_3$) δ = 132.9, 131.6, 129.0, 128.2, 87.4 (C1), 79.4 (C5), 76.7 (C3), 72.0 (C2), 62.7 (C6), 61.3 (C4), 25.9 (*t*-Bu), 18.4 (*t*-Bu), -5.2 (Me), -5.4 (Me). HRMS (ESI) m/z: $[M+NH_4]^+$ calculated for $C_{18}H_{33}N_4O_4SSi$ 429.19863, found 429.19861.

Phenyl 2,3-*O*-di-benzoyl-4-deoxy-4-azido-1-thio-β-D-glucopyranoside (62)

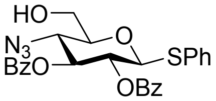

Crude diol **S14** (2.62 mmol) was dissolved in DCM (26 ml, 0.1 M). Pyridine (1.06 ml, 13.1 mmol) and BzCl (0.76 ml, 6.55 mmol) were added and the mixture was stirred overnight. The reaction was quenched with water, poured over $NaHCO_3$ (aq. sat.) and extracted with DCM. The organic layer was washed with brine, dried over $MgSO_4$ and concentrated under reduced pressure.
The crude product was dissolved in THF (21 ml, 0.1M), TBAF (5.24 ml, 1 M in THF, 5.24 mmol) was added and the mixture was stirred for 22 hours. The reaction was poured over $NaHCO_3$ (aq. sat.). The mixture was extracted with EtOAc (2x). The combined organic layers were washed with brine (2x), dried over $MgSO_4$ and the volatiles were removed under reduced pressure. The pure product was obtained after chromatography (pentane/Et_2O, 8/2 → 65/35, v/v) as a colorless oil. (1.05 g, 2.07 mmol, 79% over 3 steps)
^{1}H NMR (400 MHz, $CDCl_3$) δ 7.98 – 7.89 (m, 4H), 7.56 – 7.48 (m, 2H), 7.48 – 7.43 (m, 2H), 7.42 – 7.34 (m, 4H), 7.34 – 7.28 (m, 3H), 5.66 (t, *J* = 9.7 Hz, 1H, H3), 5.36 (t, *J* = 9.7 Hz, 1H, H2), 4.97 (d, *J* = 10.0 Hz, 1H, H1), 4.03 (dd, *J* = 12.4, 2.3 Hz, 1H, H6a), 3.95 (t, *J* = 10.1 Hz, 1H, H4), 3.86 (dd, *J* = 12.4, 4.1 Hz, 1H, H6b), 3.53 (ddd, *J* = 10.3, 4.1, 2.4 Hz, 1H, H5).
^{13}C NMR (101 MHz, $CDCl_3$) δ = 165.8, 165.3, 133.6, 133.5, 133.0, 131.9, 130.0, 130.0, 129.2, 129.1, 128.8, 128.6, 128.6, 86.3 (C1), 78.8 (C5), 75.0 (C3), 70.6 (C2), 62.0 (C6), 59.9 (C4). HRMS (ESI) m/z: $[M+Na]^+$ calculated for $C_{26}H_{23}N_3O_6SNa$ 528.11998, found 528.12004.

Phenyl 6-*O*-(2,3,4-tri-*O*-benzyl-α-D-xylopyranosyl)-2,3-*O*-di-benzoyl-4-deoxy-4-azido-1-thio-β-D-glucopyranoside (63)

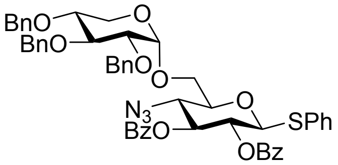

Acceptor **62** (100 mg, 0.197 mmol) was co-evaporated with toluene (3x). DCM (2 ml), DIPEA (86 µl, 0.493 mmol), $OPPh_3$ (274 mg, 0.985 mmol) and 3Å molecular sieves were added and the mixture was stirred for 1 hour at room temperature.
Acetate donor **56**[23] (182 mg, 0.394 mmol) was co-evaporated with toluene (3x). The dry residue was dissolved in DCM (1 ml) followed by the addition of TMSI (59 µl, 0.414 mmol) at room temperature. The mixture was stirred for 30 minutes and turned deep red. The mixture was co-evaporated with toluene (2x), dissolved in DCM (0.5 ml) and added to the reaction flask containing the acceptor. Upon addition the color faded immediately, and the reaction was stirred overnight. The reaction was quenched by the addition of water. The mixture was filtered over celite and diluted with EtOAc. The layers were separated and the organic layer was washed with , $NaHCO_3$ (aq. sat.) and brine, dried over $MgSO_4$ and concentrated under reduced pressure. The product was isolated after column chromatography (pentane/Et_2O, 8/2, v/v). (125 mg, 0.138 mmol, 70%)
^{1}H NMR (400 MHz, $CDCl_3$) δ 8.00 – 7.92 (m, 2H), 7.88 – 7.83 (m, 2H), 7.56 – 7.48 (m, 4H), 7.44 – 7.21 (m, 22H), 5.59 (t, *J* = 9.7 Hz, 1H, H3), 5.32 (t, *J* = 9.7 Hz, 1H, H2), 4.96 – 4.87 (m, 3H, H1', CH_2Bn), 4.85 (d, *J* = 10.0 Hz, 1H, H1), 4.75 (m, 2H, CH_2Bn), 4.66 – 4.61 (m, 2H, CH_2Bn), 3.99 – 3.85 (m, 4H, H4/H6ab/H3'), 3.71 – 3.55 (m, 4H, H5ab'/H4'/H5), 3.49 (dd, *J* = 9.6, 3.5 Hz, 1H, H2').
^{13}C NMR (101 MHz, $CDCl_3$) δ = 165.8, 165.3, 139.1, 138.5, 138.3, 133.9, 133.6, 133.5, 131.7, 130.1, 130.0, 129.3, 129.2, 128.9, 128.7, 128.6, 128.5, 128.5, 128.2, 128.1, 128.0, 128.0, 127.9, 127.9, 127.8, 127.7, 97.5 (C1'), 86.5 (C1), 81.2 (C3'), 80.1 (C2'), 78.8, 78.2(C4'/C5), 75.9 (CH_2Bn), 75.0 (C3), 73.6 (CH_2Bn), 73.4 (CH_2Bn), 70.7 (C2), 66.0 (C6), 60.4 (C5' and C4 weak). HRMS (ESI) m/z: $[M+Na]^+$ calculated for $C_{52}H_{49}N_3O_{10}SNa$ 930.3063, found 930.3023

4-*O*-(6-*O*-(2,3,4-tri-*O*-benzyl-α-D-xylopyranosyl)-2,3-*O*-di-benzoyl-4-deoxy-4-azido-β-D-glucopyranosyl)-2,3,6-tri-*O*-benzyl-cyclophellitol (20)

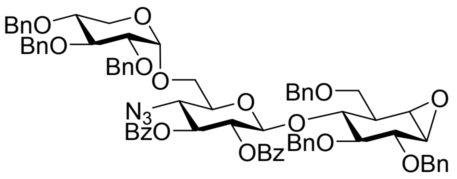

Donor **63** (108 mg, 0.119 mmol), Ph_2SO (33 mg, 0.162 mmol) and TTBP (134 mg, 0.54 mmol) were co-evaporated with toluene (2x). DCM (1.2 ml) and 3 Å molecular sieves were added and the mixture was stirred for 30 minutes at room

temperature. The mixture was cooled to -70°C and Tf_2O (0.25 ml, 0.59 M in DCM, 0.148 mmol) was added and the mixture was allowed to warm to -40°C within 30 minutes. The mixture was cooled again to -70°C and acceptor **X-19**[19] (48 mg, 0.108 mmol) dissolved in DCM (0.5 ml) was added.
The mixture was slowly warm to room temperature overnight, diluted with DCM and poured over brine. The layers were separated and the organic layer was dried over $MgSO_4$. The product was obtained after chromatography (Pentane/EtOAc, 9/1 -> 8/2, v/v). (85 mg, 0.068 mmol, 63%)
^{1}H NMR (500 MHz, $CDCl_3$) δ 7.93 – 7.89 (m, 2H), 7.88 – 7.84 (m, 2H), 7.52 – 7.46 (m, 2H), 7.44 – 7.17 (m, 36H), 5.43 (t, *J* = 9.9 Hz, 1H, H3'), 5.30 (dd, *J* = 9.8, 7.9 Hz, 1H, H2'), 4.97 (d, *J* = 12.3 Hz, 1H, CH_2Bn), 4.91 (d, *J* = 3.5 Hz, 1H, H1''), 4.89 – 4.81 (m, 3H, CH_2Bn), 4.80 (d, *J* = 8.0 Hz, 1H, H1'), 4.73 (d, *J* = 11.8 Hz, 1H, CH_2Bn)), 4.65 – 4.57 (m, 4H, CH_2Bn)), 4.55 (d, *J* = 11.4 Hz, 1H, CH_2Bn), 4.32 (d, *J* = 11.8 Hz, 1H, CH_2Bn), 4.15 (d, *J* = 11.8 Hz, 1H, CH_2Bn), 3.94 (t, *J* = 10.1 Hz, 1H, H4'), 3.92 – 3.87 (m, 1H, H3''), 3.86 (d, *J* = 7.4 Hz, 1H, H2), 3.69 (t, *J* = 9.8 Hz, 1H, H4), 3.62 (m, 2H, H6a/H6a'), 3.59 – 3.47 (m, 5H, H5ab''/H6b'/H4''/H3), 3.43 (t, *J* = 8.5 Hz, 1H, H6b), 3.37 – 3.31 (m, 2H, H2''/epoxide), 3.17 (ddd, *J* = 10.2, 3.9, 1.6 Hz, 1H, H5'), 3.09 (d, *J* = 3.7 Hz, 1H, epoxide), 2.18 (dddd, *J* = 9.8, 8.2, 3.5, 1.4 Hz, 1H, H5).
^{13}C NMR (126 MHz, $CDCl_3$) δ = 165.8, 165.2, 139.4, 139.0, 138.6, 138.5, 138.2, 137.7, 133.5, 133.4, 130.0, 129.9, 129.3, 129.0, 128.6, 128.6, 128.5, 128.5, 128.5, 128.4, 128.4, 128.4, 128.3, 128.1, 127.9, 127.9, 127.8, 127.8, 127.7, 127.7, 127.6, 127.6, 127.6, 127.3, 126.7, 101.6 (C1'), 97.7 (C1''), 83.3 (C3), 81.3 (C3''), 79.9 (C2''), 79.6 (C2), 78.1 (C4''), 75.9 (C4), 75.7 (CH_2Bn), 74.9 (C5'), 74.2 (CH_2Bn), 74.0 (C3'), 73.4 (CH_2Bn), 73.2 (CH_2Bn), 73.1 (C2'), 73.1 (CH_2Bn), 73.0 (CH_2Bn), 68.6 (C6), 65.5 (C6'), 60.4 (C4'/C5'' weak), 55.5 (epoxide), 53.3 (epoxide), 42.0 (C5). HRMS (ESI) m/z: $[M+Na]^+$ calculated for $C_{74}H_{73}N_3O_{15}Na$ 1266.4934, found 1266.4942

4-*O*-(6-*O*-(2,3,4-tri-*O*-benzyl-α-D-xylopyranosyl)-4-deoxy-4-azido-β-D-glucopyranosyl)-2,3,6-tri-*O*-benzyl-cyclophellitol (64)

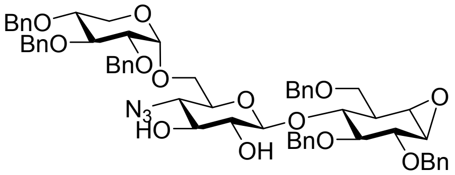

20 (57 mg, 68 µmol) was dissolved in MeOH/DCM (1 ml, 1/1, v/v). NaOMe (10 µl, 5.4M, 42 µmol) was added and the mixture was stirred overnight. NH_4Cl was added and the solvent was evaporated under reduced pressure. Column chromatography (DCM/EtOAc, 1/0 -> 9/1, v/v) provided the product. (57 mg, 55 µmol, 81%)
^{1}H NMR (500 MHz, $CDCl_3$) δ = 7.36 – 7.21 (m, 30H), 4.86 (d, *J*=11.7, 1H, CH_2Bn), 4.84 – 4.78 (m, 3H, CH_2Bn/H1''), 4.76 (d, *J*=10.9, 1H, CH_2Bn), 4.70 (d, *J*=11.8, 1H, CH_2Bn), 4.68 – 4.55 (m, 7H, CH_2Bn), 4.41 (d, *J*=7.8, 1H, H1'), 3.90 – 3.77 (m, 5H, H4/H3''/H6ab/), 3.61 – 3.46 (m, 6H), 3.44 – 3.37 (m, 2H), 3.35 (dd, *J*=9.6, 3.5, 1H, H2''), 3.30 – 3.28 (m, 1H, epoxide), 3.15 – 3.09 (m, 2H, H2', epoxide), 3.00 (ddd, *J*=10.1, 4.2, 1.5, 1H), 2.33 – 2.26 (m, 1H, H5).
^{13}C NMR (126 MHz, $CDCl_3$) δ 139.0, 138.8, 138.7, 138.6, 137.5, 128.6, 128.6, 128.5, 128.5, 128.5, 128.5, 128.4, 128.1, 128.1, 128.0, 128.0, 127.8, 127.7, 127.7, 127.6, 127.5, 127.1, 102.9 (H1'), 97.7 (H1''), 83.5, 81.3, 79.8 (H2''), 79.7, 78.1, 75.7, 75.5, 75.0, 74.7, 74.3, 74.2, 73.4, 73.0, 72.7, 69.3, 66.2, 61.2, 60.3, 56.1 (epoxide), 53.1 (epoxide), 41.9 (C5). HRMS (ESI) m/z: $[M+NH_4]^+$ calculated for $C_{60}H_{69}N_4O_{13}$ 1053.4856, found 1053.4848

4-*O*-(6-*O*-(α-D-xylopyranosyl)-4-deoxy-4-amino-β-D-glucopyranosyl)-cyclophellitol (65)

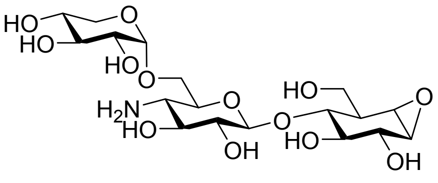

Ammonia (8 ml) was condensed at -50°C. Sodium (77 mg, 3.4 mmol) was added. Starting material **64** (58 mg, 0.056 mmol) was dissolved in THF (2 ml) and *t*-BuOH (0.37 ml, 3.9 mmol) and the mixture was added dropwise at – 65°C. After 40 minutes the reaction was quenched with NH_4Cl (195 mg, 3.6 mmol). The mixture was warmed to room temperature and the ammonia was evaporated.
The crude product was purified by size exclusion chromatography over HW-40 eluting with 1% AcOH in H_2O yielding the product as a white solid. (20 mg, 0.043 mmol, 76%)

^{1}H NMR (400 MHz, MeOD) δ 4.84 (d, *J* = 3.7 Hz, 1Hn H1xyl), 4.38 (d, *J* = 7.9 Hz, 1H, H1gluc), 4.11 (dd, *J* = 10.9, 4.1 Hz, 1H, H6a), 3.94 – 3.62 (m, 5H), 3.61 – 3.35 (m, 9H), 3.30 – 3.26 (m, 1H, H2gluc), 3.06 (d, *J* = 3.6 Hz, 1H, epoxide), 2.89 (t, *J* = 9.8 Hz, 1H, H4gluc), 2.23 – 2.16 (m, 1H, H5).
^{13}C NMR (101 MHz, MeOD) δ 105.2 (C1xyl), 100.9 (C1gluc), 81.5, 76.8, 75.6, 75.3, 75.0, 73.7, 73.5, 72.9, 71.4, 69.3, 63.4, 62.0 (C6), 56.7 (epoxide), 56.5 (epoxide), 56.0 (C4gluc), 44.9 (C5). HRMS (ESI) m/z: $[M+H]^+$ calculated for $C_{18}H_{32}NO_{13}$ 470.1868, found 470.1871

XG Cy5 probe (66, ABP-XyG-Cy5)

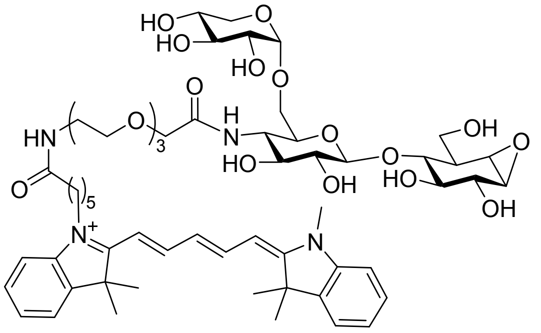

Amine **65** (4.4 mg, 9.4 μmol) was reacted with stock solution TEG Cy5 **S12** (0.24 ml) according to general procedure B. (2.64 mg, 2.2 μmol, 23%)
^{1}H NMR (600 MHz, MeOD) δ = 8.30 – 8.21 (m, 2H), 7.50 (dt, *J*=7.5, 1.5, 2H), 7.44 – 7.39 (m, 2H), 7.32 – 7.23 (m, 4H), 6.64 (t, *J*=12.4, 1H), 6.29 (dd, *J*=13.7, 4.9, 2H), 4.73 (d, *J*=3.6, 1H, H1xyl), 4.32 (d, *J*=8.0, 1H, H1gluc), 4.14 – 4.08 (m, 3H), 4.03 (d, *J*=1.7, 2H), 3.82 (dd, *J*=10.9, 7.2, 1H), 3.79 – 3.58 (m, 16H), 3.57 – 3.46 (m, 5H), 3.45 – 3.39 (m, 2H), 3.39 – 3.32 (m, 7H), 3.05 (d, *J*=3.7, 1H, epoxide), 2.24 (t, *J*=7.4, 2H), 2.22 – 2.17 (m, 1H, H5), 1.86 – 1.80 (m, 2H), 1.76 – 1.66 (m, 14H), 1.51 – 1.44 (m, 2H).
^{13}C NMR (151 MHz, MeOD) δ 176.0, 175.4, 174.7, 173.5, 144.3, 143.6, 142.7, 142.5, 129.8, 129.7, 126.6, 126.3, 123.4, 123.3, 112.1, 111.8, 105.6, 104.4 (C1gluc), 104.3, 100.0 (C1xyl), 82.7, 77.1, 75.7, 75.1, 74.9, 74.8, 73.9, 72.8, 71.8, 71.7, 71.4, 71.4, 71.2, 71.1, 71.1, 70.5, 68.0, 63.2, 62.4, 56.6 (epoxide), 56.5 (epoxide), 53.3, 50.6, 50.5, 49.8, 49.6, 44.9 (C5), 44.8, 40.3, 36.7, 31.5, 28.2, 28.0, 27.8, 27.4, 26.5. HRMS (ESI) m/z: $[M]^+$ calculated for $C_{58}H_{83}N_4O_{18}$ 1123.5697 found 1123.5690

XG azide probe (67, ABP-XyG-N3)

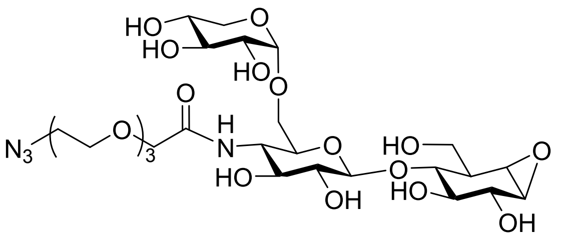

Amine **65** (4.4 mg, 9.4 μmol) was reacted with stock solution TEG azide **S4** (0.24 ml) according to general procedure B. (1.74 mg, 2.54 μmol, 27%)
^{1}H NMR (850 MHz, MeOD) δ 4.74 (d, *J* = 3.6 Hz, 1H, H1xyl), 4.32 (d, *J* = 8.0 Hz, 1H, H1gluc), 4.12 (dd, *J* = 10.9, 4.5 Hz, 1H), 4.03 (d, *J* = 1.9 Hz, 2H), 3.83 (dd, *J* = 10.9, 7.2 Hz, 1H), 3.81 – 3.77 (m, 1H), 3.77 – 3.74 (m, 2H), 3.74 – 3.67 (m, 11H), 3.59 (t, *J* = 9.4 Hz, 1H), 3.57 – 3.52 (m, 2H), 3.49 (t, *J* = 10.7 Hz, 1H), 3.45 – 3.40 (m, 4H), 3.40 – 3.37 (m, 1H), 3.36 – 3.32 (m, 2H), 3.06 (d, *J* = 3.6 Hz, 1H, epoxide), 2.23 – 2.19 (m, 1H, H5).
^{13}C NMR (214 MHz, MeOD) δ 173.5, 105.5 (C1gluc), 100.0 (C1xyl), 82.7, 77.1, 75.6, 75.1, 74.9, 74.8, 73.9, 72.8, 71.9, 71.7, 71.5, 71.4, 71.4, 71.2, 71.0, 68.0, 63.2, 62.4, 56.6 (epoxide), 56.5 (epoxide), 53.3, 51.8, 44.9 (C5). HRMS (ESI) m/z: $[M+H]^+$ calculated for $C_{26}H_{45}N_4O_{17}$ 685.2774 found 685.2768

XG biotin probe (68, ABP-XyG-Bio)

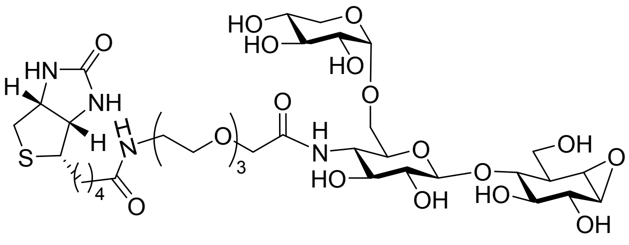

Amine **65** (4.4 mg, 9.4 μmol) was reacted with stock solution TEG biotin **S8** (0.24 ml) according to general procedure B. (2.14 mg, 2,44 μmol, 26%)
^{1}H NMR (850 MHz, MeOD) δ 4.74 (d, *J* = 3.6 Hz, 1H, H1xyl), 4.50 (ddd, *J* = 7.9, 5.0, 0.9 Hz, 1H), 4.34 – 4.29 (m, 2H, H1gluc), 4.12 (dd, *J* = 10.9, 4.5 Hz, 1H, H6a), 4.05 (d, *J* = 2.7 Hz, 2H), 3.84 (dd, *J* = 10.9, 7.1 Hz, 1H, H6b), 3.81 – 3.78 (m, 1H), 3.77 – 3.71 (m, 4H), 3.71 – 3.67 (m, 6H), 3.67 – 3.64 (m, 2H), 3.61 (t, *J* = 9.4 Hz, 1H), 3.58 – 3.52 (m, 4H), 3.50 (t, *J* = 10.7 Hz, 1H), 3.46 – 3.41 (m, 2H, epoxide),

3.41 – 3.37 (m, 3H), 3.36 – 3.34 (m, 2H), 3.34 – 3.32 (m, 2H), 3.24 – 3.20 (m, 1H), 3.06 (d, *J* = 3.6 Hz, 1H, epoxide), 2.93 (dd, *J* = 12.8, 5.0 Hz, 1H), 2.71 (d, *J* = 12.7 Hz, 1H), 2.27 – 2.19 (m, 3H, H5), 1.78 – 1.57 (m, 4H), 1.48 – 1.42 (m, 2H).

^{13}C NMR (214 MHz, MeOD) δ 176.2, 173.5, 105.5 (C1gluc), 100.0 (C1xyl), 82.7, 77.1, 75.7, 75.1, 74.9, 74.8, 73.9, 72.8, 71.8, 71.7, 71.4, 71.4, 71.2, 71.1, 70.6, 68.0, 63.4, 63.2, 62.4 (C6), 61.6, 57.0, 56.7, 56.5 (epoxide), 53.3 (epoxide), 44.9 (C5), 41.0, 40.3, 36.8, 29.8, 29.5, 26.9. HRMS (ESI) m/z: $[M+H]^+$ calculated for $C_{31}H_{53}N_4O_{15}S$ 753.3223 found 753.3219

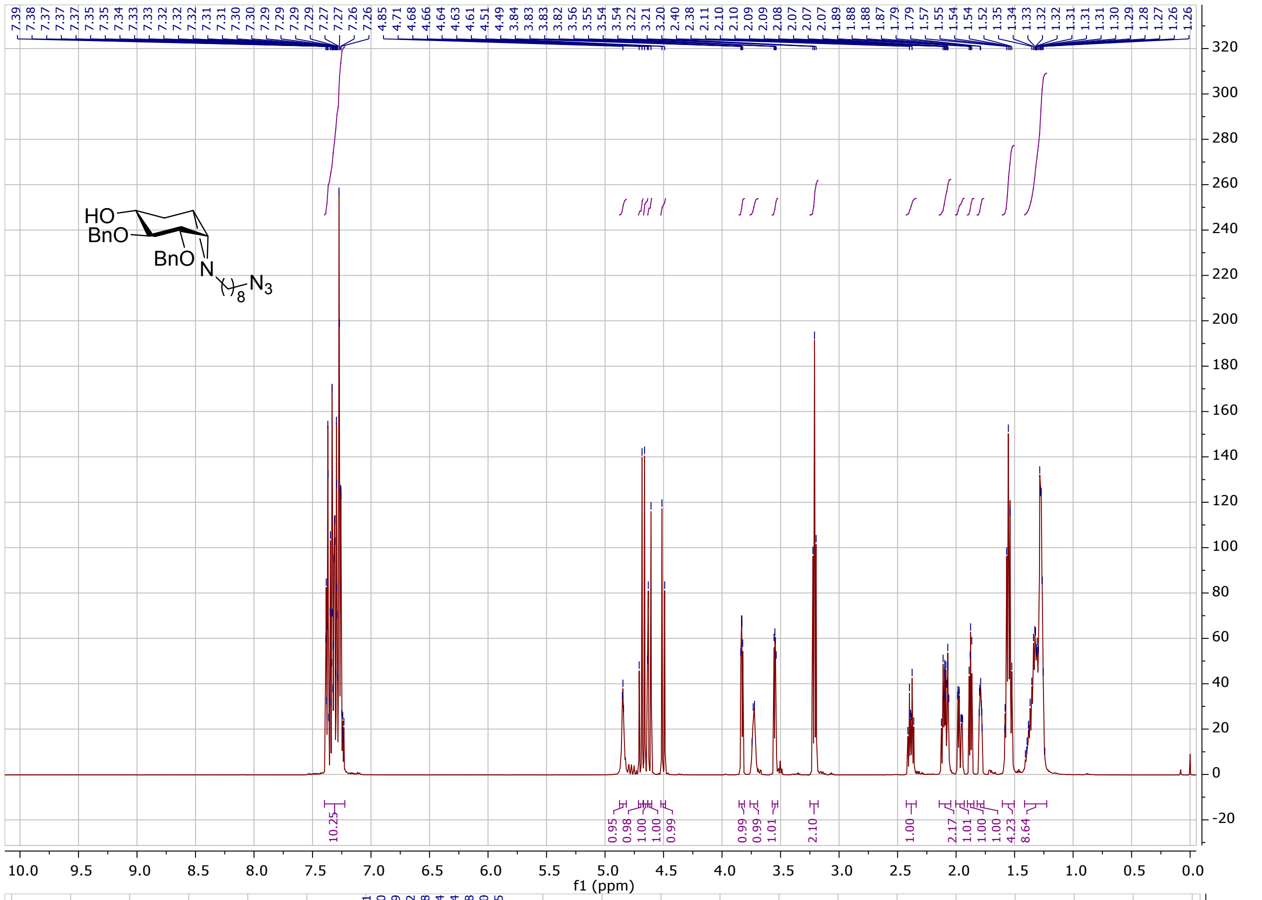
7.39 7.38 7.37 7.37 7.37 7.35 7.35 7.34 7.33 7.33 7.32 7.32 7.32 7.31 7.31 7.30 7.29 7.29 7.29 7.29 7.27 7.27 7.26 7.26 4.85 4.71 4.68 4.66 4.64 4.63 4.61 4.51 4.49 3.84 3.83 3.83 3.82 3.56 3.55 3.54 3.54 3.22 3.21 3.20 2.40 2.38 2.11 2.10 2.10 2.09 2.09 2.08 2.07 2.07 1.89 1.88 1.87 1.79 1.79 1.57 1.55 1.54 1.54 1.54 1.52 1.35 1.34 1.33 1.32 1.32 1.31 1.31 1.30 1.29 1.28 1.27 1.26 1.26
HO
BnO
BnO
N
8
N_3
10.25
0.95 0.98 1.00 1.00 0.99
0.99 0.99 1.01
2.10
1.00
2.17 1.01 1.00 1.00 4.23 8.64
f1 (ppm)
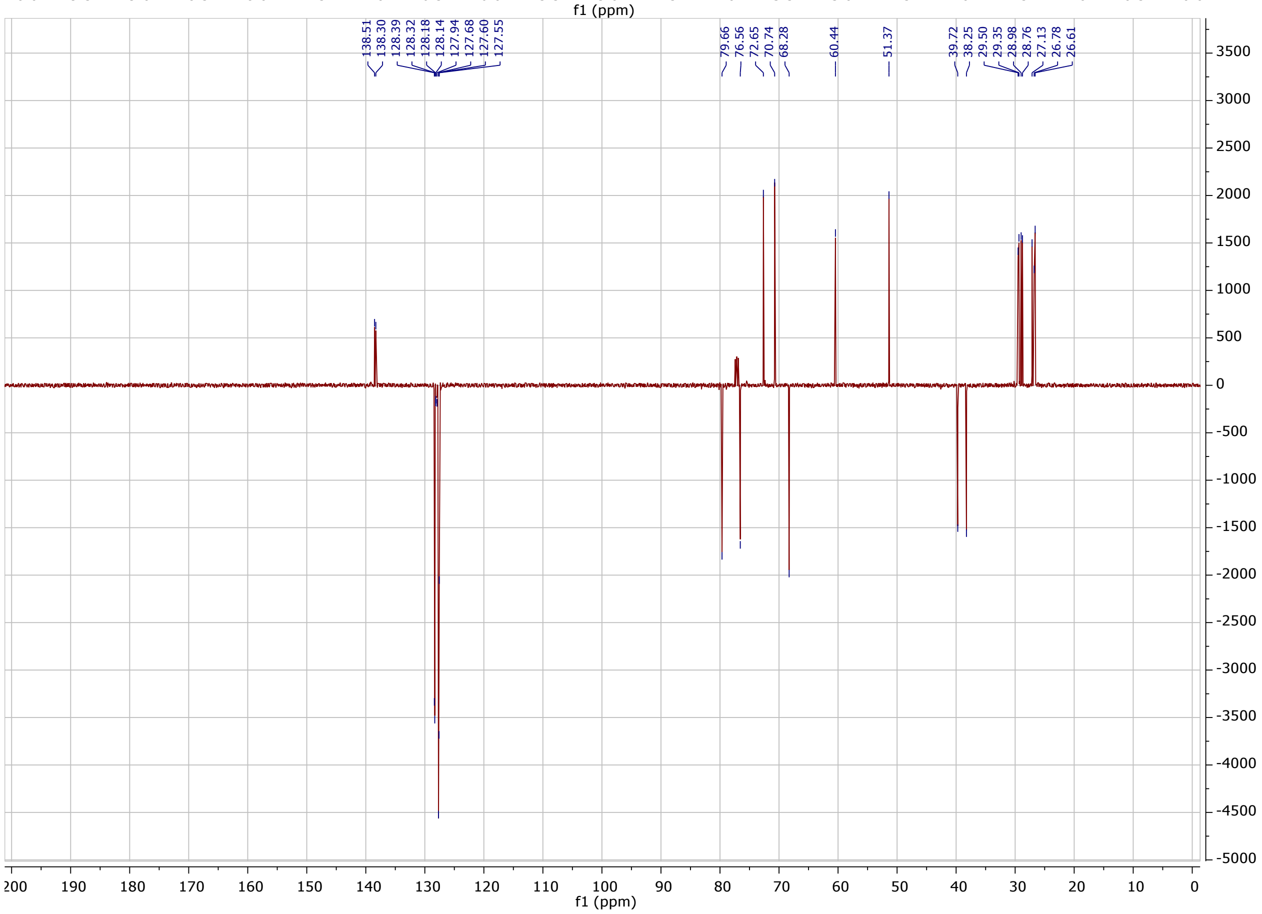
138.51 138.30 128.39 128.32 128.18 128.14 127.94 127.68 127.60 127.55
79.66 76.56 72.65 70.74 68.28
60.44
51.37
39.72 38.25 29.50 29.35 28.98 28.76 27.13 26.78 26.61
f1 (ppm)

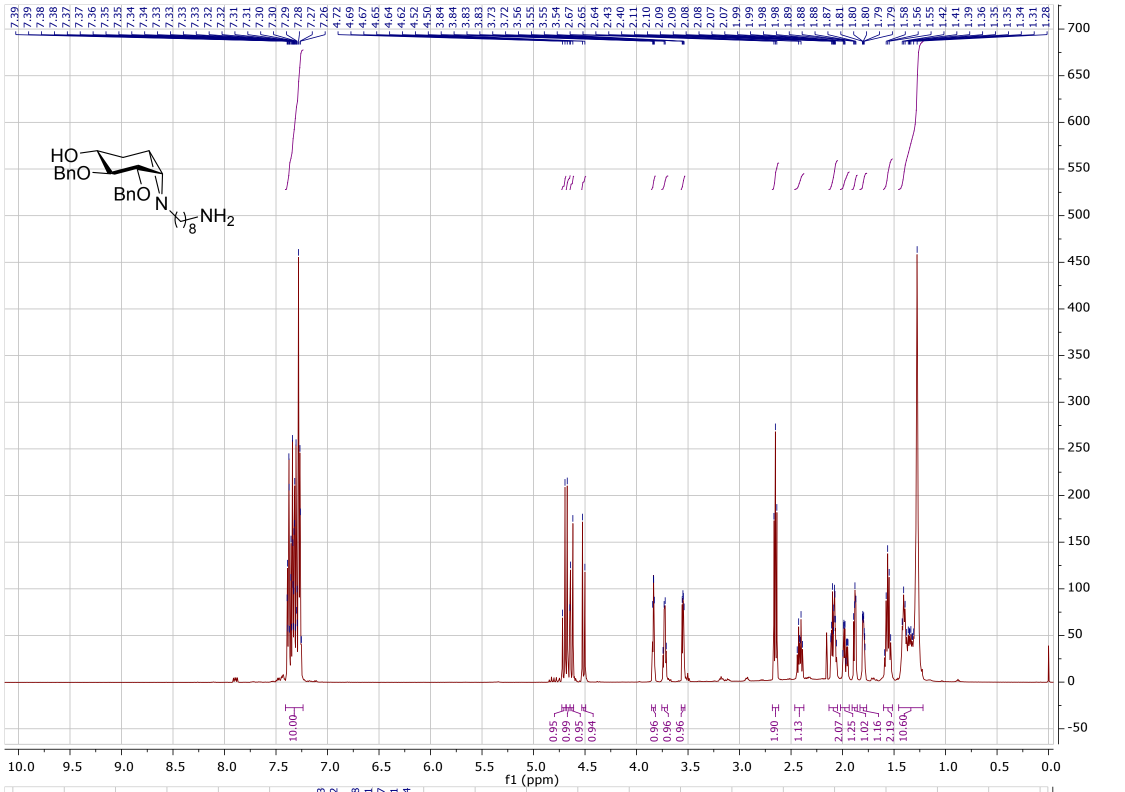
7.39 7.39 7.38 7.38 7.37 7.37 7.36 7.35 7.35 7.34 7.34 7.33 7.33 7.33 7.32 7.32 7.31 7.31 7.30 7.30 7.29 7.28 7.27 7.26 4.72 4.69 4.67 4.65 4.64 4.62 4.52 4.50 3.84 3.84 3.83 3.83 3.73 3.72 3.56 3.55 3.55 3.54 2.67 2.65 2.64 2.43 2.40 2.11 2.10 2.09 2.09 2.08 2.08 2.07 2.07 1.99 1.99 1.98 1.98 1.89 1.88 1.88 1.87 1.81 1.80 1.80 1.79 1.79 1.58 1.56 1.55 1.42 1.41 1.39 1.36 1.35 1.35 1.34 1.31 1.28
HO
BnO
BnO
N
8
NH2
10.00
0.95 0.99 0.95 0.94
0.96 0.96 0.96
1.90 1.13 2.07 1.25 1.02 1.16 2.19 10.60
f1 (ppm)

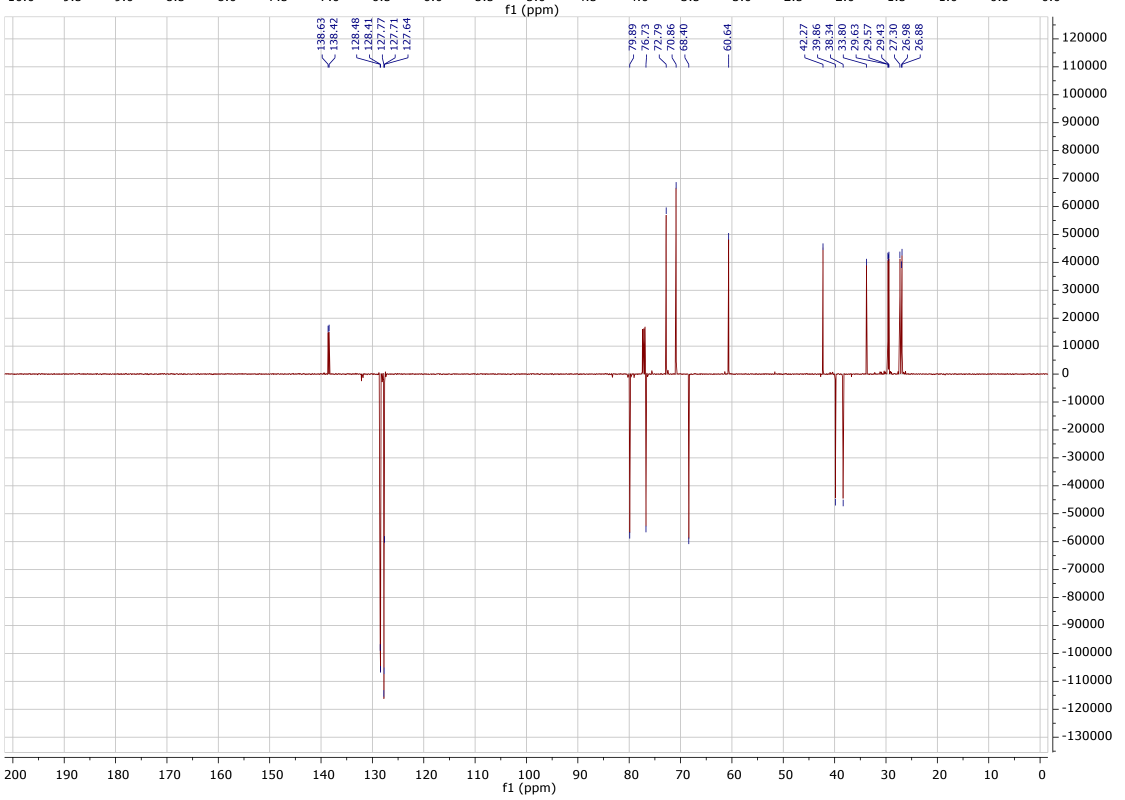
138.63 138.42 128.48 128.41 127.77 127.71 127.64
79.89 76.73 72.79 70.86 68.40
60.64
42.27 39.86 38.34 33.80 29.63 29.57 29.43 27.30 26.98 26.88
f1 (ppm)

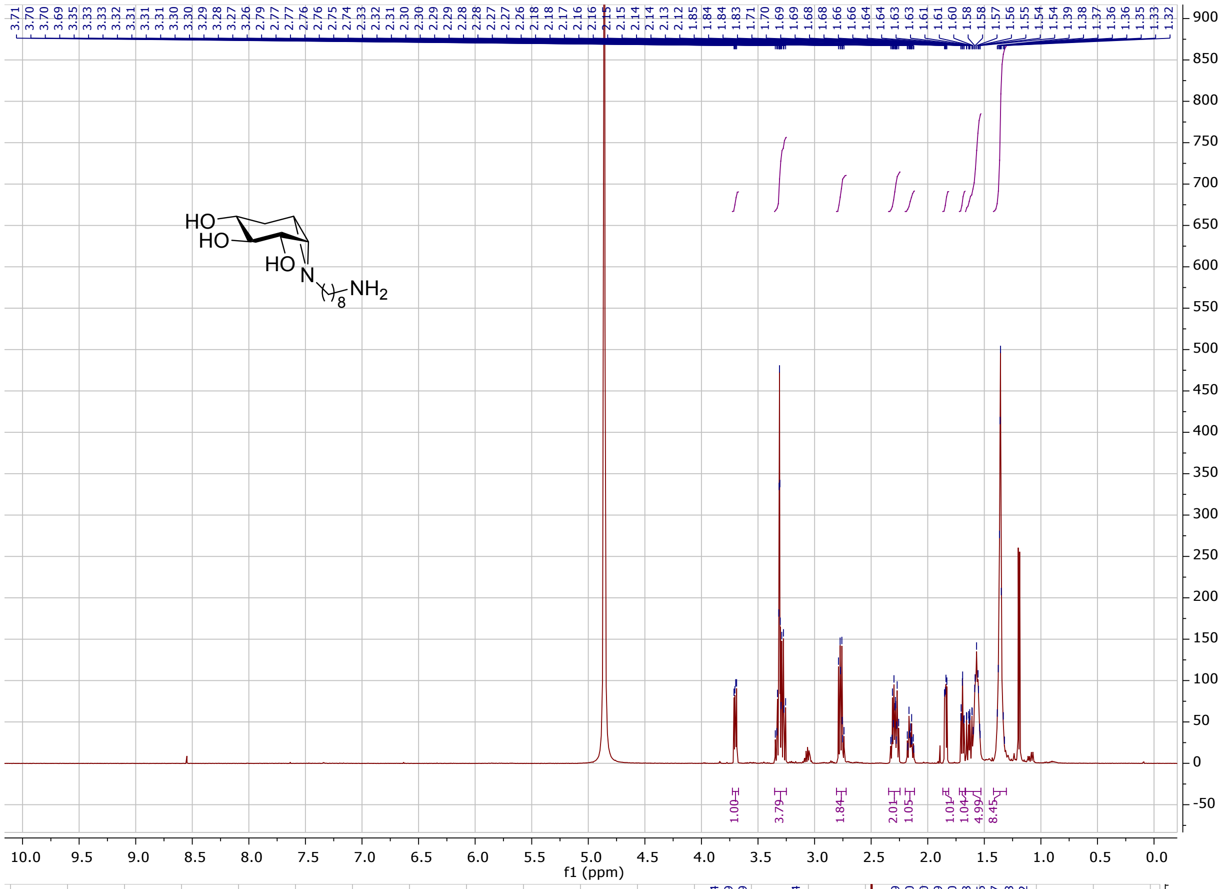

3.71 3.70 3.70 3.69 3.35 3.33 3.33 3.32 3.31 3.31 3.31 3.30 3.30 3.29 3.28 3.27 3.26 2.79 2.77 2.77 2.76 2.76 2.75 2.74 2.33 2.32 2.31 2.30 2.30 2.29 2.29 2.28 2.28 2.27 2.27 2.26 2.18 2.18 2.17 2.16 2.16 2.15 2.15 2.14 2.14 2.13 2.12 1.85 1.84 1.84 1.83 1.71 1.70 1.69 1.69 1.68 1.68 1.66 1.66 1.64 1.64 1.63 1.63 1.61 1.61 1.60 1.58 1.58 1.57 1.56 1.55 1.54 1.54 1.39 1.38 1.37 1.36 1.35 1.33 1.32
HO
HO
HO
N
8
NH2
1.00
3.79
1.84
2.01
1.05
1.01
1.04
4.99
8.45
f1 (ppm)

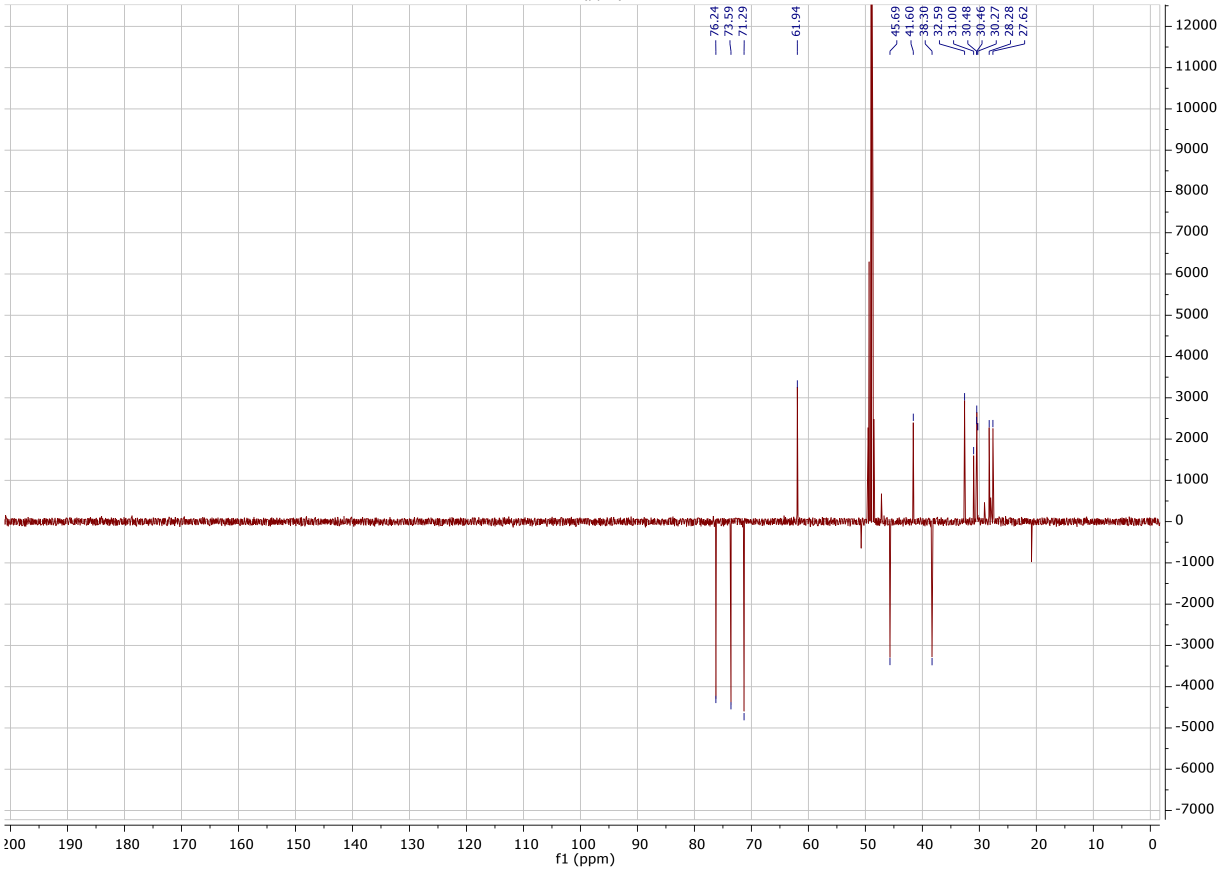

76.24 73.59 71.29
61.94
45.69 41.60 38.30 32.59 31.00 30.48 30.46 30.27 28.28 27.62
f1 (ppm)

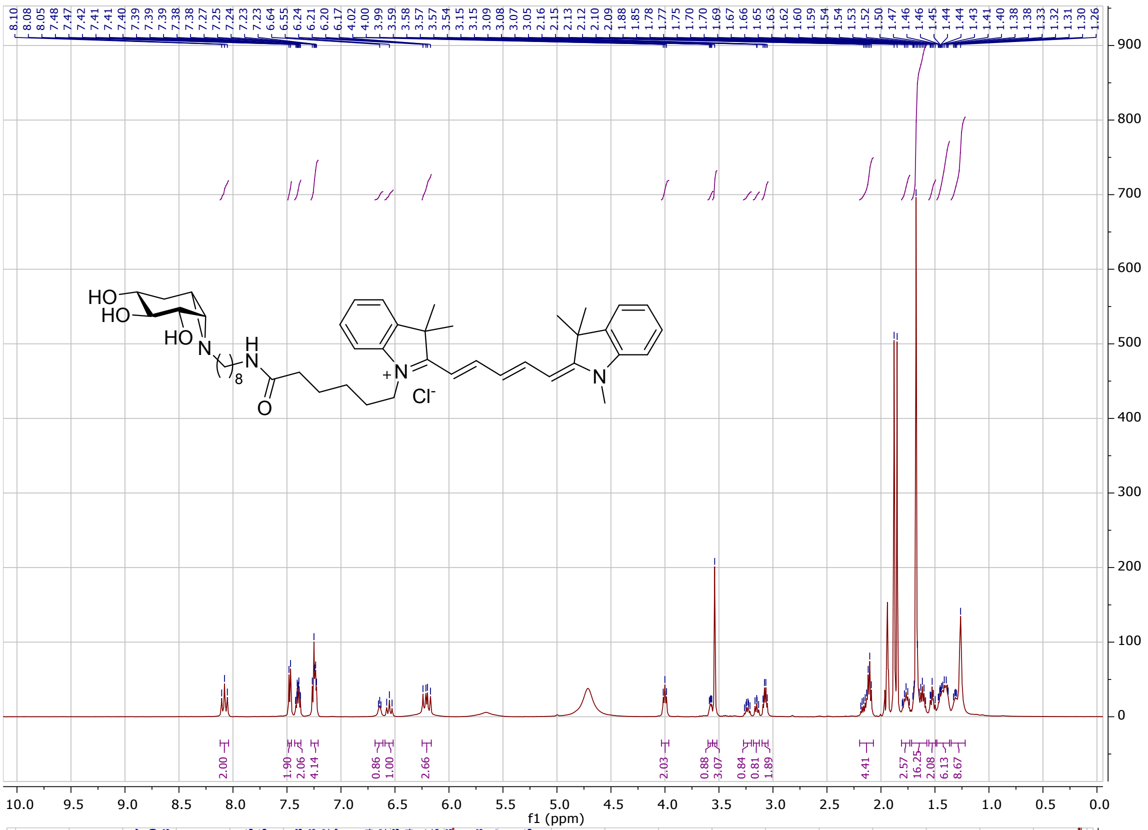
8.10
8.08
8.05
7.48
7.47
7.42
7.41
7.40
7.39
7.39
7.39
7.38
7.27
7.25
7.24
7.23
7.23
6.64
6.55
6.24
6.21
6.20
6.17
4.02
4.00
3.99
3.59
3.58
3.57
3.57
3.54
3.15
3.15
3.09
3.08
3.07
3.05
2.16
2.15
2.13
2.12
2.10
2.09
1.88
1.85
1.78
1.77
1.75
1.70
1.70
1.69
1.67
1.66
1.65
1.63
1.62
1.60
1.59
1.54
1.54
1.53
1.52
1.50
1.47
1.46
1.46
1.45
1.44
1.44
1.43
1.41
1.40
1.38
1.38
1.33
1.32
1.31
1.30
1.26
HO
HO
HO
N
H
N
8
O
+N
Cl-
N
2.00
1.90
2.06
4.14
0.86
1.00
2.66
2.03
0.88
3.07
0.84
0.81
1.89
4.41
2.57
16.25
2.08
6.13
8.67
f1 (ppm)

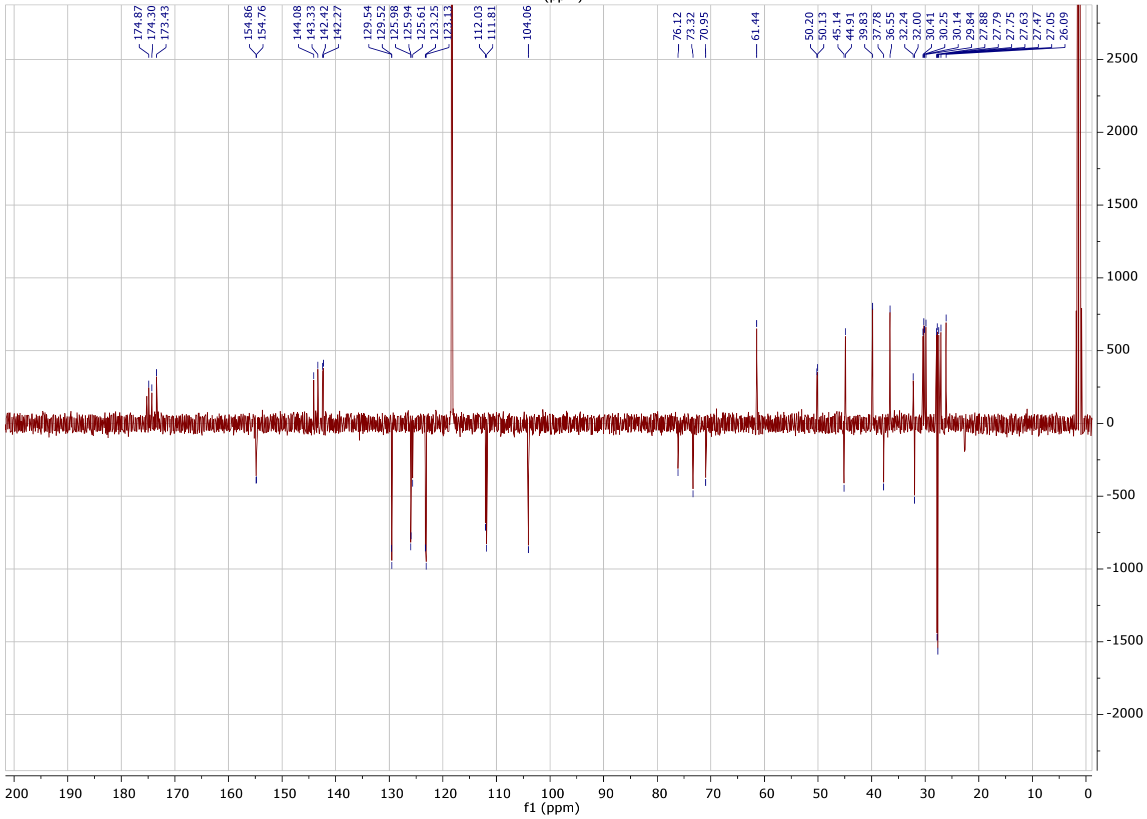
174.87
174.30
173.43
154.86
154.76
144.08
143.33
142.42
142.27
129.54
129.52
125.98
125.94
125.61
123.25
123.13
112.03
111.81
104.06
76.12
73.32
70.95
61.44
50.20
50.13
45.14
44.91
39.83
37.78
36.55
32.24
32.00
30.41
30.25
30.14
29.84
27.88
27.79
27.75
27.63
27.47
27.05
26.09
f1 (ppm)

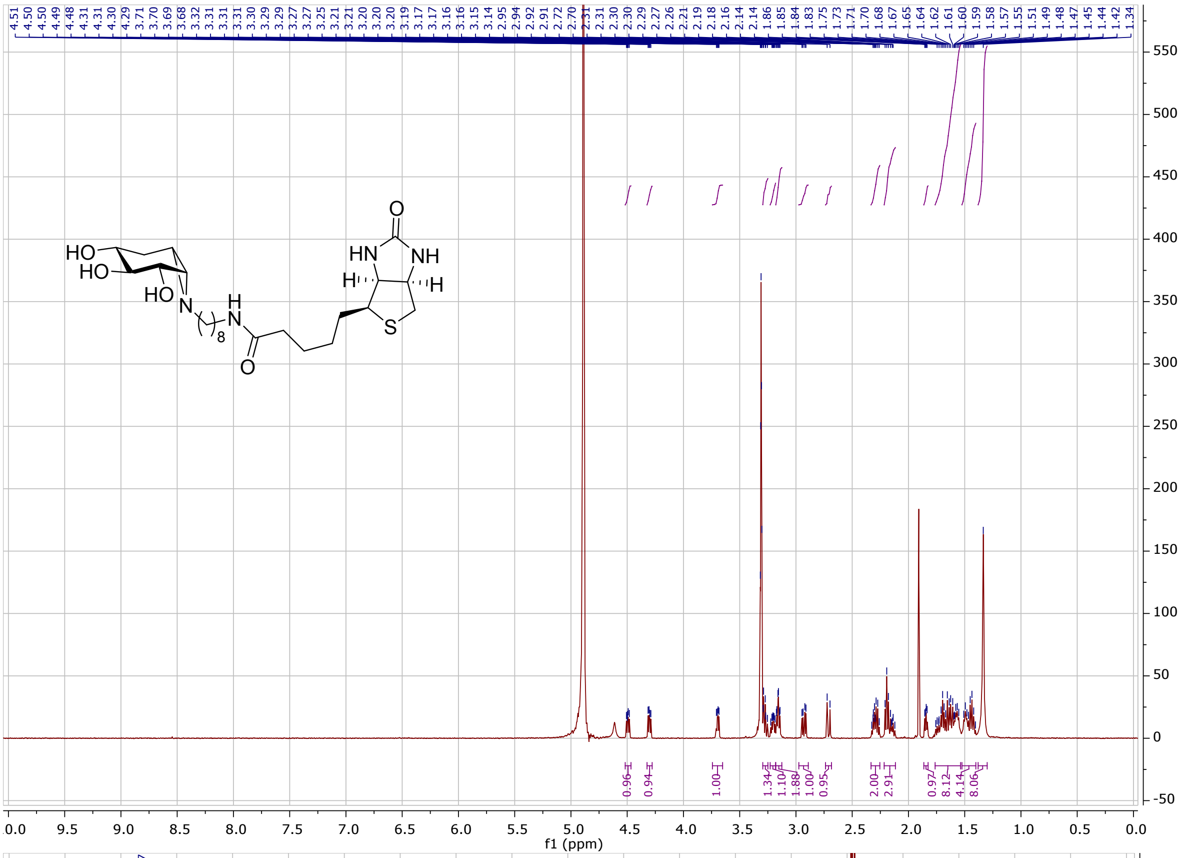

HO
HO
HO
N
H
N
8
O
HN
NH
O
H
H
S
0.96
0.94
1.00
1.34
1.10
1.88
1.00
0.95
2.00
2.91
0.97
8.12
4.14
8.06
f1 (ppm)

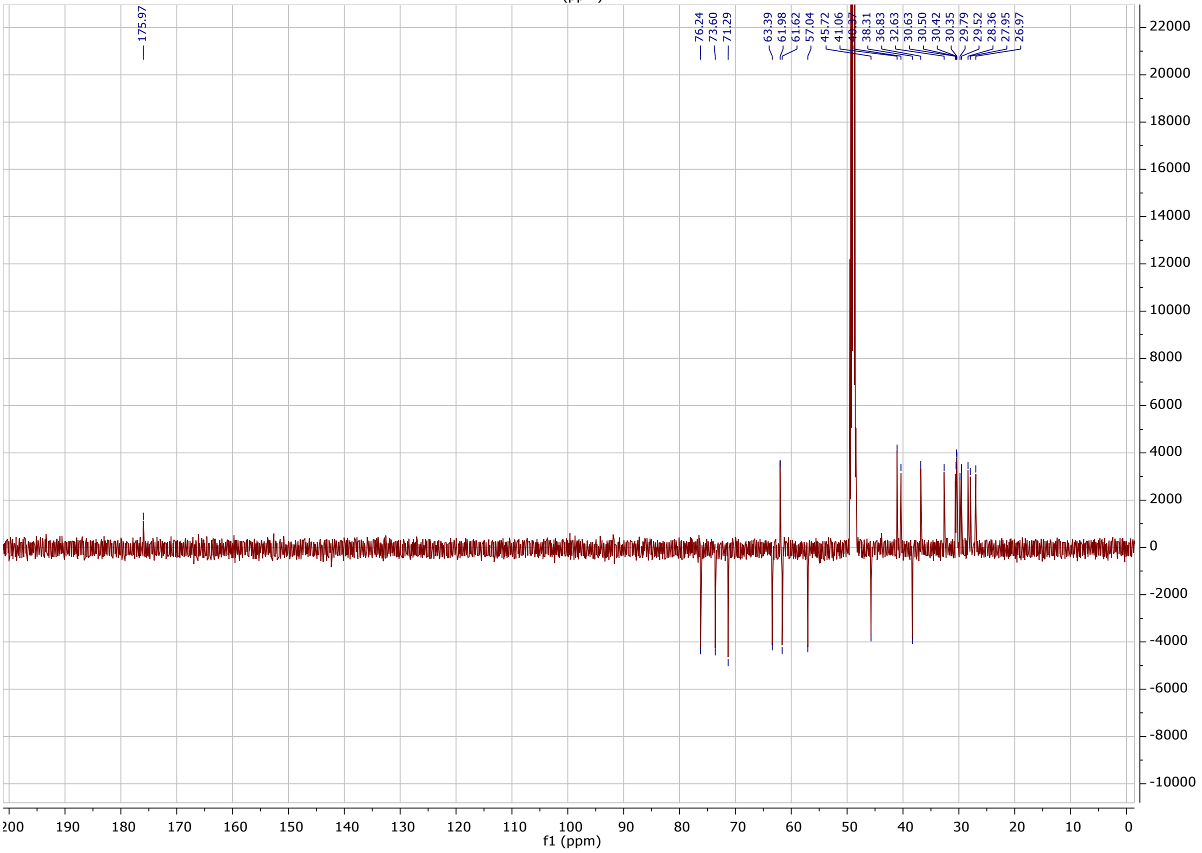

175.97
76.24
73.60
71.29
63.39
61.98
61.62
57.04
45.72
41.06
38.31
36.83
32.63
30.63
30.50
30.42
30.35
29.79
29.52
28.36
27.95
26.97
f1 (ppm)

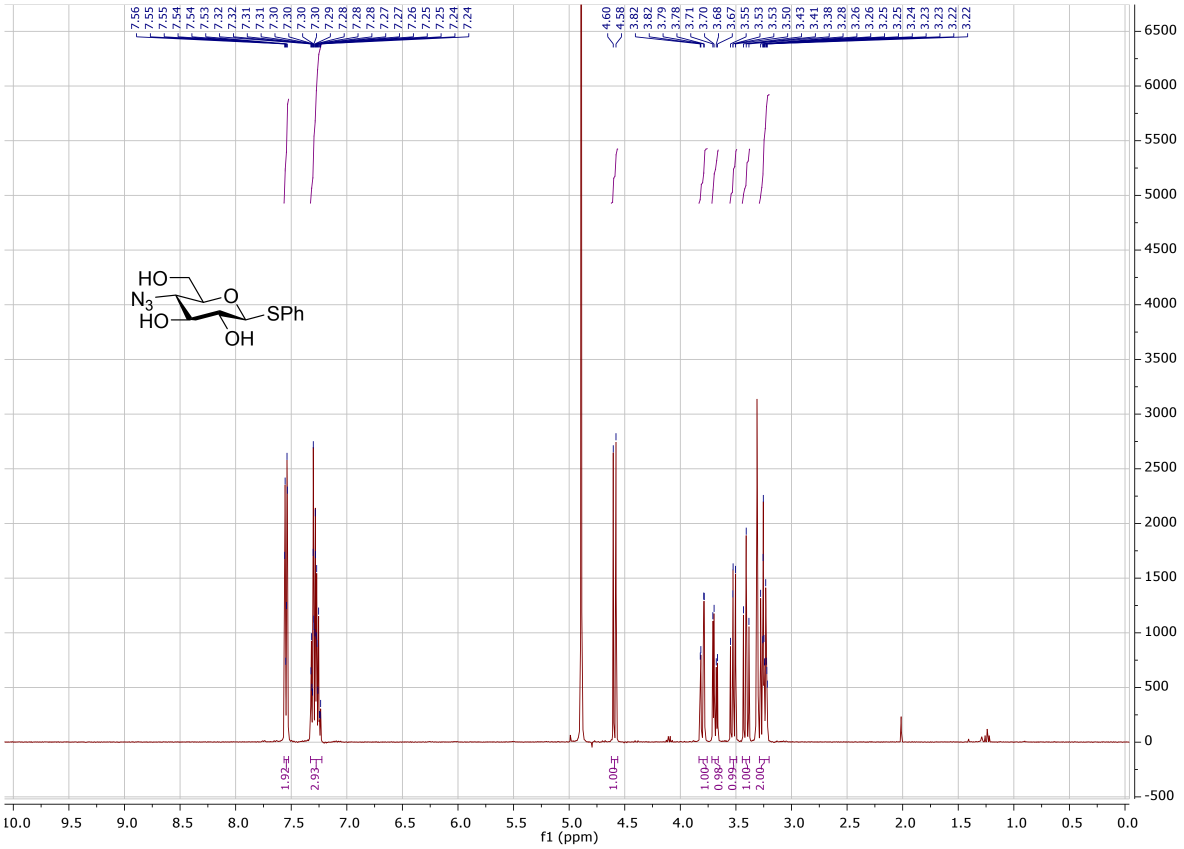

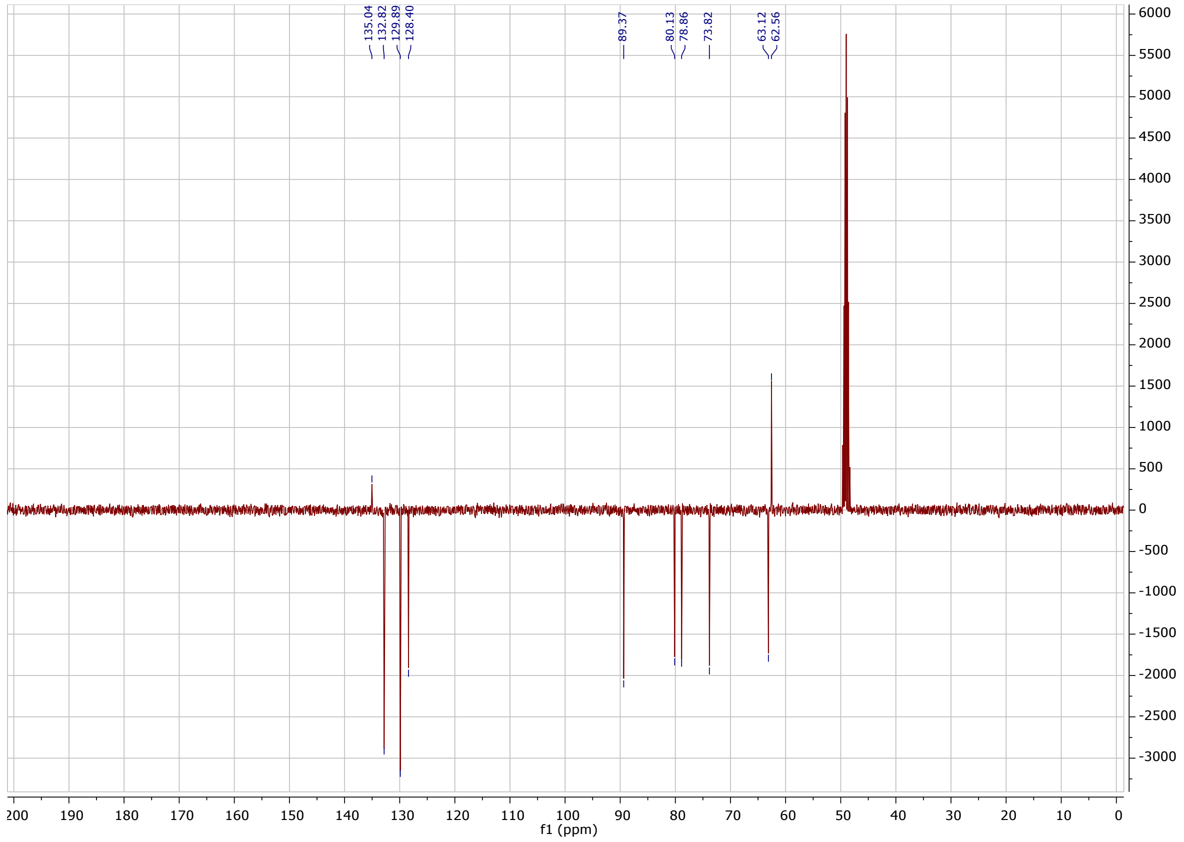

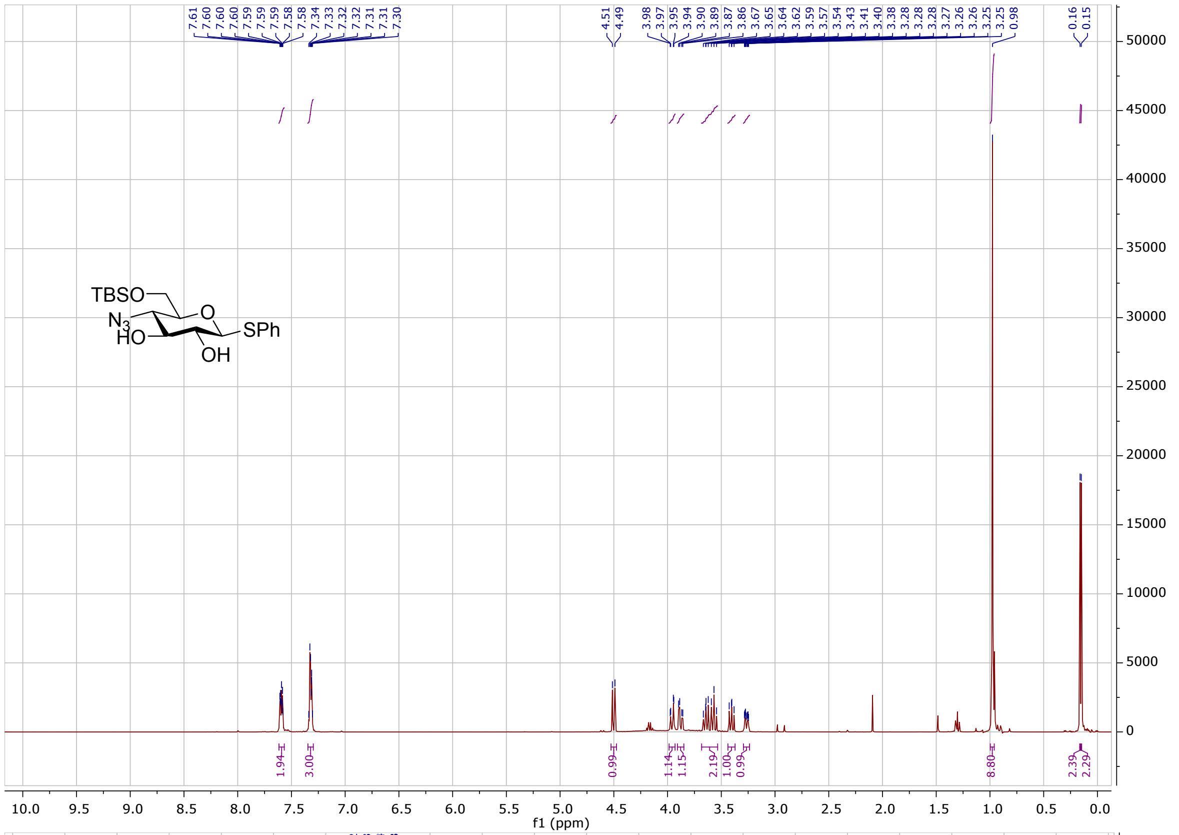
7.61 7.60 7.60 7.60 7.59 7.59 7.59 7.58 7.58 7.34 7.33 7.32 7.32 7.31 7.31 7.30
4.51 4.49
3.98 3.97 3.95 3.94 3.90 3.89 3.87 3.86 3.67 3.65 3.64 3.62 3.59 3.57 3.54 3.43 3.41 3.40 3.38 3.28 3.28 3.27 3.26 3.26 3.25 3.25 0.98
0.16 0.15
TBSO
N3
HO
O
SPh
OH
1.94
3.00
0.99
1.14
1.15
2.19
1.00
0.99
8.80
2.39
2.29
f1 (ppm)

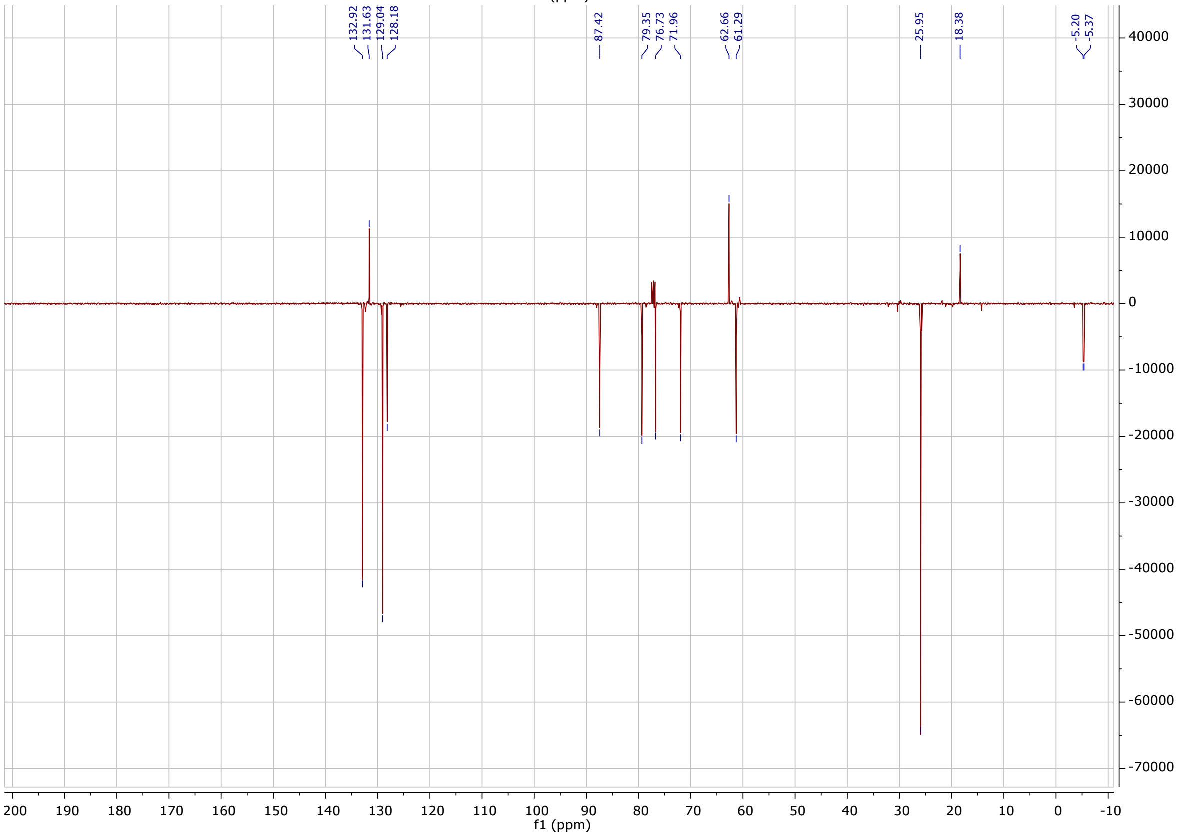
132.92 131.63 129.04 128.18
87.42
79.35 76.73 71.96
62.66 61.29
25.95
18.38
-5.20 -5.37
f1 (ppm)

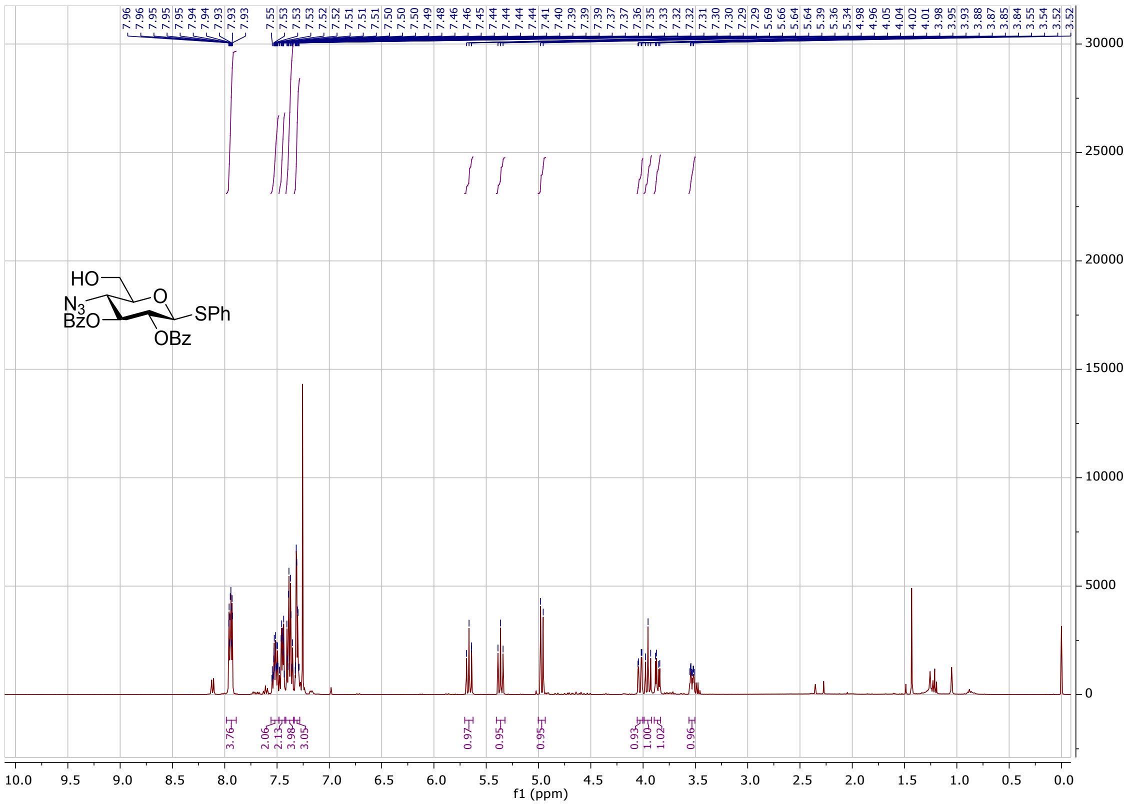

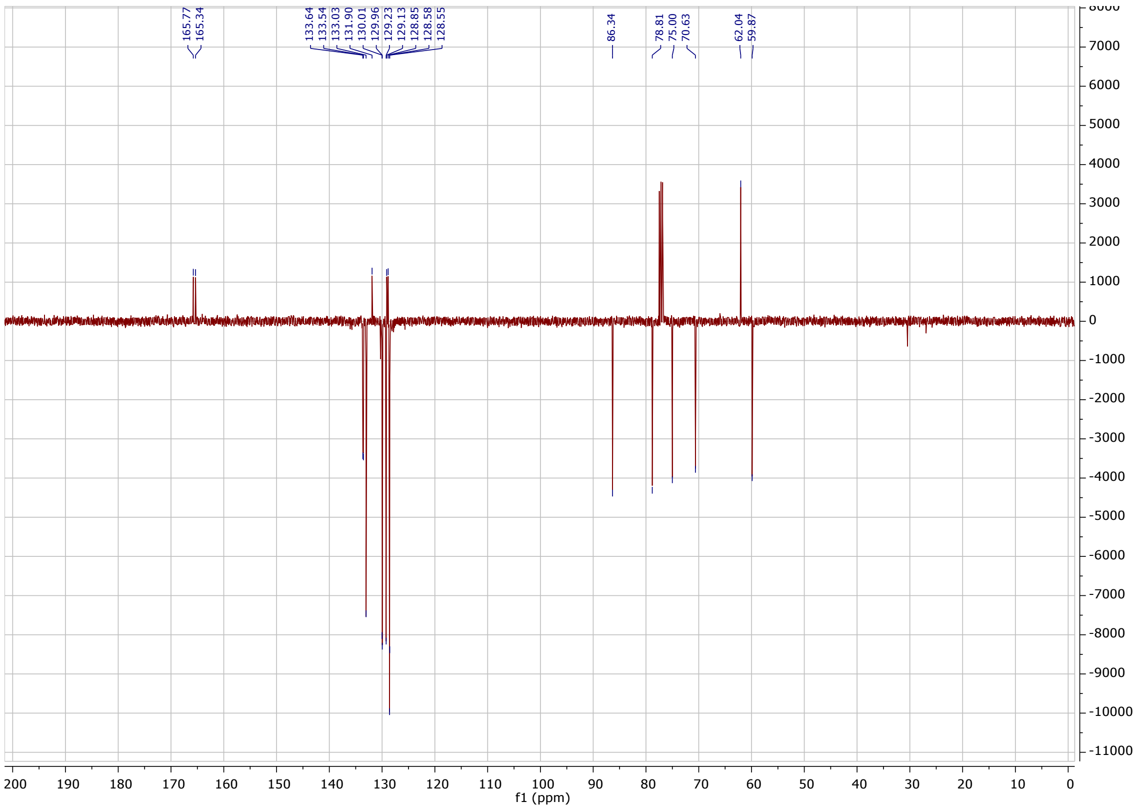

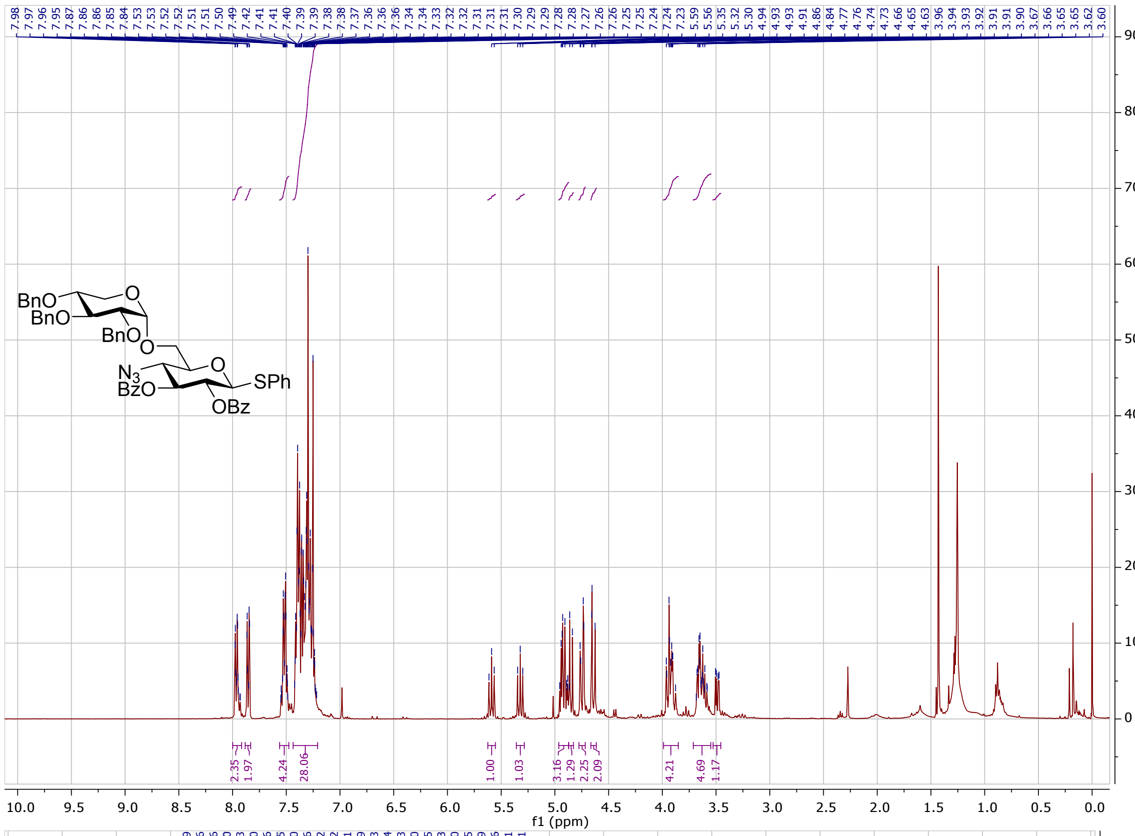

BnO
BnO
BnO
O
O
N3
BzO
O
SPh
OBz
f1 (ppm)

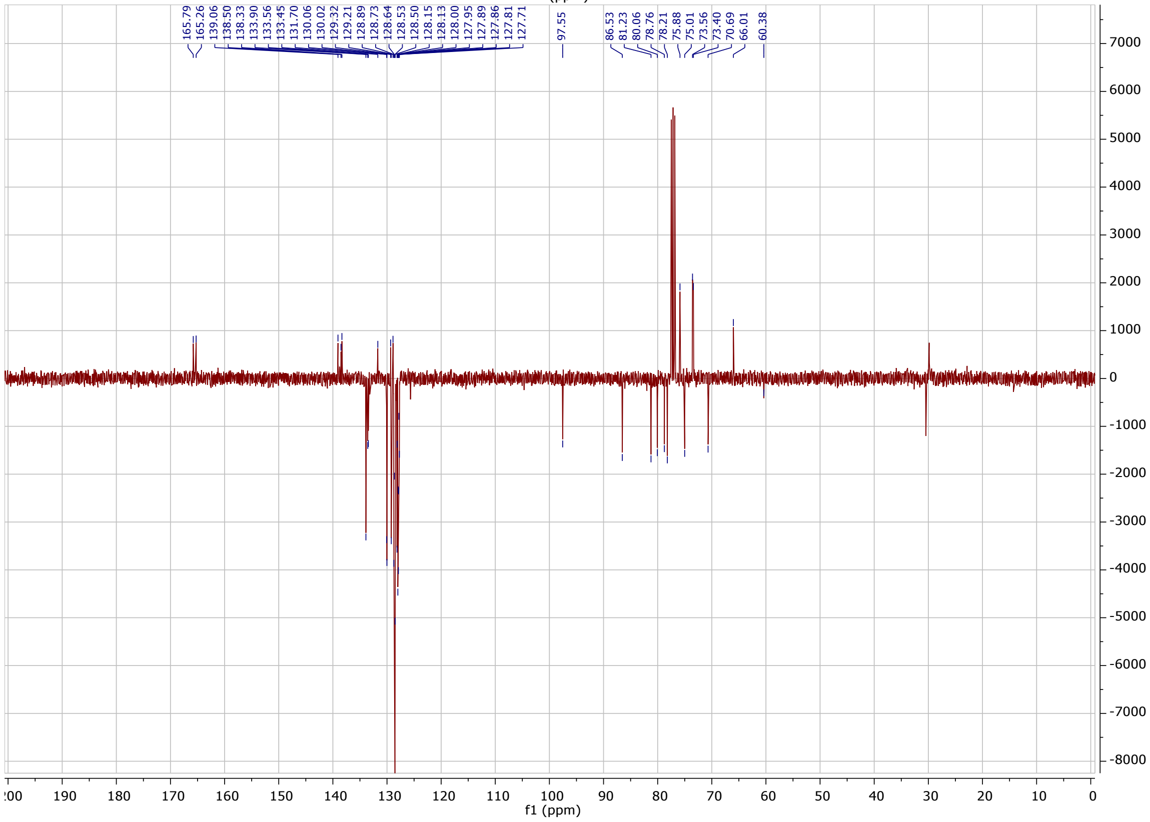

165.79
165.26
139.06
138.50
138.33
133.90
133.56
133.45
131.70
130.06
130.02
129.32
129.21
128.89
128.73
128.64
128.53
128.50
128.15
128.13
128.00
127.95
127.89
127.86
127.81
127.71
97.55
86.53
81.23
80.06
78.76
78.21
75.88
75.01
73.56
73.40
70.69
66.01
60.38
f1 (ppm)

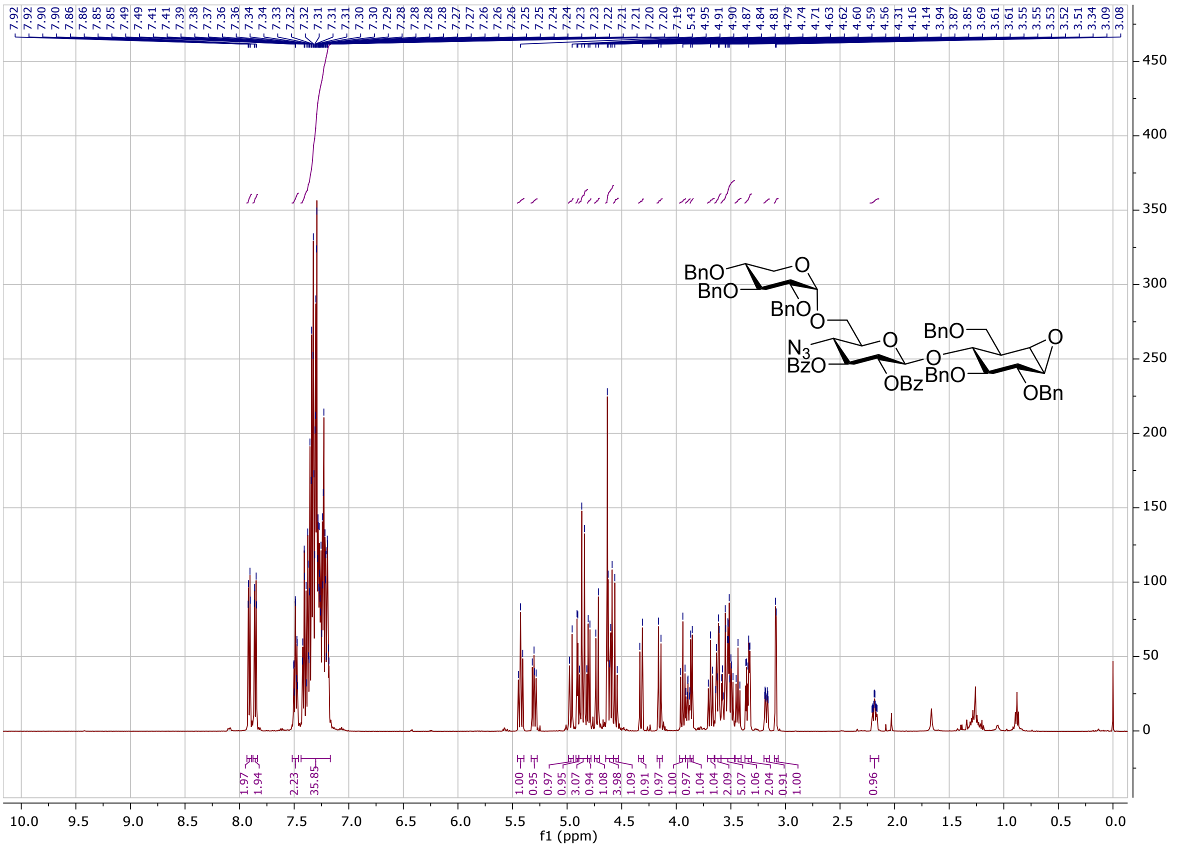
7.92 7.92 7.90 7.90 7.86 7.86 7.85 7.85 7.49 7.49 7.41 7.41 7.39 7.38 7.37 7.36 7.36 7.34 7.34 7.33 7.32 7.32 7.31 7.31 7.31 7.30 7.30 7.29 7.28 7.28 7.28 7.28 7.27 7.27 7.26 7.26 7.26 7.25 7.25 7.24 7.24 7.23 7.23 7.23 7.22 7.21 7.21 7.20 7.20 7.19 5.43 4.95 4.91 4.90 4.87 4.84 4.81 4.79 4.74 4.71 4.63 4.62 4.60 4.59 4.56 4.31 4.16 4.14 3.94 3.87 3.85 3.69 3.61 3.61 3.55 3.55 3.53 3.52 3.51 3.34 3.09 3.08
BnO
BnO
BnO
O
N3
BzO
OBz
BnO
BnO
O
OBn
f1 (ppm)

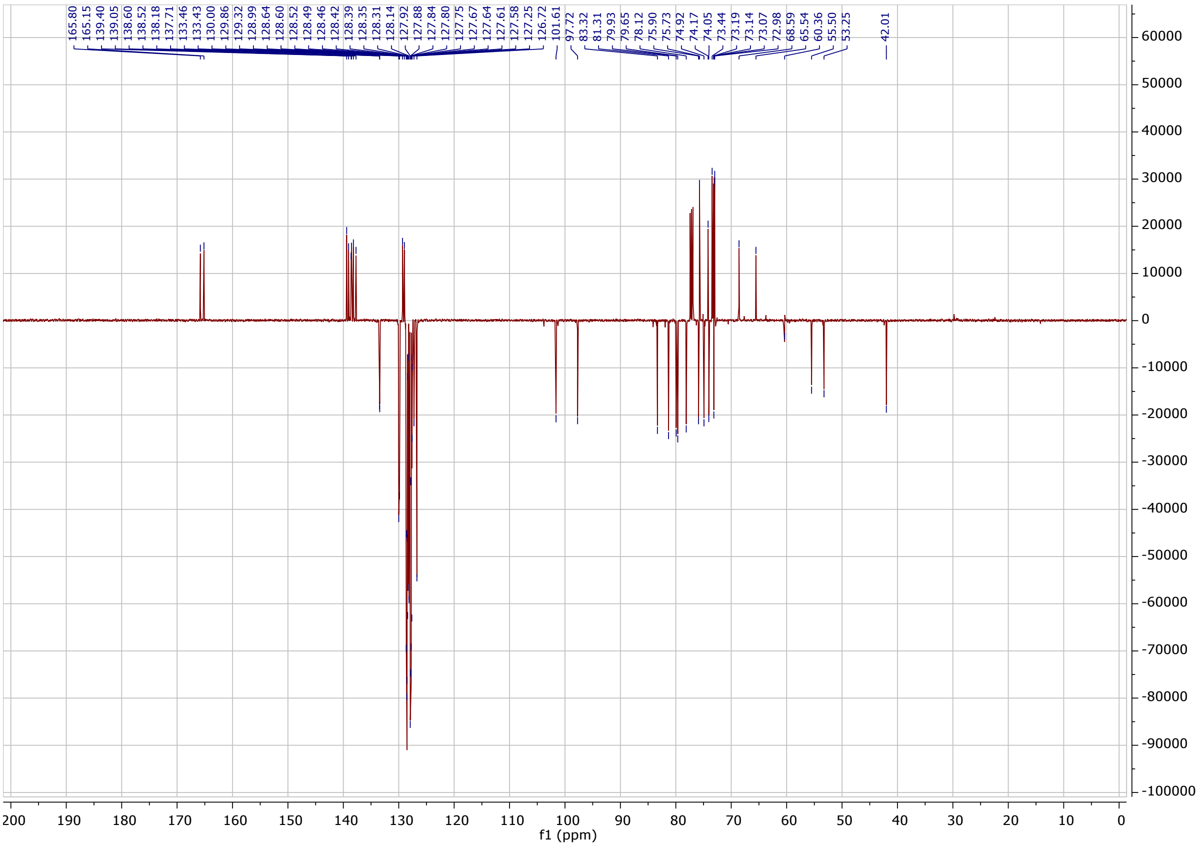
165.80 165.15 139.40 139.05 138.60 138.52 138.18 137.71 133.46 133.43 130.00 129.86 129.32 128.99 128.64 128.60 128.52 128.49 128.46 128.42 128.39 128.35 128.31 128.14 127.92 127.88 127.84 127.80 127.75 127.67 127.64 127.61 127.58 127.25 126.72 101.61 97.72 83.32 81.31 79.93 79.65 78.12 75.90 75.73 74.92 74.17 74.05 73.44 73.19 73.14 73.07 72.98 68.59 65.54 60.36 55.50 53.25 42.01
f1 (ppm)

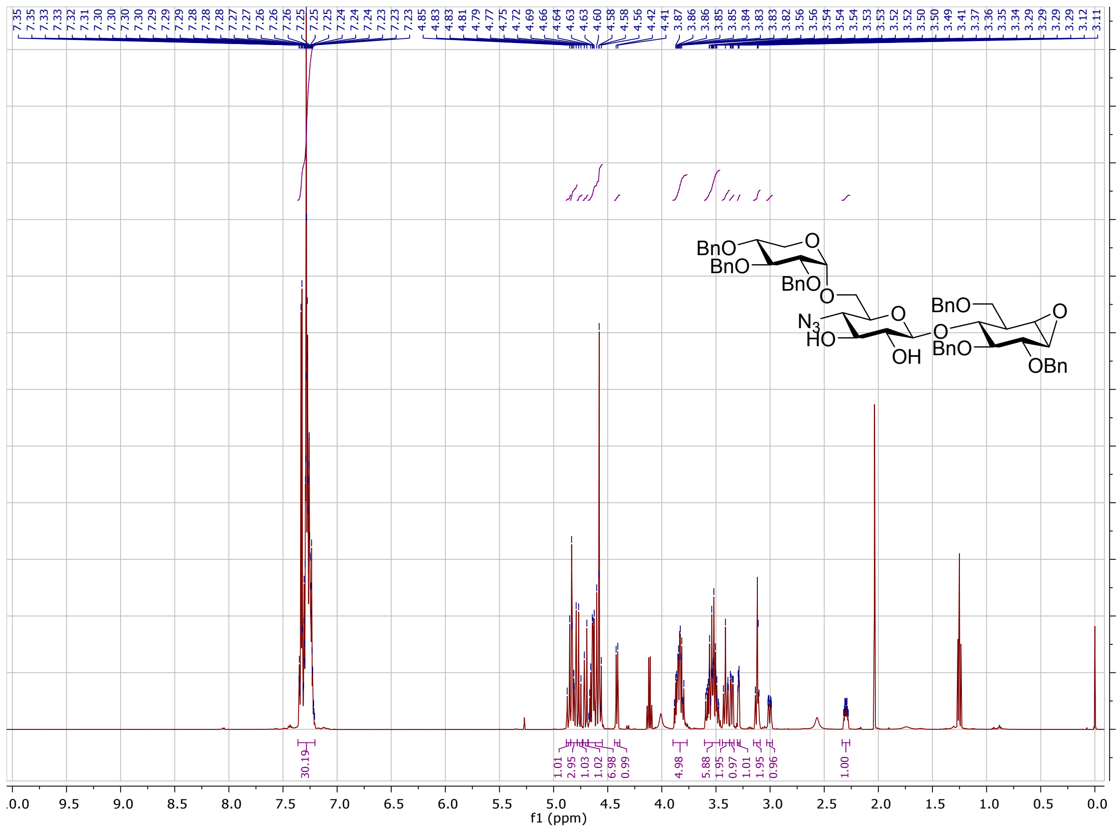
BnO
BnO
BnO
O
N3
HO
OH
BnO
BnO
OBn
f1 (ppm)

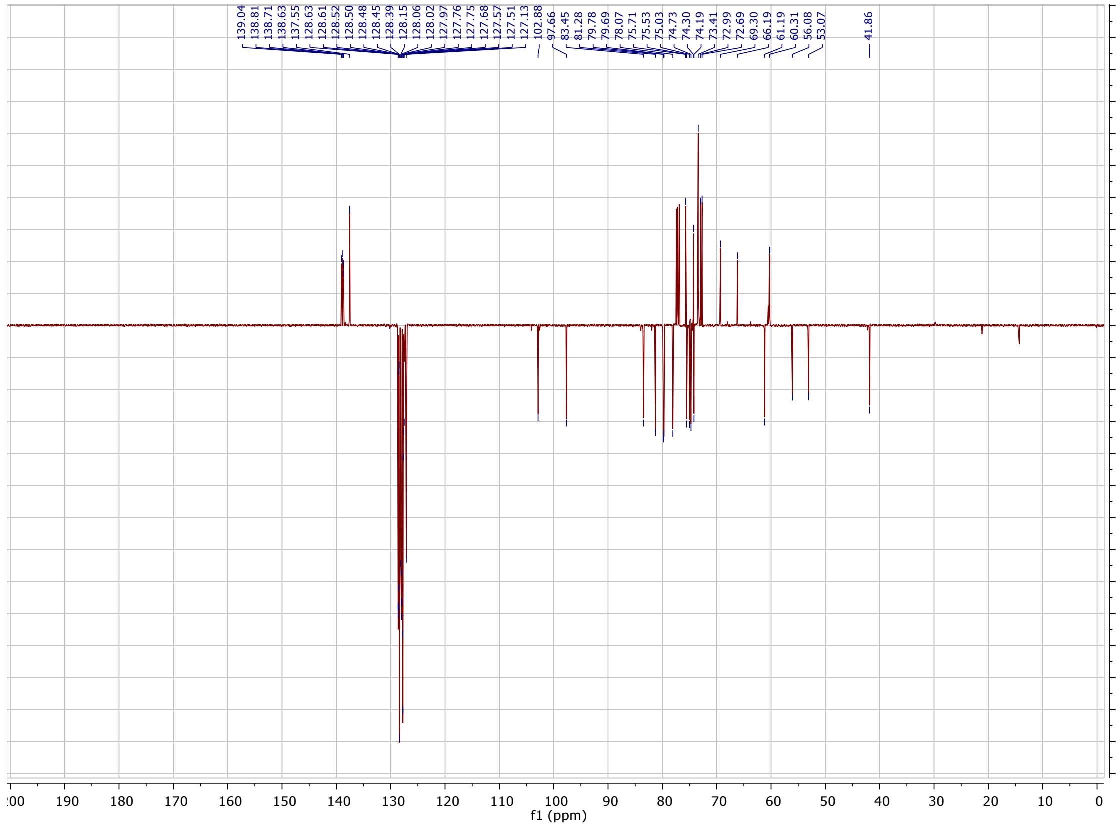
f1 (ppm)

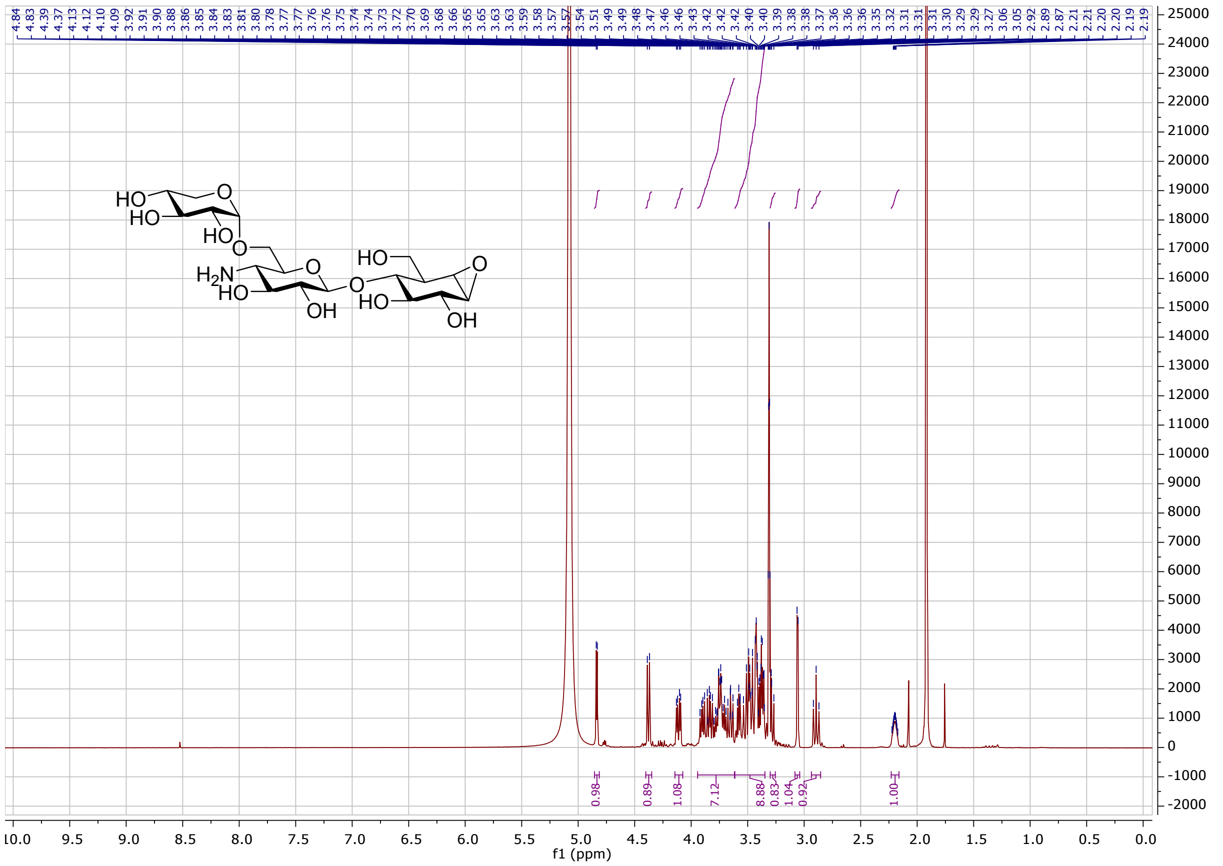
4.84 4.83 4.39 4.37 4.13 4.12 4.10 4.09 3.92 3.91 3.90 3.88 3.86 3.85 3.84 3.83 3.81 3.80 3.78 3.77 3.76 3.76 3.75 3.74 3.74 3.73 3.72 3.70 3.69 3.68 3.66 3.65 3.65 3.63 3.63 3.59 3.58 3.57 3.57 3.54 3.51 3.49 3.49 3.48 3.47 3.46 3.46 3.43 3.42 3.42 3.42 3.40 3.40 3.39 3.38 3.38 3.37 3.36 3.36 3.36 3.35 3.32 3.31 3.31 3.31 3.30 3.29 3.29 3.27 3.06 3.05 2.92 2.89 2.87 2.21 2.21 2.20 2.20 2.19 2.19
0.98 0.89 1.08 7.12 8.88 0.83 1.04 0.92 1.00
HO
HO
HO
O
O
H2N
HO
O
OH
O
HO
HO
O
OH
f1 (ppm)

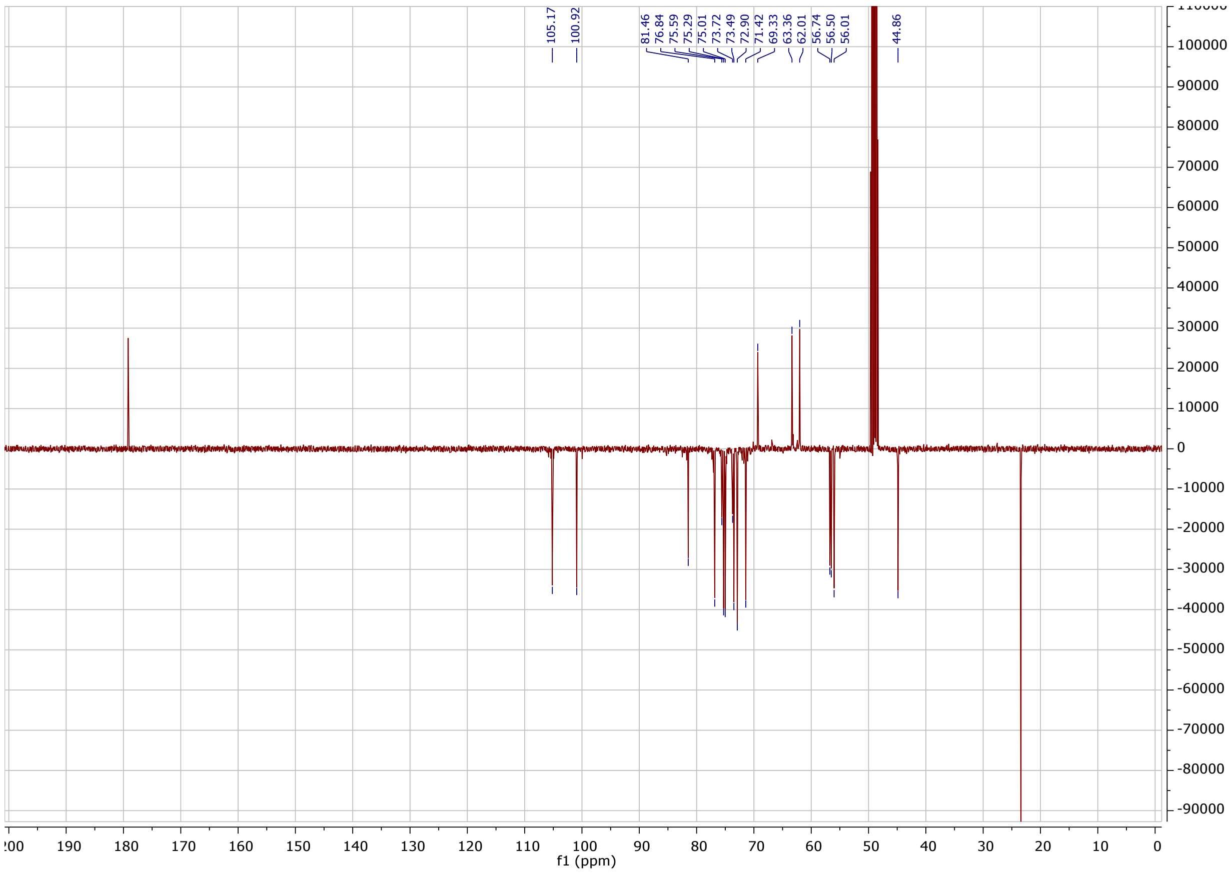
105.17 100.92 81.46 76.84 75.59 75.29 75.01 73.72 73.49 72.90 71.42 69.33 63.36 62.01 56.74 56.50 56.01 44.86
f1 (ppm)

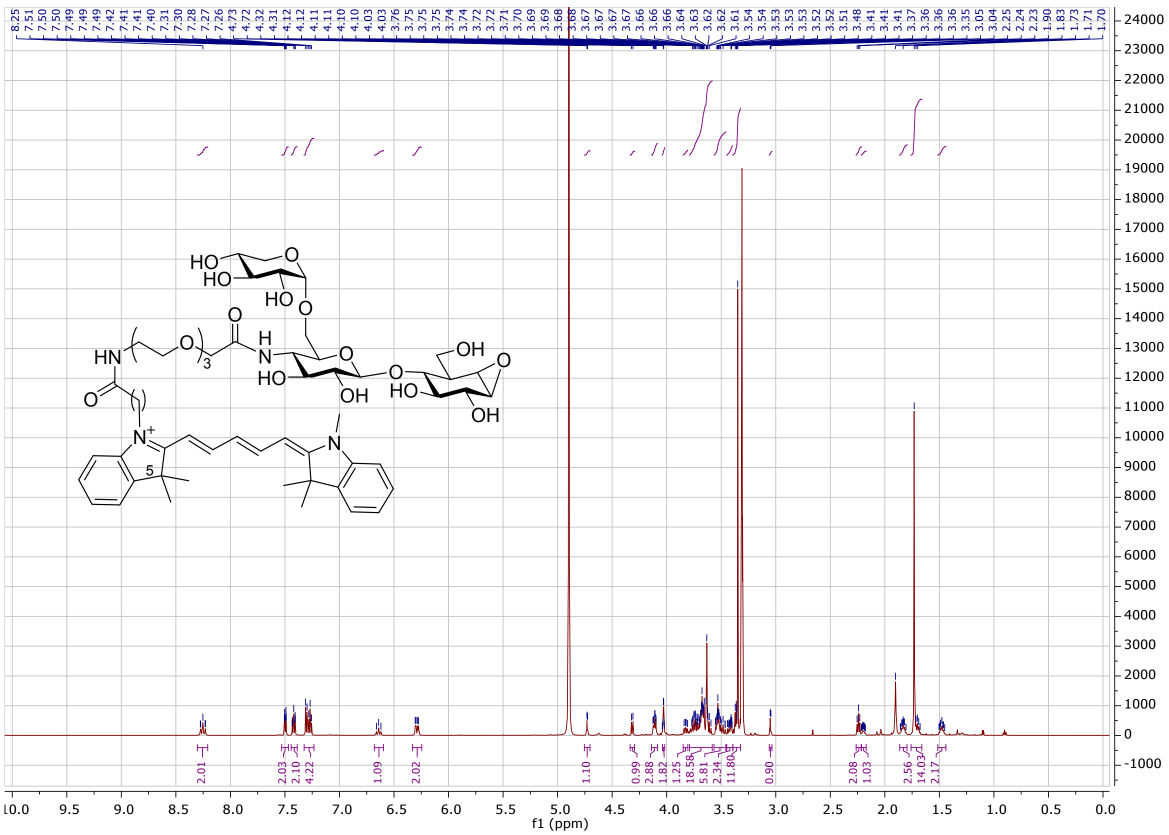
8.25 7.51 7.50 7.50 7.49 7.49 7.49 7.42 7.41 7.41 7.40 7.31 7.30 7.28 7.27 7.26 4.73 4.72 4.32 4.31 4.12 4.12 4.11 4.11 4.10 4.10 4.03 4.03 3.76 3.75 3.75 3.75 3.74 3.74 3.72 3.72 3.71 3.70 3.69 3.69 3.68 3.68 3.67 3.67 3.67 3.67 3.66 3.66 3.66 3.64 3.63 3.62 3.62 3.61 3.61 3.54 3.54 3.54 3.53 3.53 3.53 3.52 3.52 3.51 3.48 3.41 3.41 3.41 3.37 3.36 3.36 3.36 3.35 3.05 3.04 2.25 2.24 2.23 1.90 1.83 1.73 1.71 1.70
2.01 2.03 2.10 4.22 1.09 2.02 1.10 0.99 2.88 1.82 1.25 18.58 5.81 2.34 11.80 0.90 2.08 1.03 2.56 14.03 2.17
HO
HO
HO
O
O
O
HN
O
3
H
N
O
HO
OH
O
OH
HO
O
OH
O
N+
5
N
f1 (ppm)

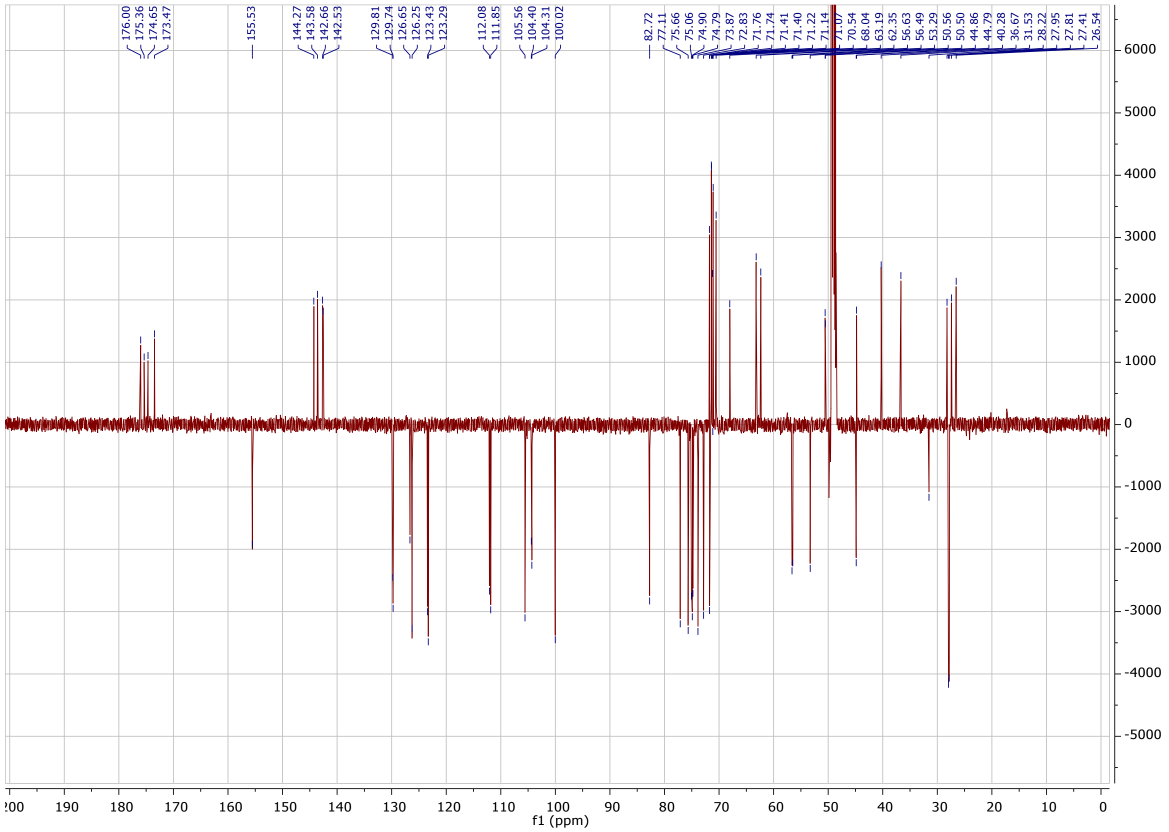
176.00 175.36 174.65 173.47 155.53 144.27 143.58 142.66 142.53 129.81 129.74 126.65 126.25 123.43 123.29 112.08 111.85 105.56 104.40 104.31 100.02 82.72 77.11 75.66 75.06 74.90 74.79 73.87 72.83 71.76 71.74 71.41 71.40 71.22 71.14 71.07 70.54 68.04 63.19 62.35 56.63 56.49 53.29 50.56 50.50 44.86 44.79 40.28 36.67 31.53 28.22 27.95 27.81 27.41 26.54
f1 (ppm)

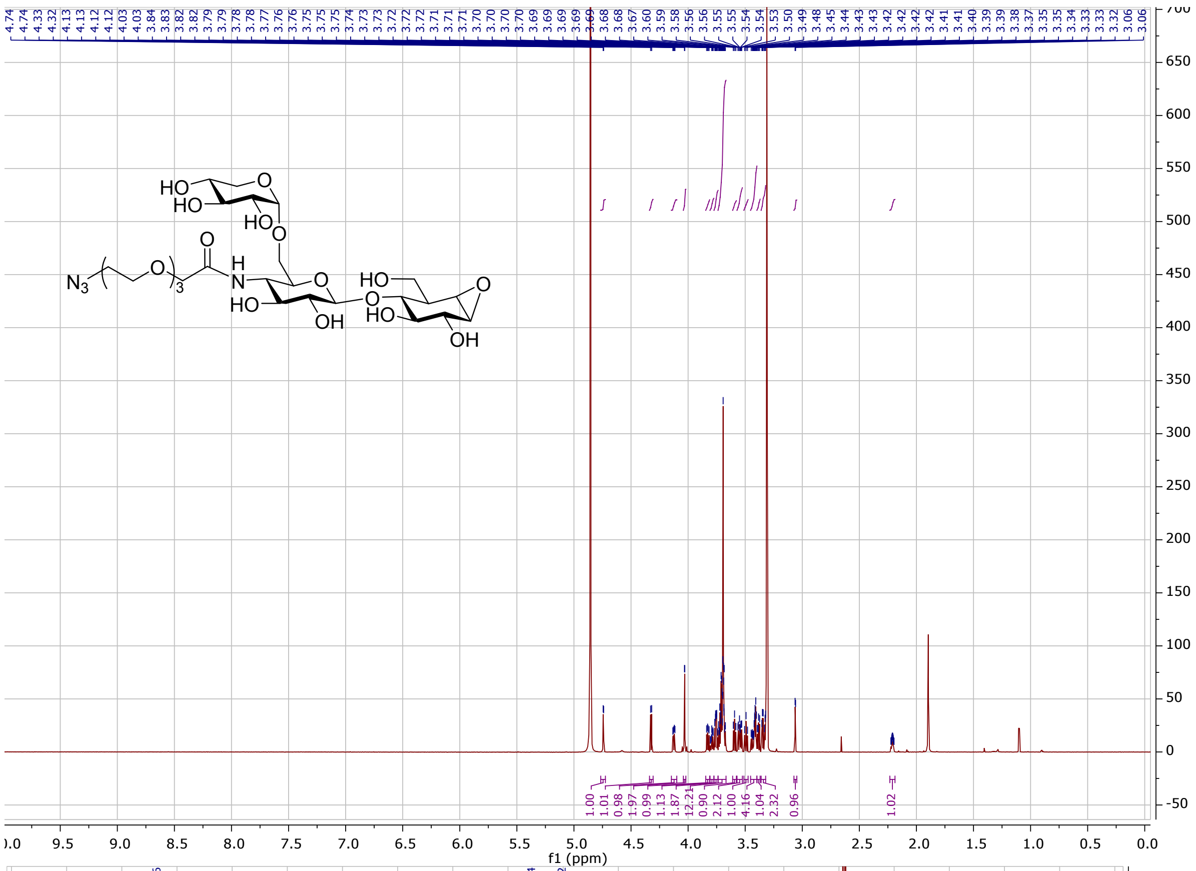
4.74 4.74 4.33 4.32 4.13 4.13 4.12 4.12 4.03 4.03 3.84 3.83 3.82 3.79 3.79 3.78 3.77 3.76 3.75 3.75 3.75 3.74 3.73 3.73 3.72 3.72 3.72 3.71 3.71 3.71 3.70 3.70 3.70 3.69 3.69 3.69 3.69 3.68 3.68 3.67 3.60 3.59 3.58 3.56 3.56 3.55 3.55 3.54 3.54 3.53 3.50 3.49 3.48 3.45 3.44 3.43 3.43 3.42 3.42 3.42 3.42 3.41 3.40 3.39 3.39 3.38 3.37 3.35 3.34 3.33 3.33 3.32 3.06
1.00 1.01 0.98 1.97 0.99 1.13 1.87 12.21 0.90 2.12 1.00 4.16 1.04 2.32 0.96 1.02
f1 (ppm)
HO
HO
HO
O
O
N3
O
3
H
N
HO
O
OH
HO
O
HO
O
OH

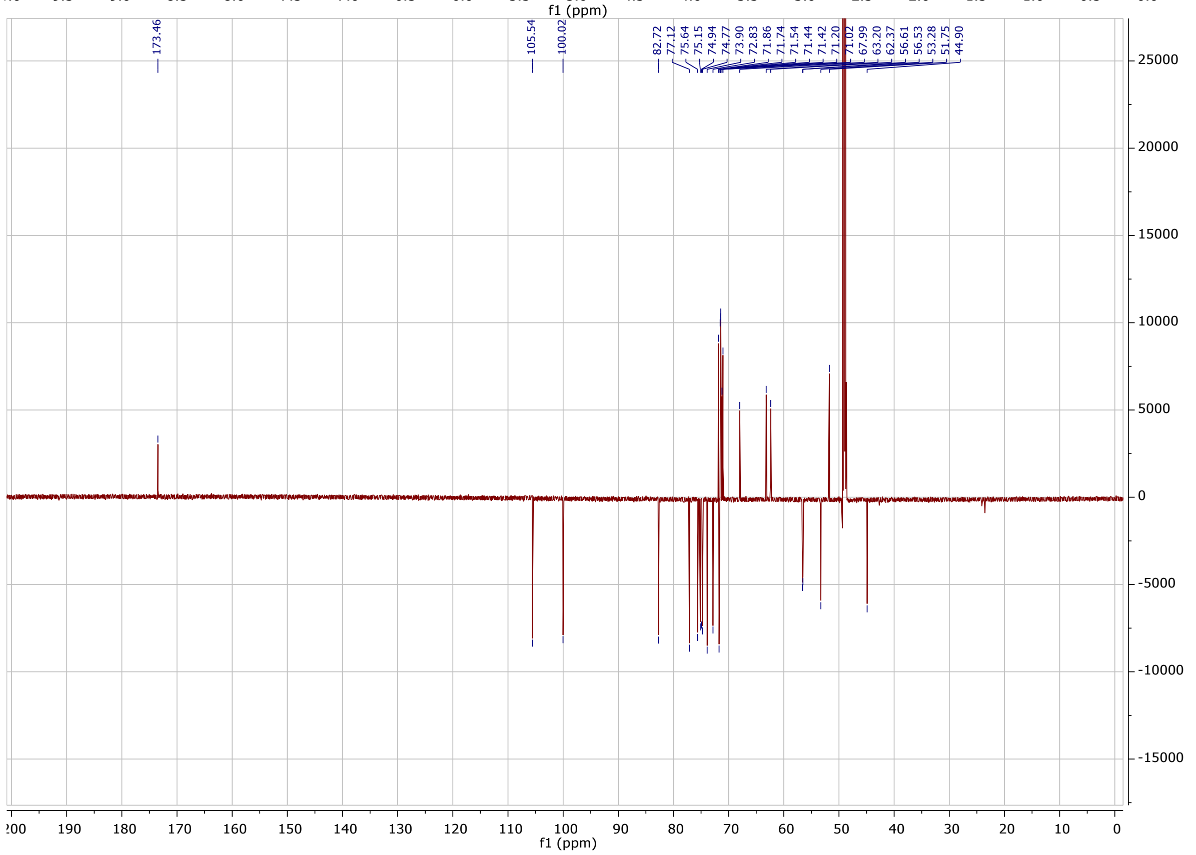
173.46
105.54
100.02
82.72 77.12 75.64 75.15 74.94 74.77 73.90 72.83 71.86 71.74 71.54 71.44 71.42 71.20 71.02 67.99 63.20 62.37 56.61 56.53 53.28 51.75 44.90
f1 (ppm)

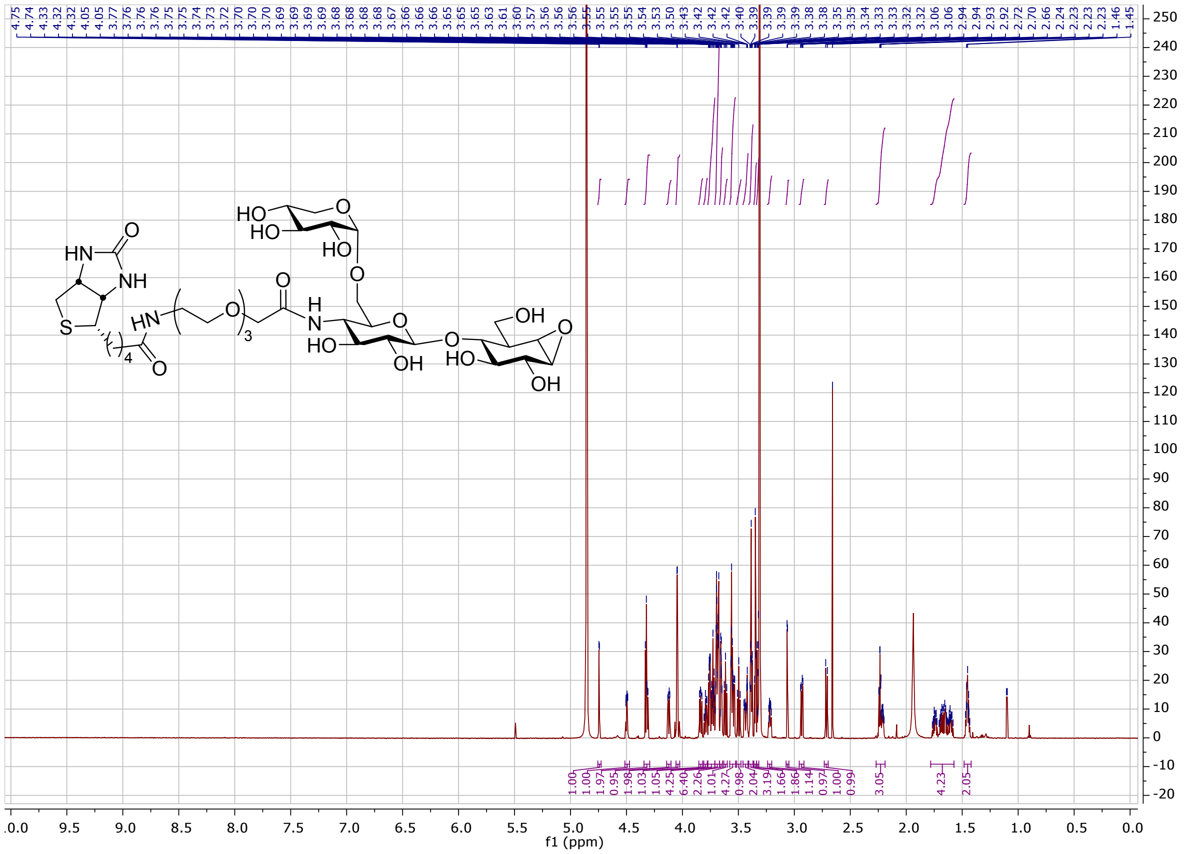
4.75 4.74 4.33 4.32 4.32 4.05 4.05 3.77 3.76 3.76 3.75 3.75 3.74 3.73 3.72 3.70 3.70 3.69 3.69 3.69 3.69 3.68 3.68 3.68 3.68 3.67 3.66 3.66 3.66 3.65 3.65 3.65 3.63 3.61 3.60 3.57 3.56 3.56 3.56 3.55 3.55 3.55 3.55 3.54 3.53 3.50 3.43 3.42 3.42 3.42 3.40 3.39 3.39 3.39 3.39 3.39 3.38 3.38 3.35 3.35 3.34 3.33 3.33 3.32 3.32 3.06 3.06 2.94 2.94 2.93 2.92 2.72 2.70 2.66 2.24 2.23 2.23 2.23 2.23 1.46 1.45
1.00 1.00 1.97 0.95 1.98 1.03 1.05 4.25 6.40 2.26 1.01 4.27 0.98 2.04 3.19 1.66 1.86 1.14 0.97 1.00 0.99 3.05 4.23 2.05
f1 (ppm)

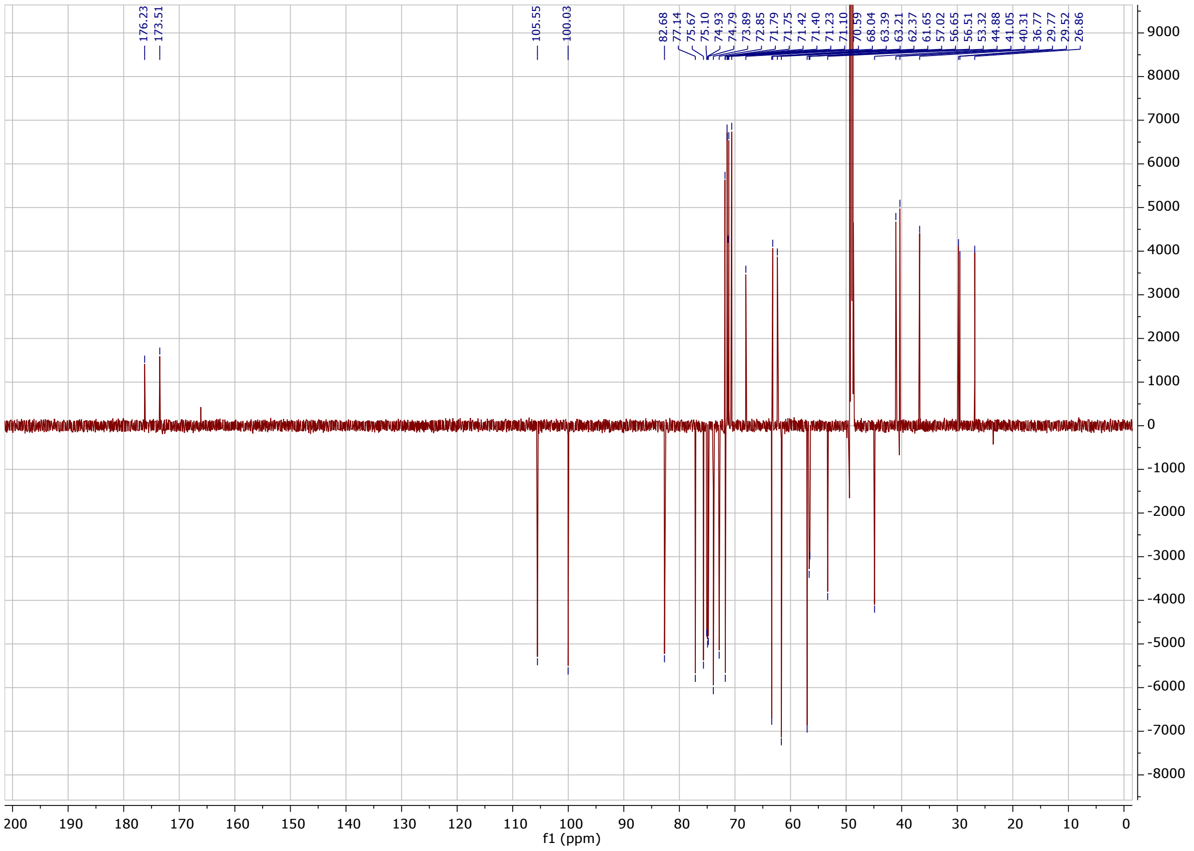
176.23 173.51 105.55 100.03 82.68 77.14 75.67 75.10 74.93 74.79 73.89 72.85 71.79 71.75 71.42 71.40 71.23 71.10 70.59 68.04 63.39 63.21 62.37 61.65 57.02 56.65 56.51 53.32 44.88 41.05 40.31 36.77 29.77 29.52 26.86
f1 (ppm)

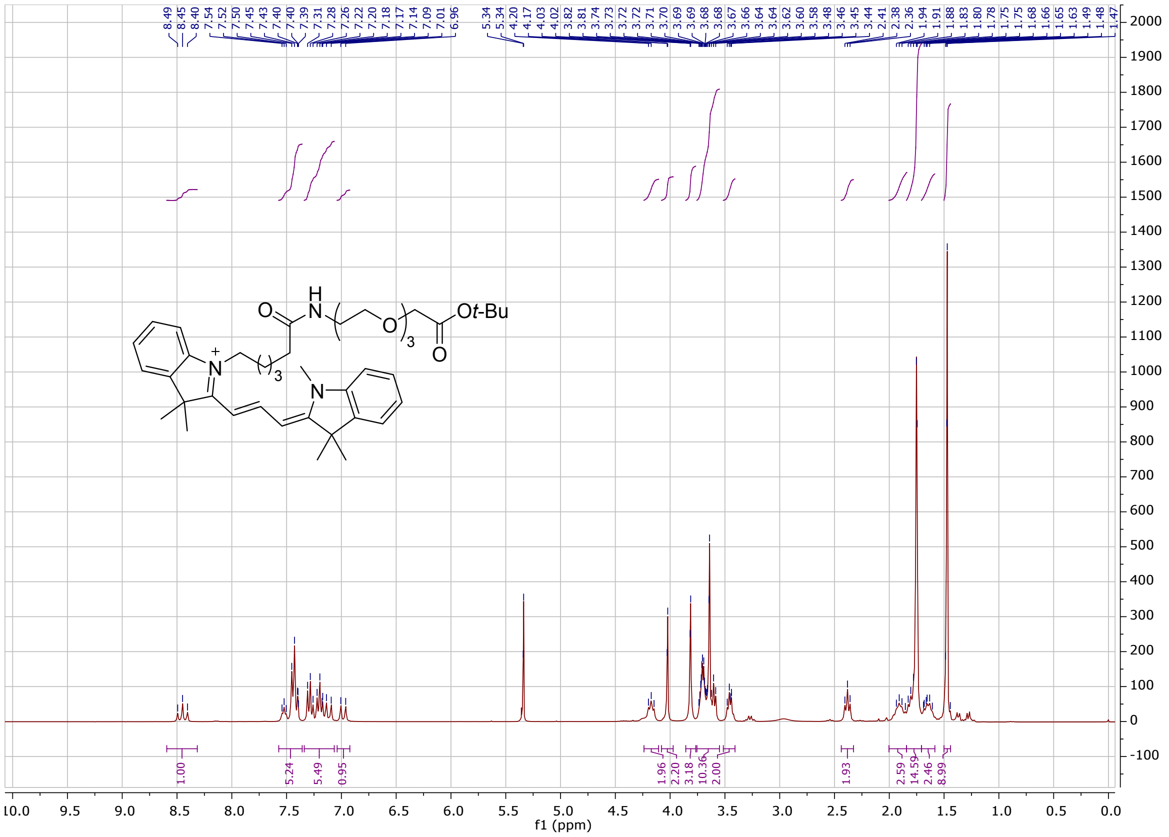
8.49 8.45 8.40 7.54 7.52 7.50 7.45 7.43 7.40 7.40 7.39 7.31 7.28 7.26 7.22 7.20 7.18 7.17 7.14 7.09 7.01 6.96
5.34 5.34 4.20 4.17 4.03 4.02 3.82 3.81 3.74 3.73 3.72 3.72 3.71 3.70 3.69 3.68 3.68 3.67 3.66 3.64 3.64 3.62 3.60 3.58 3.48 3.46 3.45 3.44 2.41 2.38 2.36 1.94 1.91 1.88 1.83 1.80 1.78 1.75 1.68 1.66 1.65 1.63 1.49 1.48 1.47
H
O
N
O
3
Ot-Bu
O
N
+
3
N
1.00
5.24
5.49
0.95
1.96
2.20
3.18
10.36
2.00
1.93
2.59
14.59
2.46
8.99
f1 (ppm)

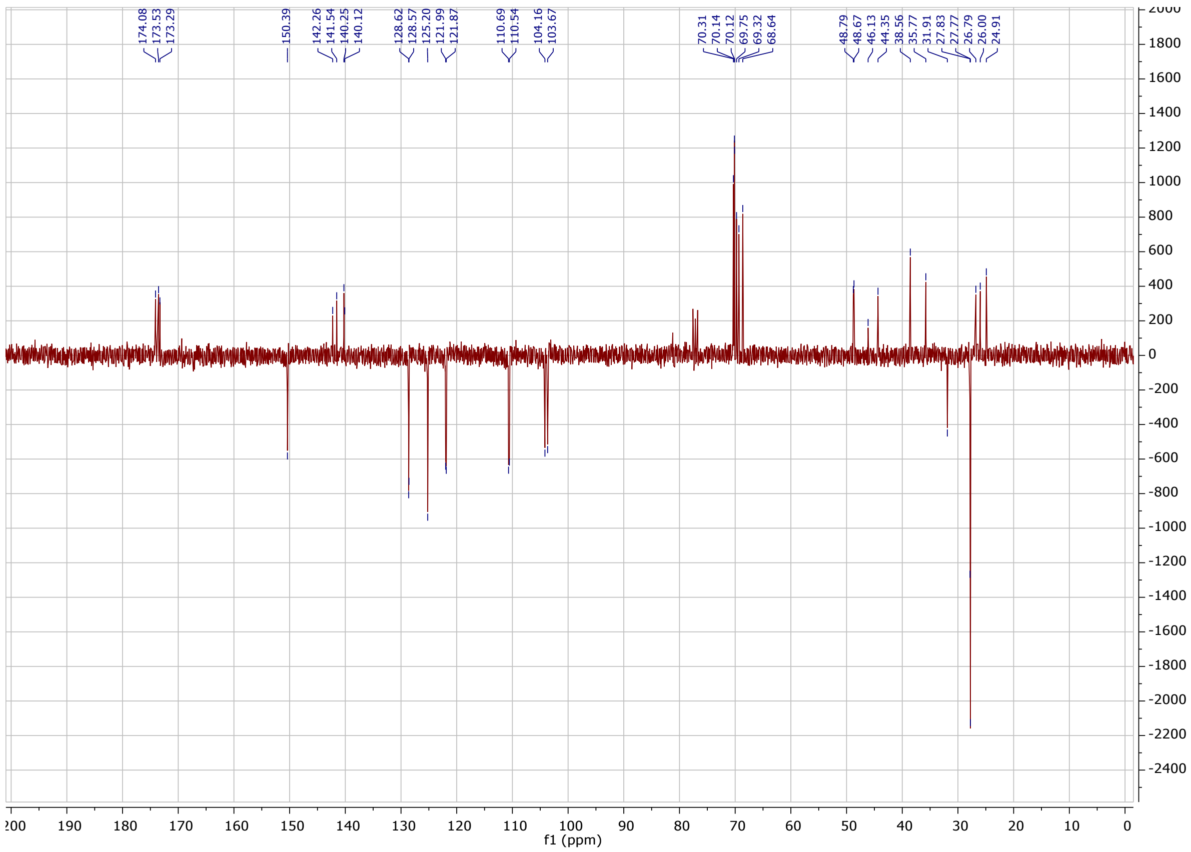
174.08 173.53 173.29
150.39
142.26 141.54 140.25 140.12
128.62 128.57 125.20 121.99 121.87
110.69 110.54
104.16 103.67
70.31 70.14 70.12 69.75 69.32 68.64
48.79 48.67 46.13 44.35 38.56 35.77 31.91 27.83 27.77 26.79 26.00 24.91
f1 (ppm)

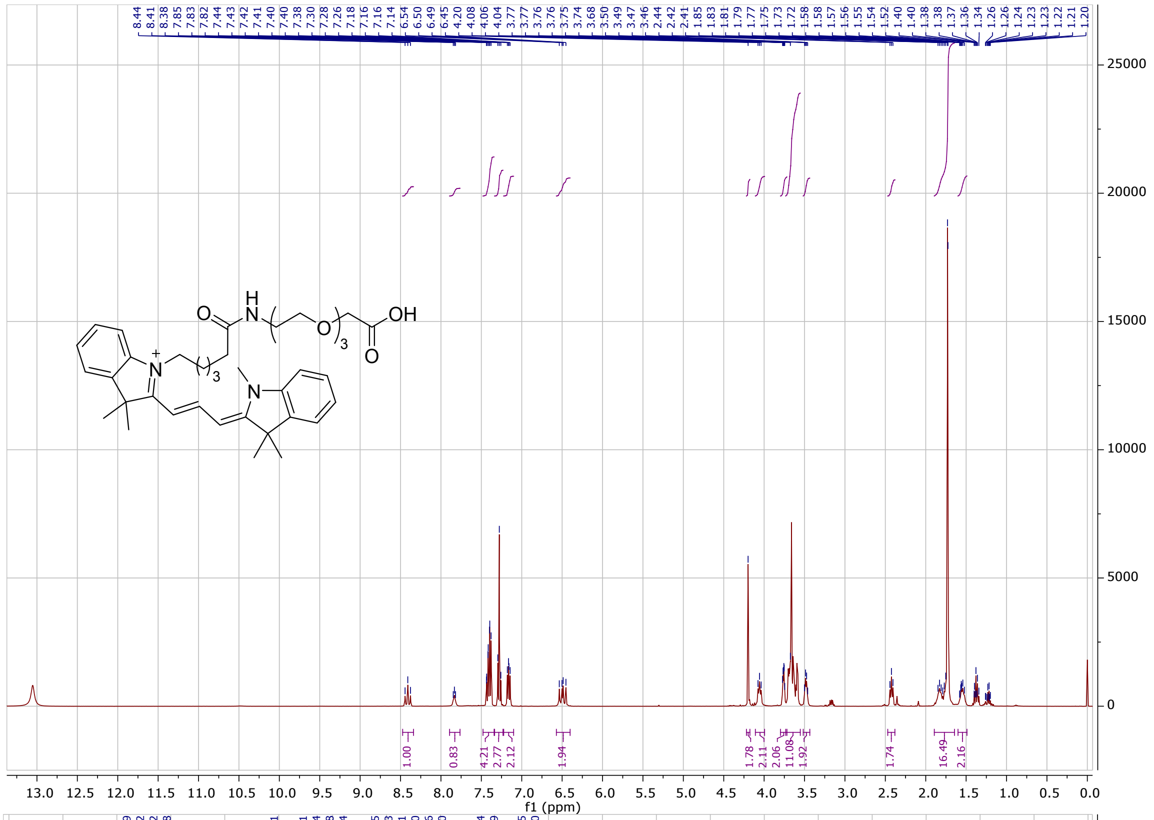
8.44 8.41 8.38 7.85 7.83 7.82 7.44 7.43 7.42 7.41 7.40 7.40 7.38 7.30 7.28 7.26 7.18 7.16 7.16 7.14 6.54 6.50 6.49 6.45 4.20 4.08 4.06 4.04 3.77 3.77 3.76 3.76 3.75 3.74 3.68 3.50 3.49 3.47 3.46 2.44 2.42 2.41 1.85 1.83 1.81 1.79 1.77 1.75 1.73 1.72 1.58 1.58 1.57 1.56 1.55 1.54 1.52 1.40 1.40 1.38 1.38 1.37 1.36 1.34 1.26 1.26 1.24 1.23 1.23 1.22 1.21 1.20
1.00 0.83 4.21 2.77 2.12 1.94 1.78 2.11 2.06 11.08 1.92 1.74 16.49 2.16
f1 (ppm)

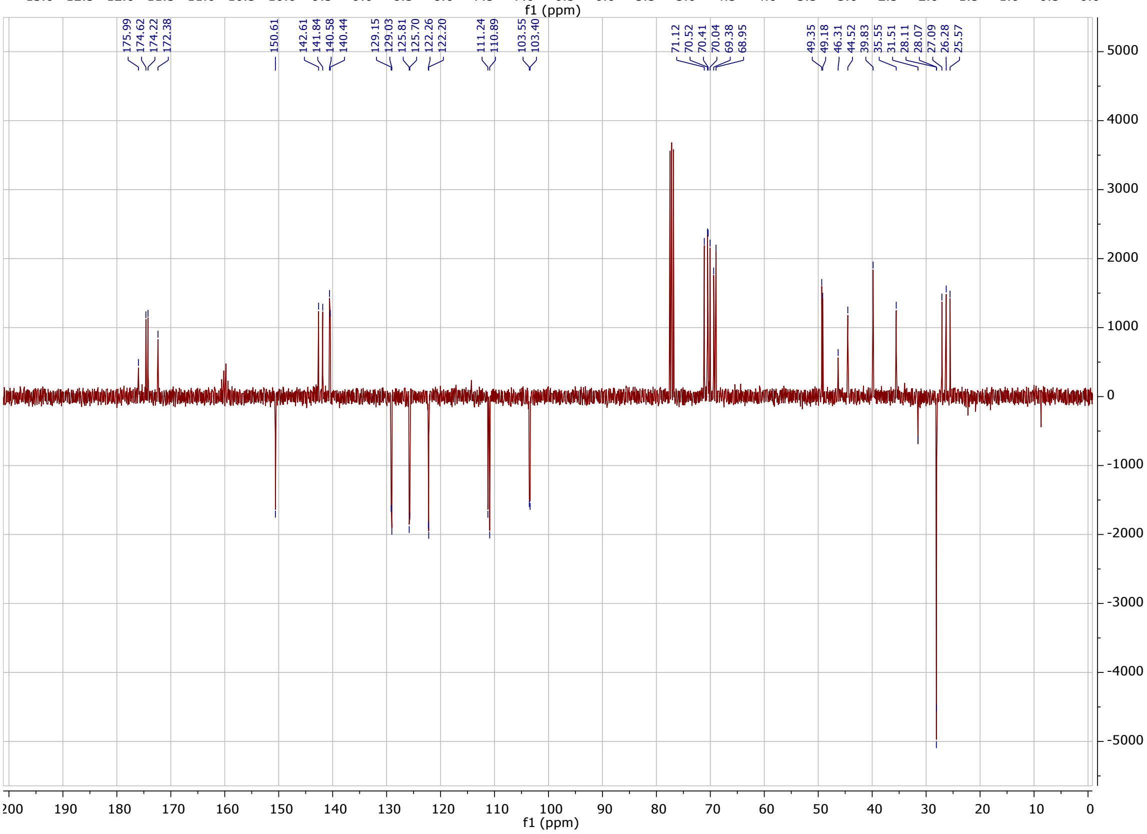
175.99 174.62 174.22 172.38 150.61 142.61 141.84 140.58 140.44 129.15 129.03 125.81 125.70 122.26 122.20 111.24 110.89 103.55 103.40 71.12 70.52 70.41 70.04 69.38 68.95 49.35 49.18 46.31 44.52 39.83 35.55 31.51 28.11 28.07 27.09 26.28 25.57
f1 (ppm)

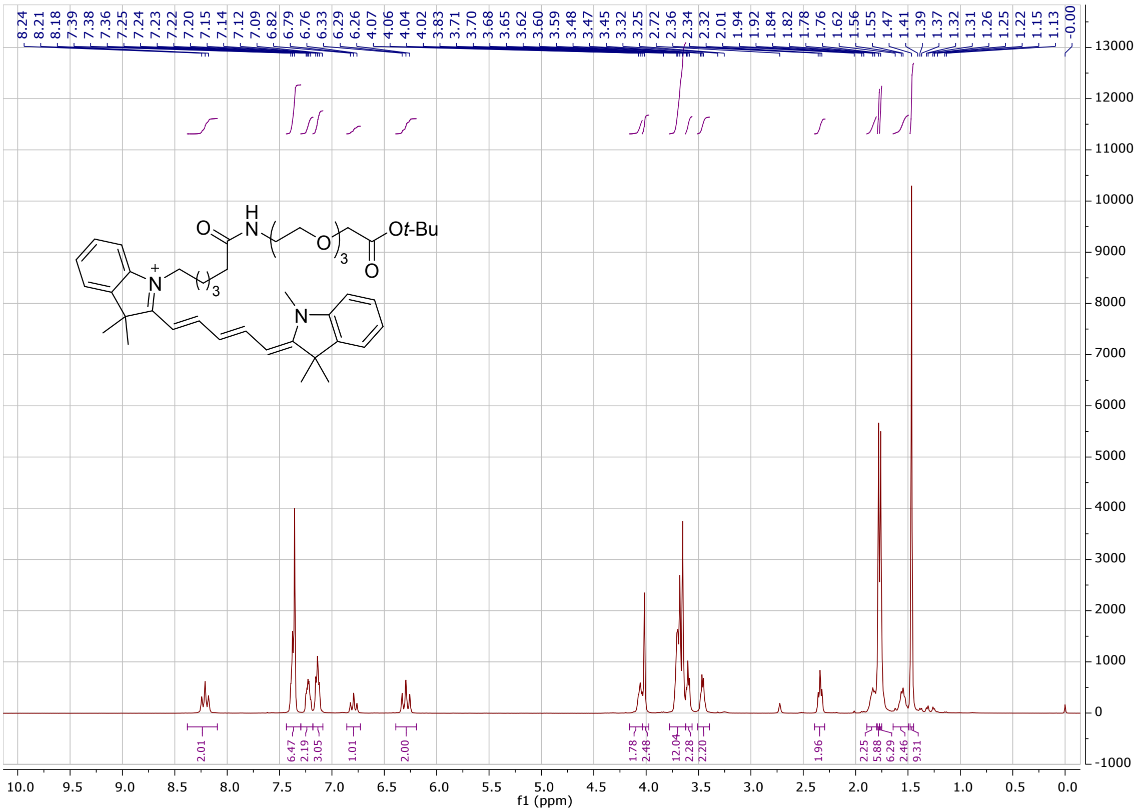
8.24 8.21 8.18 7.39 7.38 7.36 7.25 7.24 7.23 7.22 7.20 7.15 7.14 7.12 7.09 6.82 6.79 6.76 6.33 6.29 6.26 4.07 4.06 4.04 4.02 3.83 3.71 3.70 3.68 3.65 3.62 3.60 3.59 3.48 3.47 3.45 3.32 3.25 2.72 2.36 2.34 2.32 2.01 1.94 1.92 1.84 1.82 1.78 1.76 1.62 1.56 1.55 1.47 1.41 1.39 1.37 1.32 1.31 1.26 1.25 1.22 1.15 1.13 -0.00
O
H
N
O
3
Ot-Bu
O
N
+
3
N
2.01
6.47
2.19
3.05
1.01
2.00
1.78
2.48
12.04
2.28
2.20
1.96
2.25
5.88
6.29
2.46
9.31
f1 (ppm)

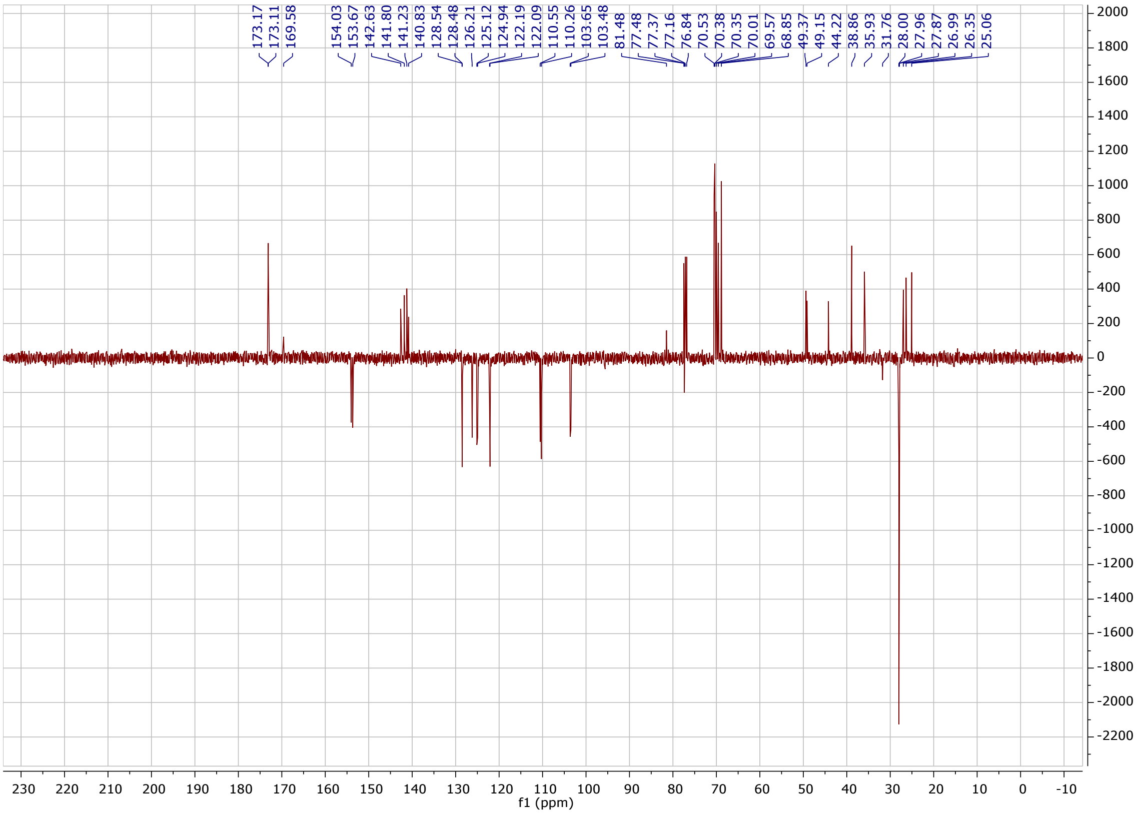
173.17 173.11 169.58 154.03 153.67 142.63 141.80 141.23 140.83 128.54 128.48 126.21 125.12 124.94 122.19 122.09 110.55 110.26 103.65 103.48 81.48 77.48 77.37 77.16 76.84 70.53 70.38 70.35 70.01 69.57 68.85 49.37 49.15 44.22 38.86 35.93 31.76 28.00 27.96 27.87 26.99 26.35 25.06
f1 (ppm)

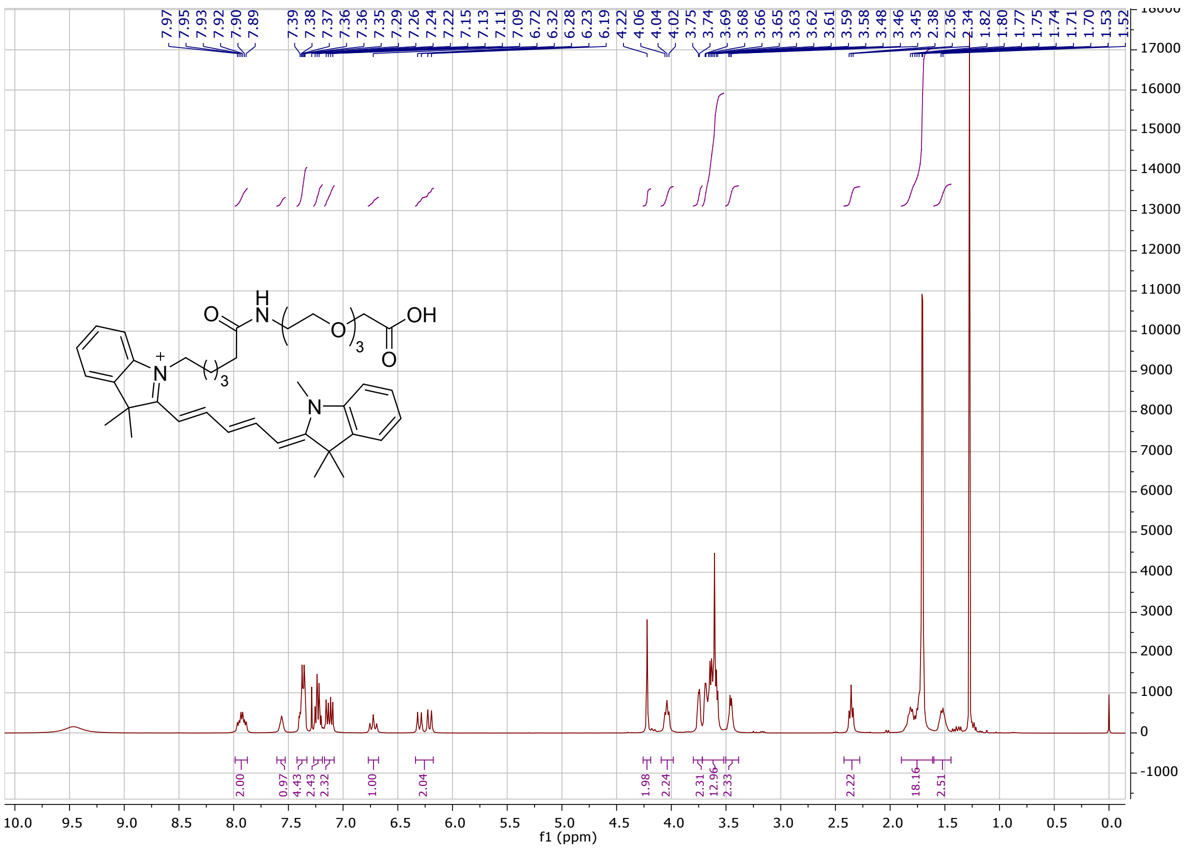
7.97 7.95 7.93 7.92 7.90 7.89 7.39 7.38 7.37 7.36 7.36 7.35 7.29 7.26 7.24 7.22 7.15 7.13 7.11 7.09 6.72 6.32 6.28 6.23 6.19 4.22 4.06 4.04 4.02 3.75 3.74 3.69 3.68 3.66 3.65 3.63 3.62 3.61 3.59 3.58 3.48 3.46 3.45 2.38 2.36 2.34 1.82 1.80 1.77 1.75 1.74 1.71 1.70 1.53 1.52
2.00 0.97 4.43 2.43 2.32 1.00 2.04 1.98 2.24 2.31 12.96 2.33 2.22 18.16 2.51
f1 (ppm)

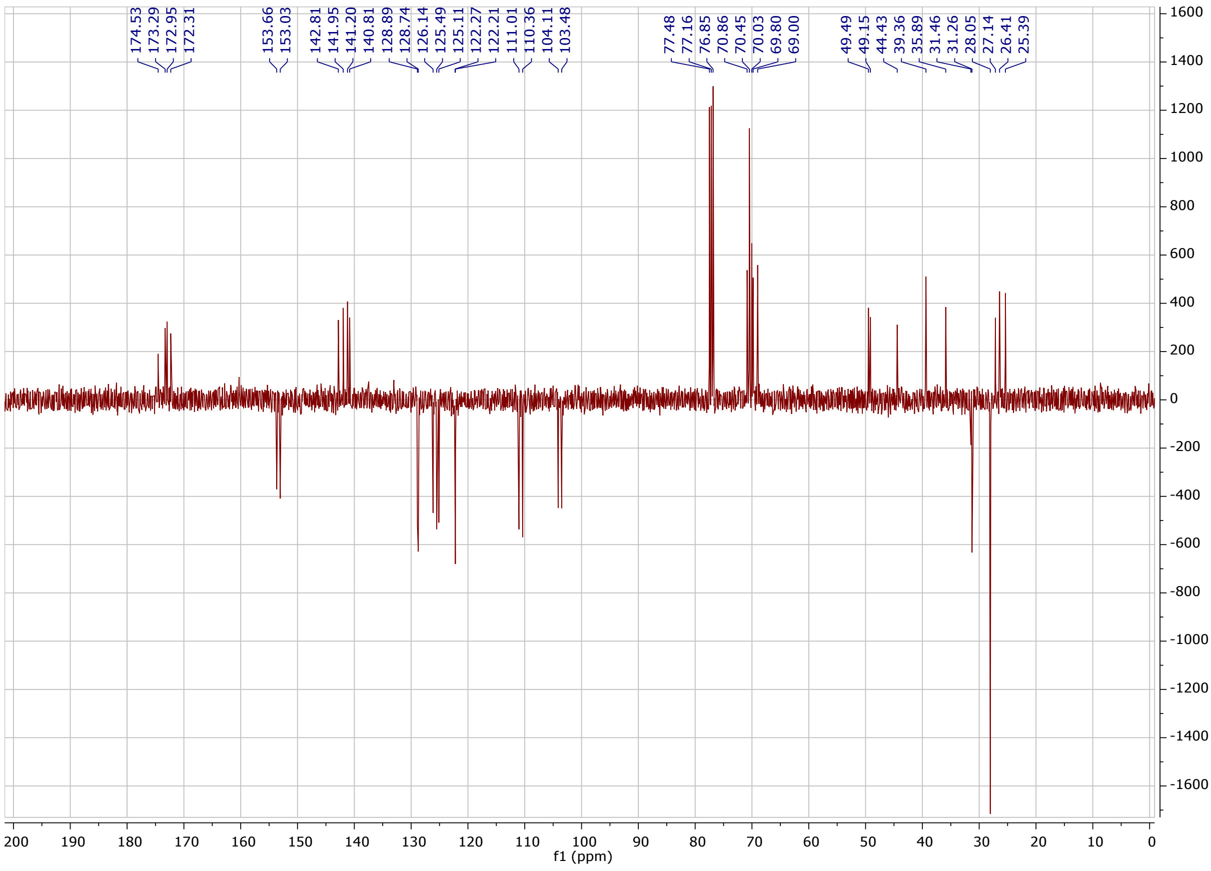
174.53 173.29 172.95 172.31 153.66 153.03 142.81 141.95 141.20 140.81 128.89 128.74 126.14 125.49 125.11 122.27 122.21 111.01 110.36 104.11 103.48 77.48 77.16 76.85 70.86 70.45 70.03 69.80 69.00 49.49 49.15 44.43 39.36 35.89 31.46 31.26 28.05 27.14 26.41 25.39
f1 (ppm)

Synthesis of xyloglucanase chromogenic substrate

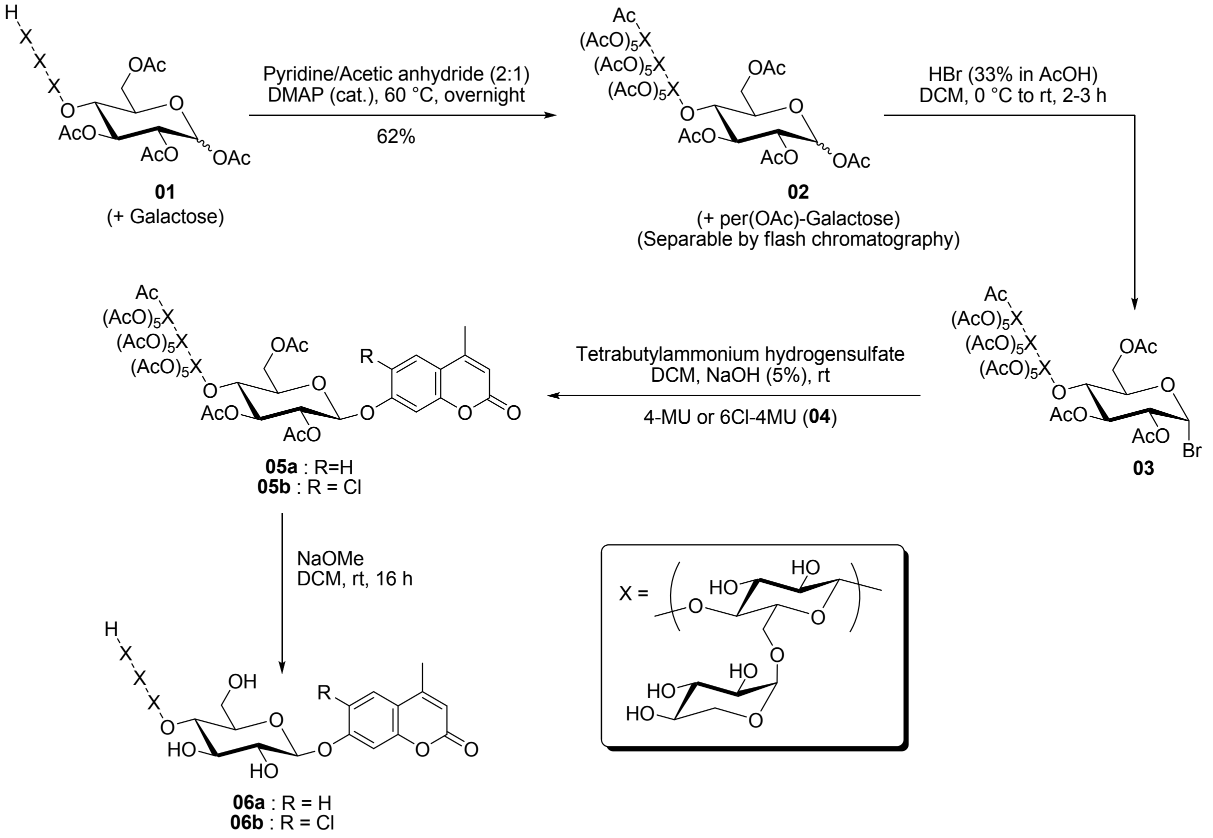

General Methods

All starting materials and reagents were purchased from commercial sources and used as received without further purification. Air- and H_2O-sensitive reactions were performed in oven-dried glassware under N_2 atmosphere. Moisture-sensitive reagents were introduced via a dry syringe. Anhydrous solvents were supplied over molecular sieves and used as received. Reactions were monitored by thin-layer chromatography (TLC) with silica gel 60 F_{254} 0.25 mm pre-coated aluminum foil plates. Compounds were visualized by using UV_{254} and/or a 5% H_2SO_4 staining solution in MeOH followed by heating with a heat gun. Flash column chromatography was performed using Sigma-Aldrich silica gel (60 Å pore size, 35-75 µm particle size). Reverse phase chromatography was performed using Sigma-Aldrich Supelco Discovery® DSC-18 SPE tubes (5g, 20 mL – C18 bonding, 70 Å pore size). NMR experiments were recorded with a Jeol 400 MHz NMR spectrometer at 400 MHz for ^1H nuclei and at 100 MHz for ^{13}C nuclei. HRMS were recorded with a Bruker compact® time of flight mass spectrometer.

Preparation of xyloglucan oligosaccharides

Xyloglucan oligosaccharides were prepared similarly using a method derived from.[24] Briefly, 20 g of tamarind gum (Tokyo Chemical Industry) was suspended in 200 mL of ultrapure water in a 1 L Erlenmeyer flask and autoclaved. The resulting highly viscous solution was cooled to 30 °C, then supplemented with 1 mL of 1 M NaOAc buffer pH 6.0 and 7.8 mg of BoGH5A in 100 µL of 20 mM MOPS pH 7.5. The reaction was stirred manually for 5 minutes, then shaken at 200 RPM for 2 hours. The resulting low-viscosity solution was centrifuged (3000xg, 5 minutes) to remove insoluble material. The supernatant was vacuum filtered (0.45 µm cut-off) into a clean bottle and a sample of xyloglucan oligosaccharides was taken for analysis. 6.4 mg of CjBgl35A (produced as described previously[25]) was then added and the reaction was stirred overnight at 30 °C. Enzymes were inactivated and the solution was sterilized by heating to a boil for 2 minutes. The quality of the resulting oligosaccharides was assessed by HPAEC-PAD (Supplemental Figure 1), showing XXXG and galactose as the major constituents. The resulting ~200 mL clear, colourless solution was stored at -20°C prior to lyophilization.

Synthesis of per-*O*-acetylated-XXXG 02[26]

The XXXG and galactose mixture **01** (3.70 g) was acetylated in a pyridine/acetic anhydride mixture (2:1 v/v – 60 mL) in the presence of a catalytic amount of DMAP (10 mg) with constant stirring overnight at 60 °C under N_2 atmosphere. After 18 h, the mixture was cooled to 0 °C, quenched with MeOH and concentrated *in vacuo*. The crude residue was dissolved in CH_2Cl_2 (150 mL) and washed successively with a 5% H_2SO_4 aqueous solution (100 mL), a saturated $NaHCO_3$ solution (100 mL) and brine (100 mL). The organic layer was dried over $MgSO_4$ and concentrated *in vacuo*. Purification by flash chromatography (silica gel, Toluene/Acetone 7:3) afforded the per-(OAc)-XXXG **02** (3.58 mg, 54%, amorphous solid).

Analytical data were in agreement with the literature.[27]

Synthesis of 6-chloro-4-methylumbelliferone 04[28]

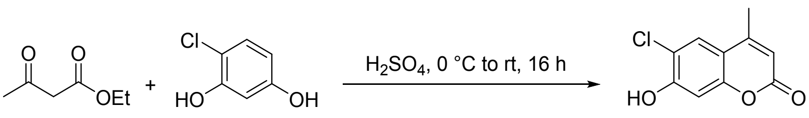

6Cl-4MU **04**

4-chlororesorcinol (1.29 g, 8.92 mmol) was dissolved in ethyl acetoacetate (1.25 mL, 9.82 mmol, 1.1 eq.). The mixture was cooled to 0 °C and 75% H_2SO_4 in ice-water (9 mL) was added. The mixture was stirred overnight at room temperature then poured into well-stirred ice-water. The solid residue was then separated, washed with water, and air-dried. Recrystallization from MeOH afforded compound **04** as an amorphous solid (1.35 g, 72%).

Analytical data were in agreement with the literature.[29]

Synthesis of 4-methylumbelliferyl-XXXG-β-glycoside 06a

The per-*O*-acetylated-XXXG **02** (500 mg, 0.26 mmol) was dissolved in dry CH_2Cl_2 (5 mL) under N_2 atmosphere, and the resulting mixture was cooled to 0 °C. HBr (33% solution in AcOH – 1.7 mL, 9.72 mmol, 37 eq.) was added dropwise and the mixture was stirred for 3 h at room temperature. The mixture was then carefully poured into ice-cold water (30 mL) and the resulting mixture was extracted with CH_2Cl_2 (30 mL). The organic layer was successively washed with an ice-cold saturated solution of $NaHCO_3$ (20 mL) and brine (20 mL). The organic layer was dried over $MgSO_4$ and concentrated *in vacuo*.

4-methylumbelliferone (138 mg, 0.79 mmol, 3 eq.) and TBAHS (89 mg, 0.26 mmol, 1 eq.) were dissolved in NaOH (1M in water, 2 mL). The mixture was stirred at room temperature for 1 h. A solution of the previous crude residue in CH_2Cl_2 (2 mL) was added. The mixture was vigorously stirred at room temperature overnight. After 16 h at room temperature, the mixture was diluted with CH_2Cl_2 (10 mL) and poured into water (20 mL). The aqueous layer was extracted with CH_2Cl_2 (3x10 mL) and the combined organic layers were dried over $MgSO_4$ and concentrated *in vacuo*.

The crude residue was re-acetylated in a 2:1 mixture of pyridine/Ac_2O (9 mL) with stirring overnight at room temperature with a catalytic amount of DMAP (2 mg). After 16 h, the mixture was cooled to 0 °C, quenched with MeOH, and the solvents were removed *in vacuo*. The crude residue was dissolved in CH_2Cl_2 (20 mL) and successively washed with a 5% H_2SO_4 aqueous solution (20 mL), a saturated $NaHCO_3$ solution (20 mL) and brine (20 mL). The organic layer was dried over $MgSO_4$ and concentrated *in vacuo*. Purification by flash chromatography (silica gel, Toluene/Acetone 7:3) afforded the per-*O*-acetylated-4-methylumbelliferyl-XXXG-β-glycoside **05a** (321 mg) as a colorless foam.

The per-*O*-acetylated-4-methylumbelliferyl-XXXG-β-glycoside **05a** (122 mg, 0.06 mmol) was dissolved in dry CH_2Cl_2 (2 mL) under N_2 atmosphere. MeONa (0.5 M solution in MeOH, 2 mL) was added, and the mixture was stirred at room temperature for 3 h. The mixture was then cooled to 0 °C, diluted with MeOH (10 mL) and neutralized with AcOH. The solvents were removed *in vacuo* and the crude residue was dissolved in water (1-2 mL) and purified by reverse-phase chromatography using a 5 mL DSC-18 cartridge (Discovery®) and stepwise elution by a gradient of acetonitrile in water, affording compound **06a** (26 mg, 35%, colorless waxy solid). **^{1}H NMR (400 MHz, D_2O)**: δ 7.56 (d, *J* = 8.9 Hz), 7.01 (d, *J* = 8.9 Hz), 6.90 (s), 6.10 (s), 5.14 (d, *J* = 7.6 Hz), 4.96-4.91 (m), 4.58-5.50 (m), 4.02-3.30 (m), 2.32 (s). **^{13}C NMR (100 MHz, D_2O)**: δ 203.0, 164.3, 159.4, 156.1, 153.7, 126.6, 115.1, 113.9, 111.3, 102.6, 99.5, 98.9, 98.3, 74.1-59.8, 18.0. **HRMS (ESI+) m/z**: $[M+Na]^+$ calc. for C49H72NaO35: 1243.3746, found: 1243.3775.

Synthesis of 6-chloro-4-methylumbelliferyl-XXXG-β-glycoside 06b

The per-*O*-acetylated-XXXG **02** (500 mg, 0.26 mmol) was dissolved in dry CH_2Cl_2 (5 mL) under N_2 atmosphere, and the resulting mixture was cooled to 0 °C. HBr (33% solution in AcOH – 1.7 mL, 9.72 mmol, 37 eq.) was added dropwise and the mixture was stirred for 3 h at room temperature. The mixture was then carefully poured into ice-cold water (30 mL) and the resulting mixture was extracted with CH_2Cl_2 (30 mL). The organic layer was successively washed with an ice-cold saturated solution of $NaHCO_3$ (20 mL) and brine (20 mL). The organic layer was dried over $MgSO_4$ and concentrated *in vacuo*.

6-chloro-4-methylumbelliferone **04** (166 mg, 0.79 mmol, 3 eq.) and TBAHS (89 mg, 0.26 mmol, 1 eq.) were dissolved in NaOH (1M in water, 2 mL). The mixture was stirred at room temperature for 1 h. S solution of the previous crude residue in CH_2Cl_2 (2 mL) was added. The mixture was vigorously stirred at room temperature overnight. After 16 h at room temperature, the mixture was diluted with CH_2Cl_2 (10 mL) and poured into water (20 mL). The aqueous layer was extracted with CH_2Cl_2 (3x10 mL) and the combined organic layers were dried over $MgSO_4$ and concentrated *in vacuo*.

The crude residue was re-acetylated in a 2:1 mixture of pyridine/Ac_2O (9 mL) with stirring overnight at room temperature with a catalytic amount of DMAP (2 mg). After 16 h, the mixture was cooled to 0 °C, quenched with MeOH, and the solvents were removed *in vacuo*. The crude residue was dissolved in CH_2Cl_2 (20 mL) and successively washed with a 5% H_2SO_4 aqueous solution (20 mL), a saturated $NaHCO_3$ solution (20 mL) and brine (20 mL). The organic layer was dried over $MgSO_4$ and concentrated *in vacuo*. Purification by flash chromatography (silica gel, Toluene/Acetone 7:3) afforded the per-*O*-acetylated-6-chloro-4-methylumbelliferyl-XXXG-β-glycoside **05b** (379 mg) as a colorless foam.

The per-*O*-acetylated-6-chloro-4-methylumbelliferyl-XXXG-β-glycoside **05b** (204 mg, 0.10 mmol) was dissolved in dry CH_2Cl_2 (2 mL) under N_2 atmosphere. MeONa (0.5 M solution in MeOH, 2 mL) was added, and the mixture was stirred at room temperature for 3 h. The mixture was then cooled to 0 °C, diluted with MeOH (10 mL) and neutralized with AcOH. The solvents were removed *in vacuo* and the crude residue was dissolved in water (1-2 mL) and purified by reverse-phase chromatography using a 5 mL DSC-18 cartridge (Discovery®) and stepwise elution by a gradient of acetonitrile in water, affording compound **06b** (36 mg, 29%, brown waxy solid). **^{1}H NMR (400 MHz, D_2O)**: δ 7.51 (bs), 6.92 (bs), 6.06 (bs), 5.08 (bd, *J* = 6.3), 4.96-4.91 (m), 4.59-4.48 (m), 4.02-3.28 (m), 2.19 (bs). **^{13}C NMR (100 MHz, D_2O)**: δ 163.3, 154.7, 154.5, 151.9, 132.7, 126.1, 119.6, 115.2, 113.0, 112.0, 103.8-98.3, 79.5-59.8, 17.9. **HRMS (ESI+) m/z**: $[M+Na]^+$ calc. for C49H72ClNaO35: 1277.3357, found: 1277.3418.

1H NMR spectrum (400 MHz, D_2O): 4methylumbelliferyl-XXXG-β-glycoside 06a:

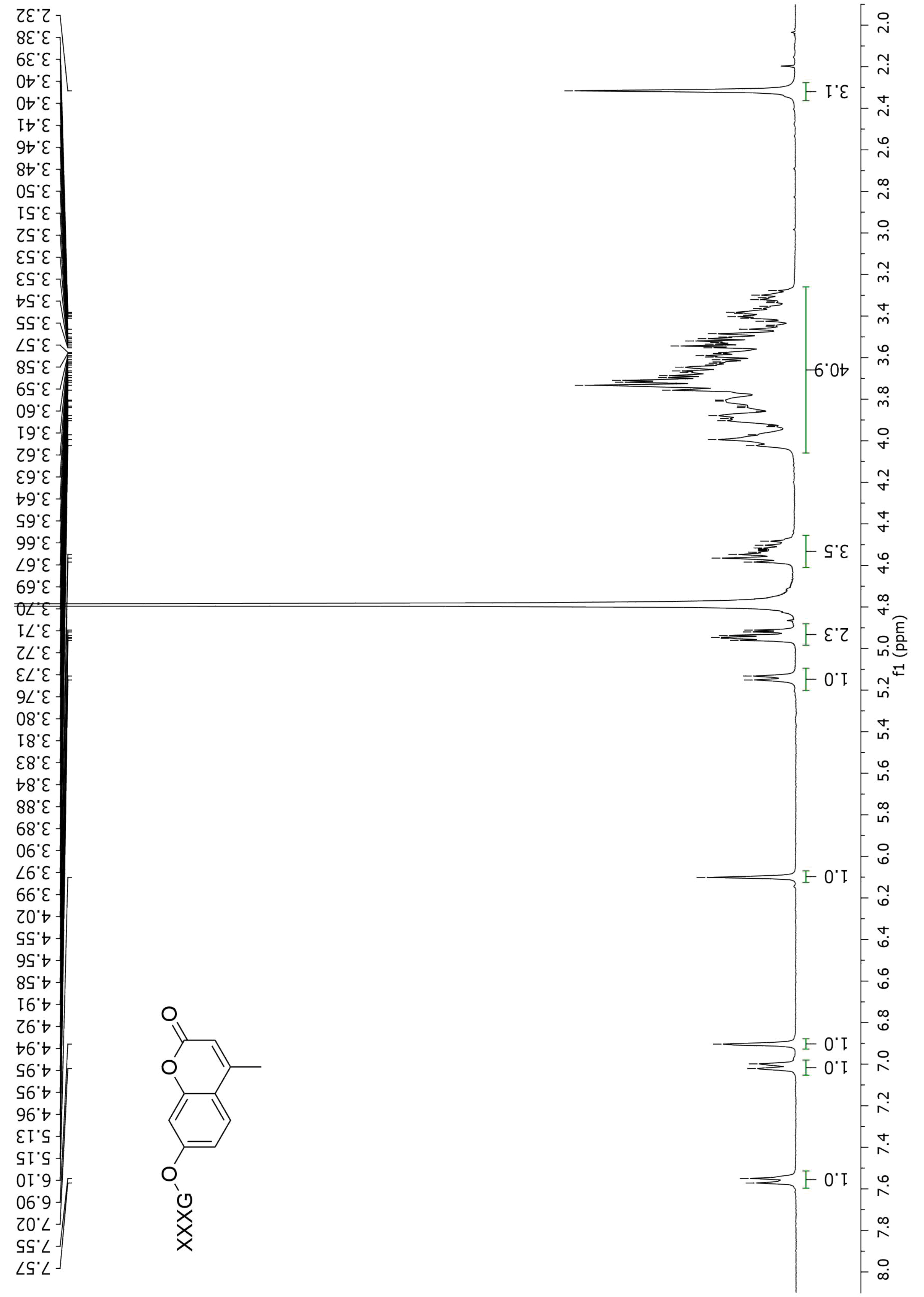

1H NMR spectrum (400 MHz, D_2O): 6-chloro-4methylumbelliferyl-XXXG-β-glycoside 06a:

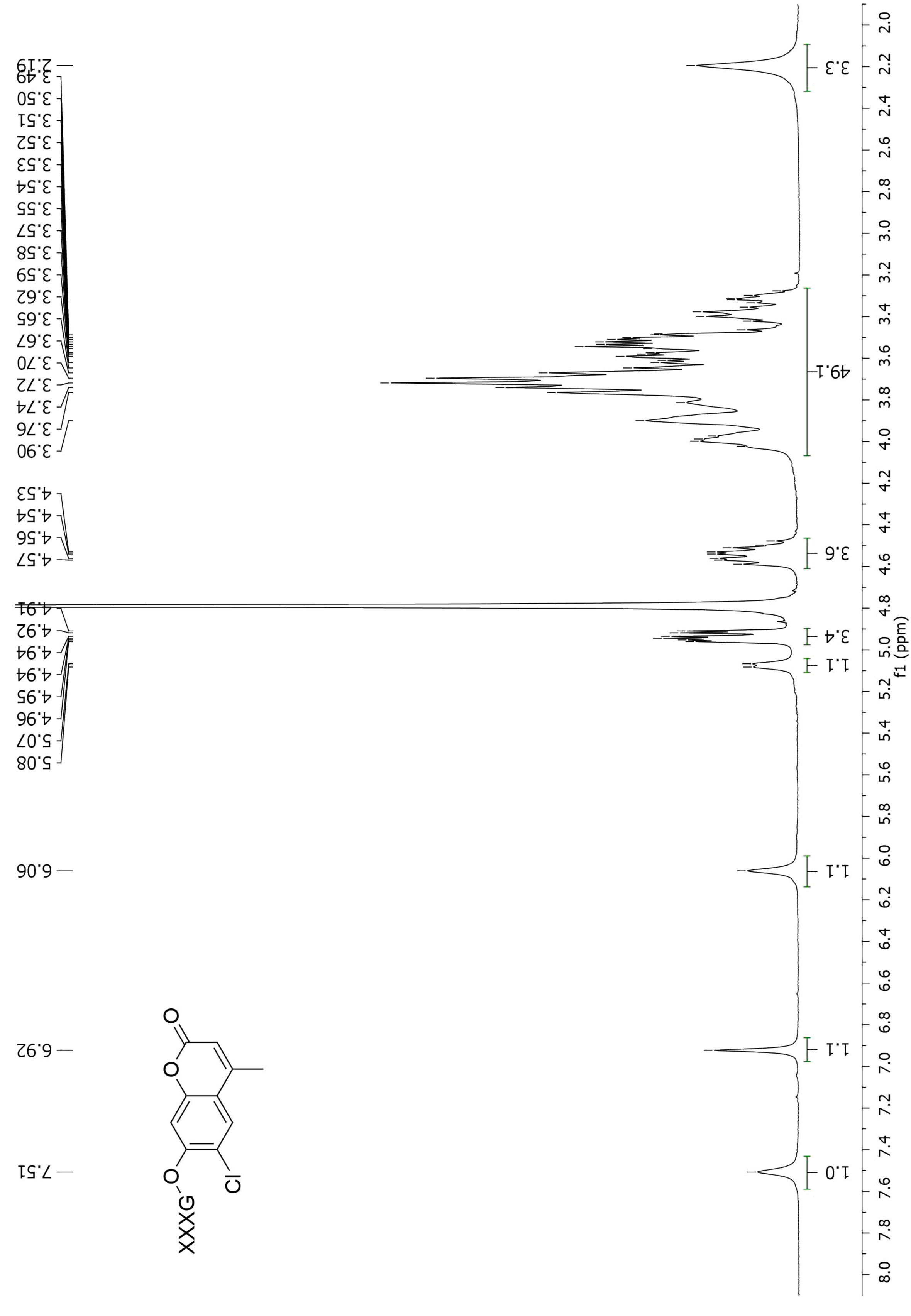

Supplemental Figures and Tables

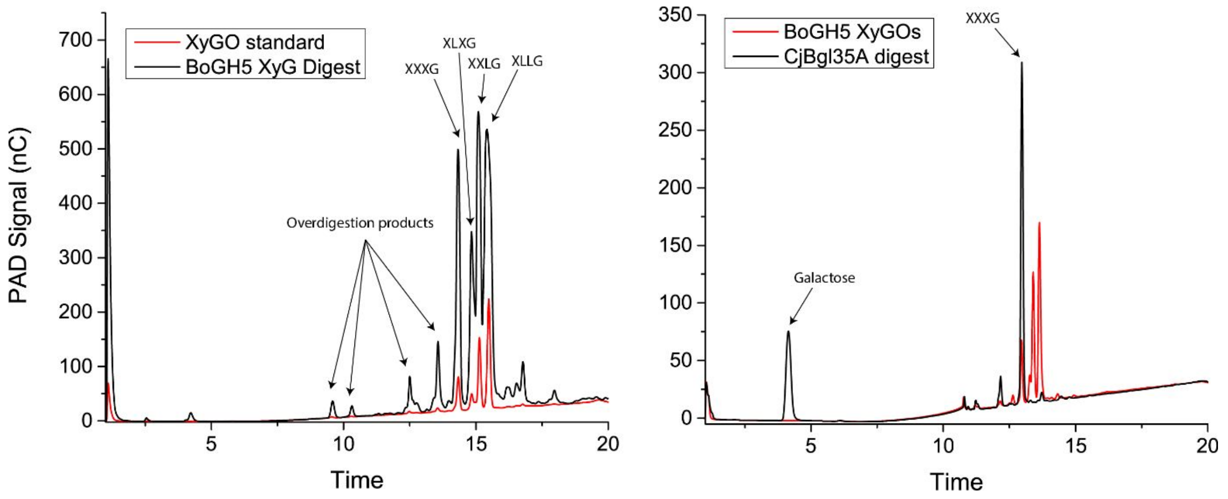

Supplemental Figure 1: HPAEC-PAD traces of xyloglucan oligosaccharides generated for the production of 4MU-XXXG and 6C4MU-XXXG. The left chromatogram shows the result of hydrolysis of xyloglucan by BoGH5 and right chromatogram shows the result of subsequent degalactosylation by CjBgl35A.

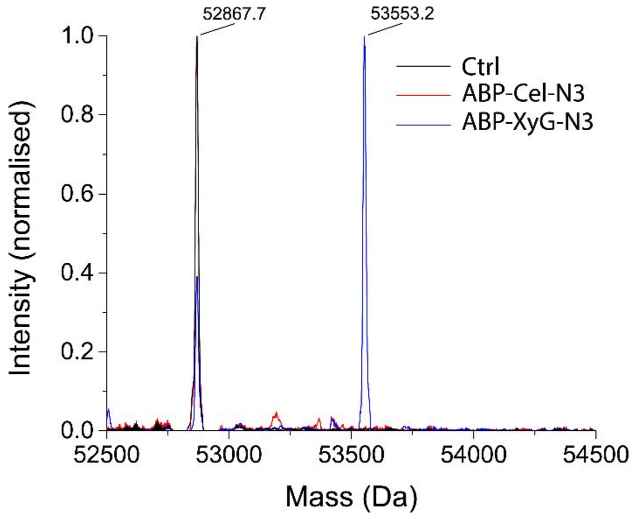

Supplemental Figure 2: Intact MS of BoGH5A xyloglucanase treated with 100 µM ABP-Cel-N3, 100 µM ABP-XyG-N3, or control for 1 hour.

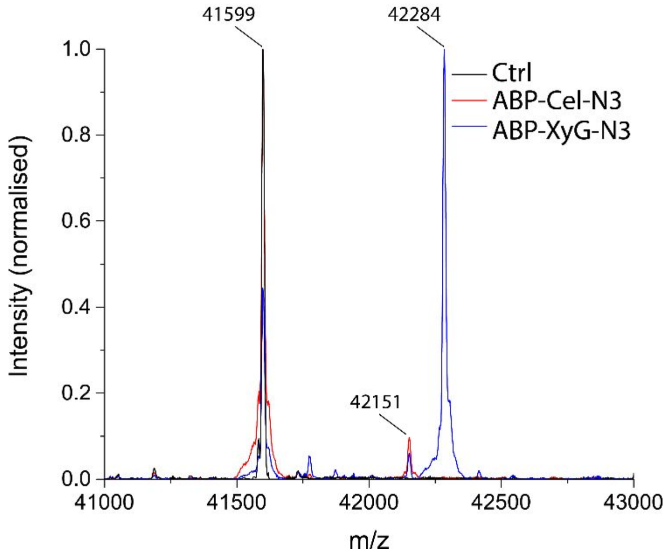

Supplemental Figure 3: Intact MS of PpXG5 xyloglucanase treated with 100 µM ABP-Cel-N3, 100 µM ABP-XyG-N3, or control for 1 hour.

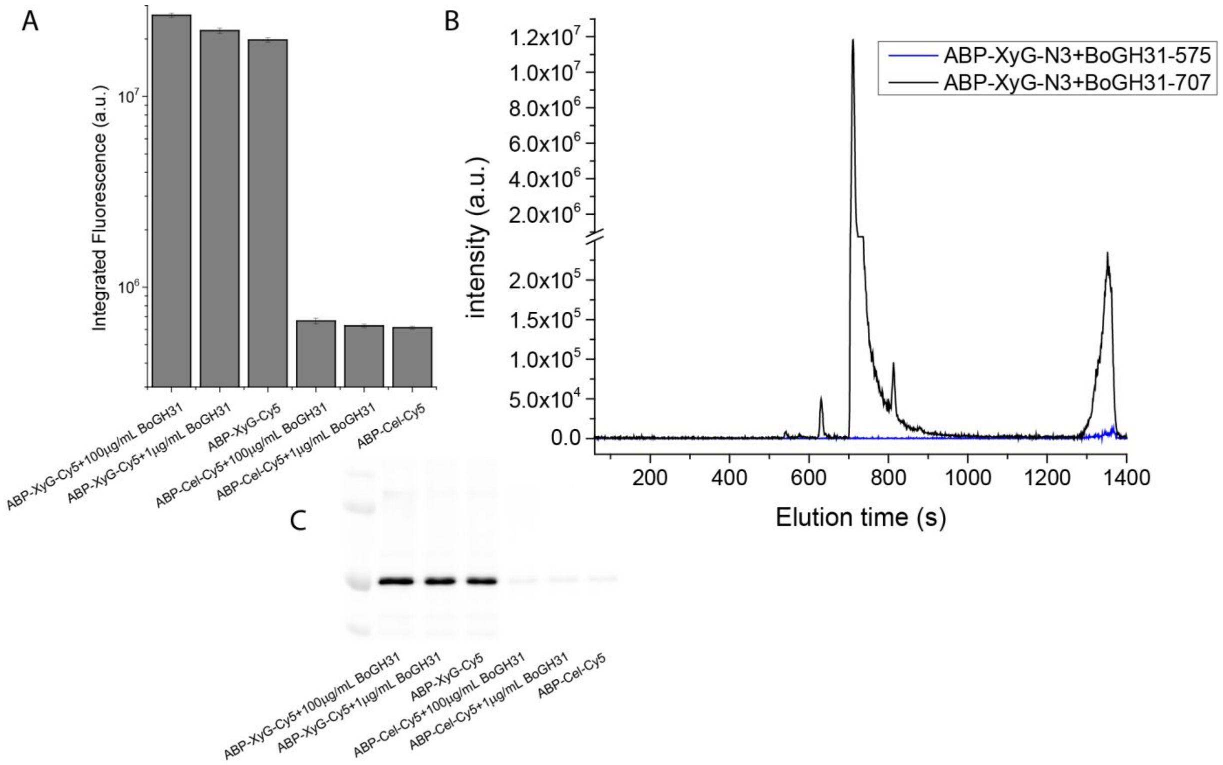

Supplemental Figure 4: Stability of ABP-XyG against enzymatic dexylosylation. A) Integrated band fluorescence in SDS-PAGE (n=3, error bars indicate standard deviation) for 10 µg/mL BoGH5A treated with ABP-XyG-Cy5 or ABP-Cel-Cy5 following 1 hour treatment of the probe with BoGH31 at 0, 1, or 100 µg/mL. B) LC-MS analysis of ABP-XyG-N3 after the same incubation with 100 µg/mL BoGH31. Extracted ion chromatograms are shown for m/z values of 707.26 (ABP-XyG-N3 sodium adduct) and 575.21 (the expected product mass following dexylosylation). C) Representative image of a gel analyzed in A.

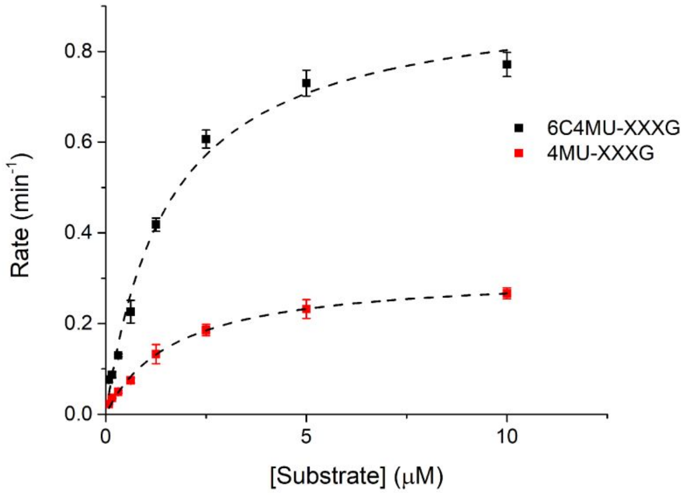

Supplemental Figure 5: Initial rate kinetics of PpXG5 acting on 4MU-XXXG and 6C4MU-XXXG. The model fit is shown as a dashed black line.

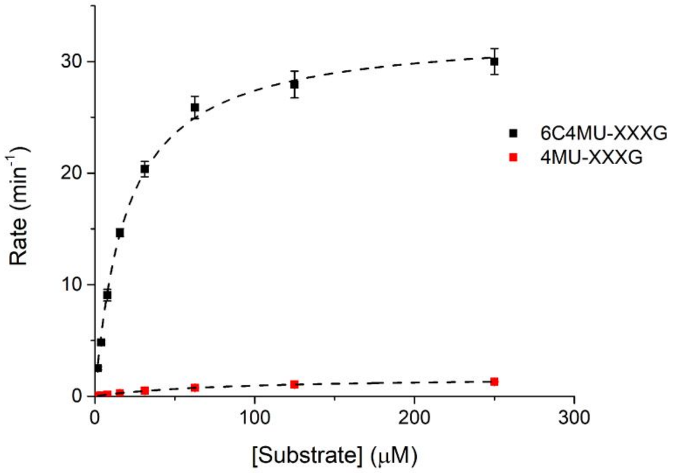

Supplemental Figure 6: Initial rate kinetics of BoGH5A acting on 4MU-XXXG and 6C4MU-XXXG. The model fit is shown as a dashed black line.

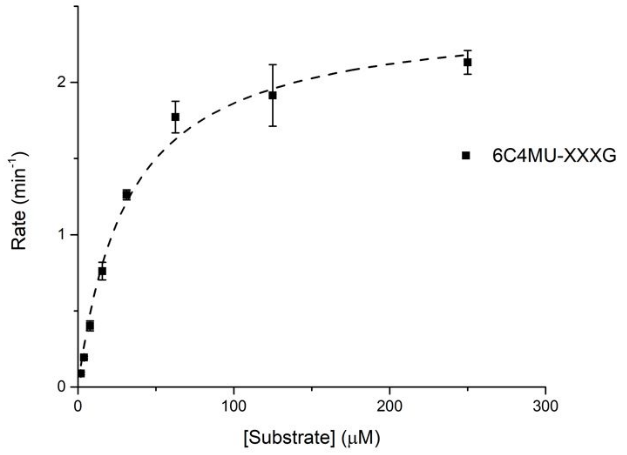

Supplemental Figure 7: Initial rate kinetics of CjCel5D acting on 6C4MU-XXXG. The model fit is shown as a dashed black line.

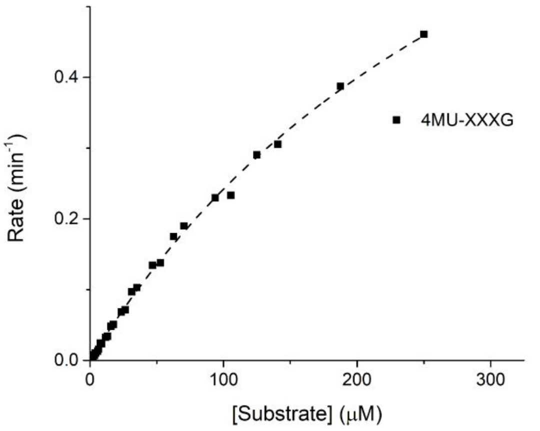

Supplemental Figure 8: Initial rate kinetics of CjCel5D acting on 4MU-XXXG. The model fit is shown as a dashed black line.

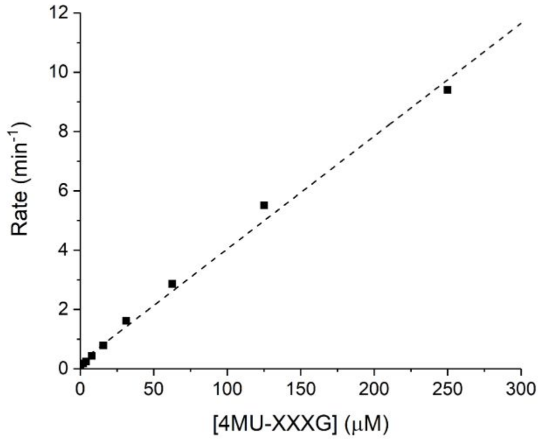

Supplemental Figure 9: Initial rate kinetics of CjCel5B acting on 4MU-XXXG. The model fit is shown as a dashed black line.

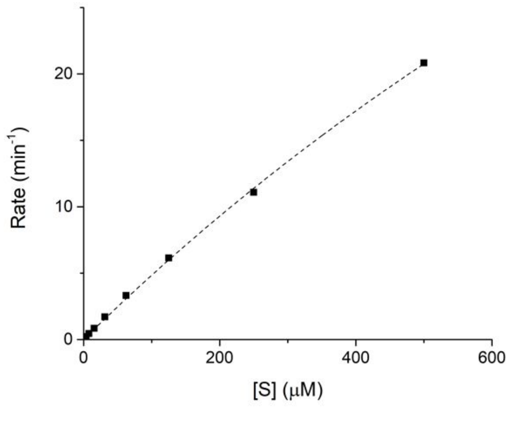

Supplemental Figure 10: Initial rate kinetics of CjCel5B acting on 4MU-GG. The model fit is shown as a dashed black line.

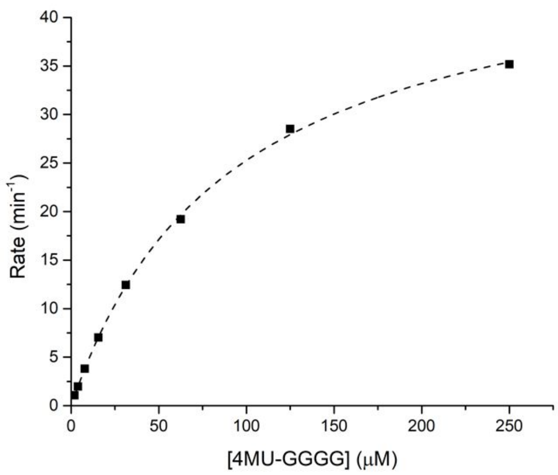

Supplemental Figure 11: Initial rate kinetics of CjCel5B acting on 4MU-GGGG. The model fit is shown as a dashed black line.

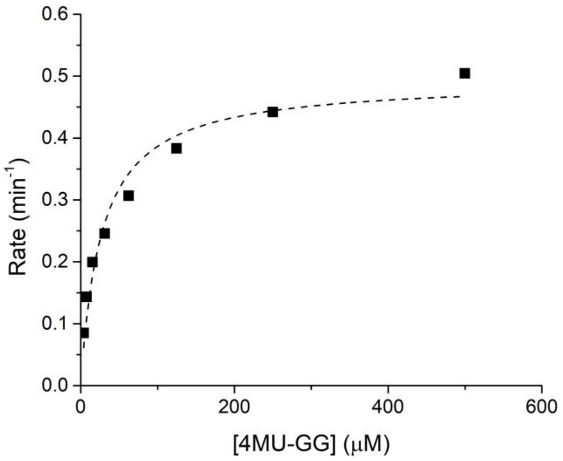

Supplemental Figure 12: Initial rate kinetics of BaCel5A acting on 4MU-GG. The model fit is shown as a dashed black line.

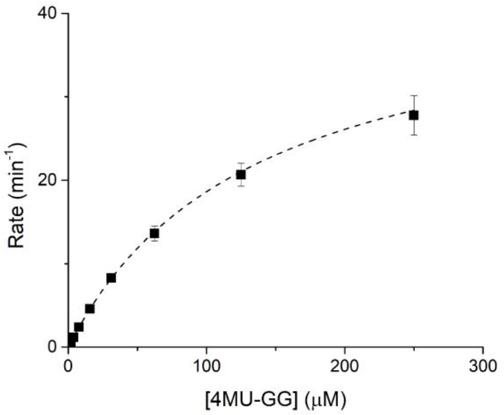

Supplemental Figure 13: Initial rate kinetics of CjCel5C acting on 4MU-GG. The model fit is shown as a dashed black line.

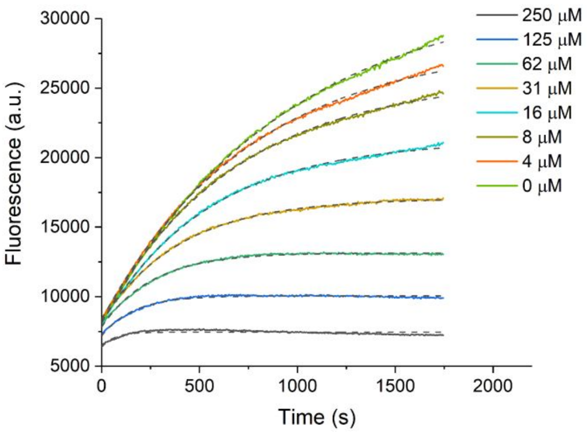

Supplemental Figure 14: Continuous inhibition kinetics of CjCel5B acting on 4MU-XXXG in the presence of different concentrations of ABP-Cel-N3. The model fits are shown as dashed black lines.

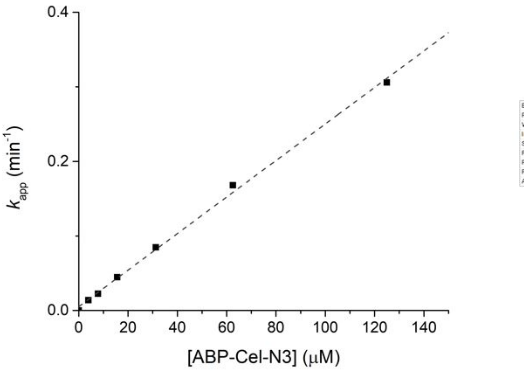

Supplemental Figure 15: k_{app} vs. inhibitor concentration for CjCel5B interacting with ABP-Cel-N3. The model fit is shown as a dashed black line.

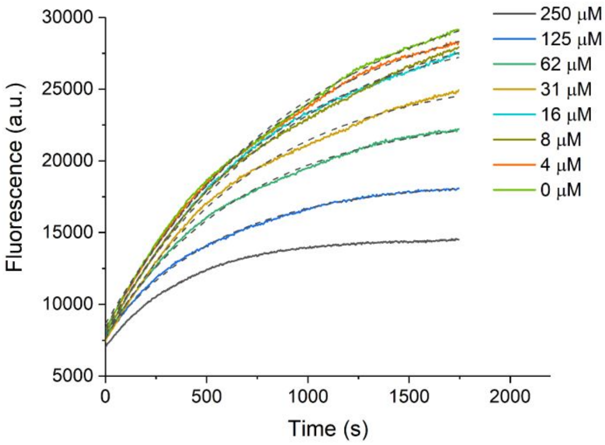

Supplemental Figure 16: Continuous inhibition kinetics of CjCel5B acting on 4MU-XXXG in the presence of different concentrations of ABP-XyG-N3. The model fits are shown as dashed black lines.

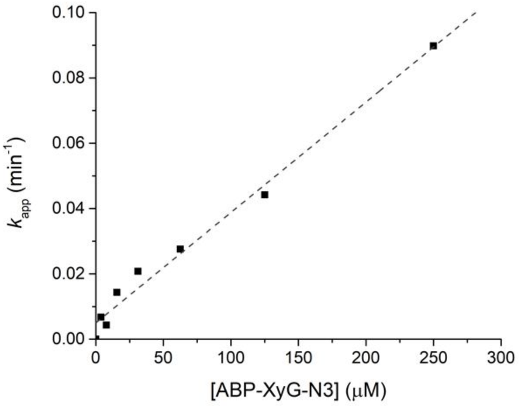

Supplemental Figure 17: k_{app} vs. inhibitor concentration for CjCel5B interacting with ABP-XyG-N3. The model fit is shown as a dashed black line.

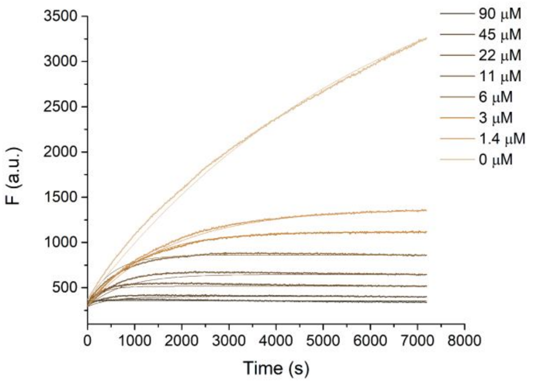

Supplemental Figure 18: Continuous inhibition kinetics of HiCel7B acting on 4MU-XXXG in the presence of different concentrations of ABP-XyG-N3. The model fits are shown as thin straight lines.

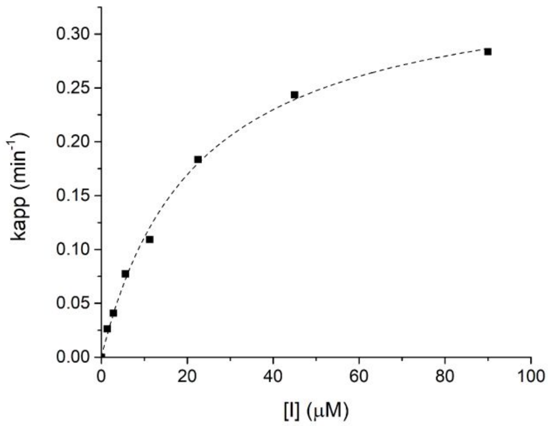

Supplemental Figure 19: k_{app} vs. inhibitor concentration for HiCel7B interacting with ABP-XyG-N3. The model fit is shown as a dashed black line.

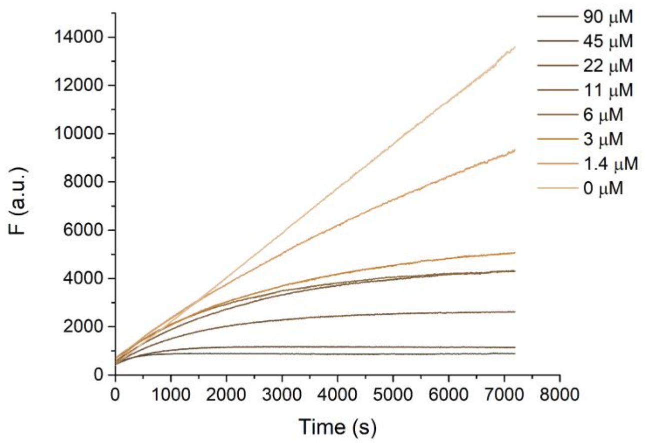

Supplemental Figure 20: Continuous inhibition kinetics of BaCel5A acting on 4MU-XXXG in the presence of different concentrations of ABP-Cel-N3. The model fits are shown as thin straight lines.

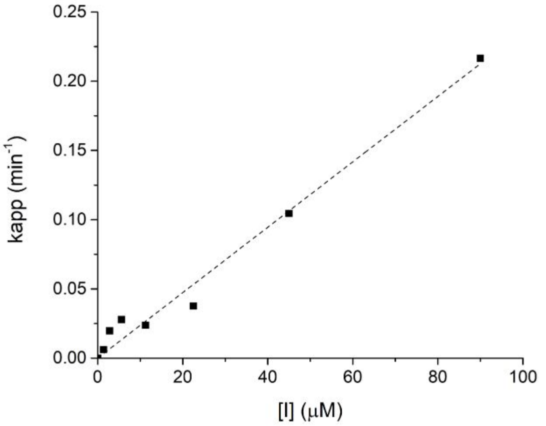

Supplemental Figure 21: k_{app} vs. inhibitor concentration for BaCel5A interacting with ABP-Cel-N3. The model fit is shown as a dashed black line.

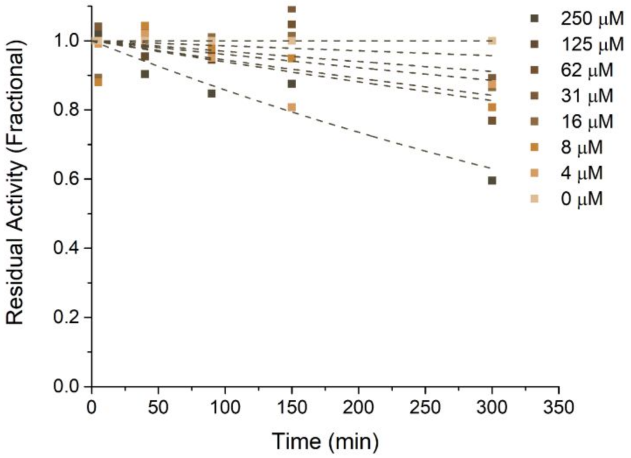

Supplemental Figure 22: Residual activity inhibition kinetics of BoGH5A inhibited by different concentrations of ABP-XyG-N3. The model fits are shown as dashed black lines. 6C4MU-XXXG was used as substrate.

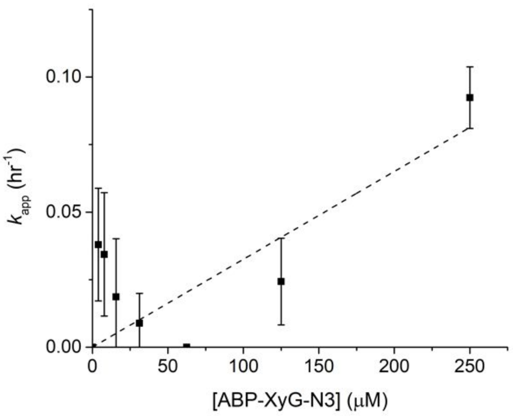

Supplemental Figure 23: k_{app} vs. inhibitor concentration for BoGH5A interacting with ABP-XyG-N3. The model fit is shown as a dashed black line.

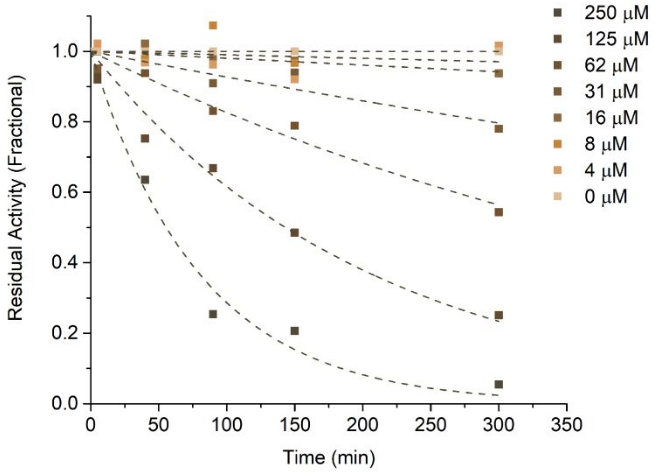

Supplemental Figure 24: Residual activity inhibition kinetics of PpXG5 inhibited by different concentrations of ABP-XyG-N3. The model fits are shown as dashed black lines. 6C4MU-XXXG was used as substrate.

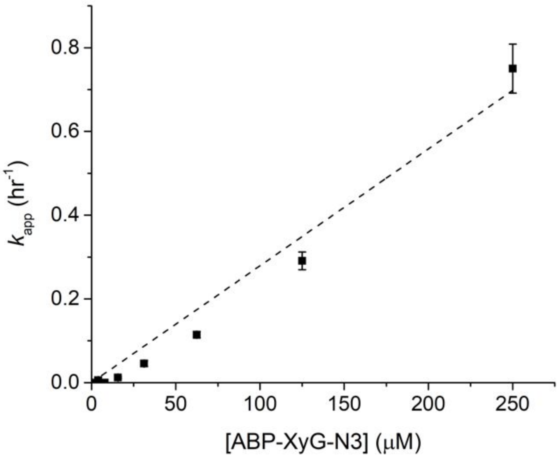

Supplemental Figure 25: k_{app} vs. inhibitor concentration for PpXG5 interacting with ABP-XyG-N3. The model fit is shown as a dashed black line.

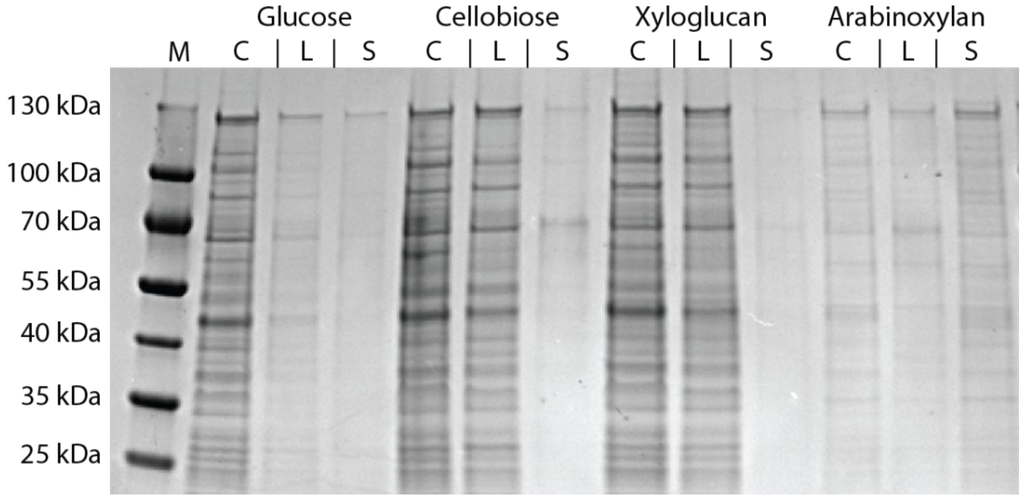

Supplemental Figure 26: Coomassie stain of the overnight *C. japonicus* cultures grown on different carbon sources (same gel as shown in figure 3A)

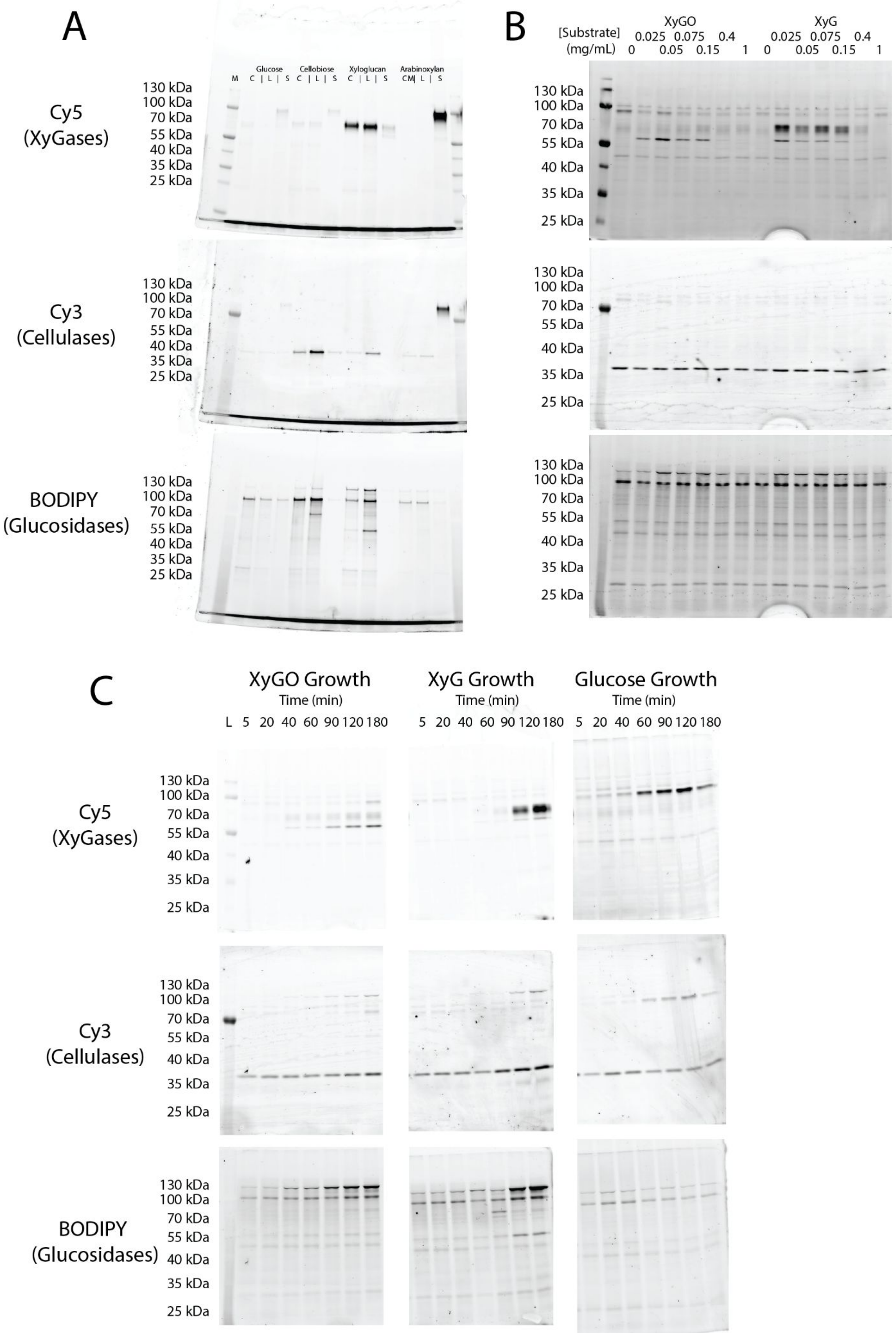
A
Cy5
(XyGases)
Cy3
(Cellulases)
BODIPY
(Glucosidases)
Glucose
Cellobiose
Xyloglucan
Arabinoxylan
M
C | L | S
130 kDa
100 kDa
70 kDa
55 kDa
40 kDa
35 kDa
25 kDa
B
[Substrate]
(mg/mL)
XyGO
XyG
0
0.025
0.05
0.075
0.15
0.4
1
C
XyGO Growth
XyG Growth
Glucose Growth
Time (min)
L
5
20
40
60
90
120
180

Supplemental Figure 26 cont'd: Greyscale fluorescent images of the overnight *C. japonicus* cultures grown on different carbon sources (lettering is the same in figure 3)

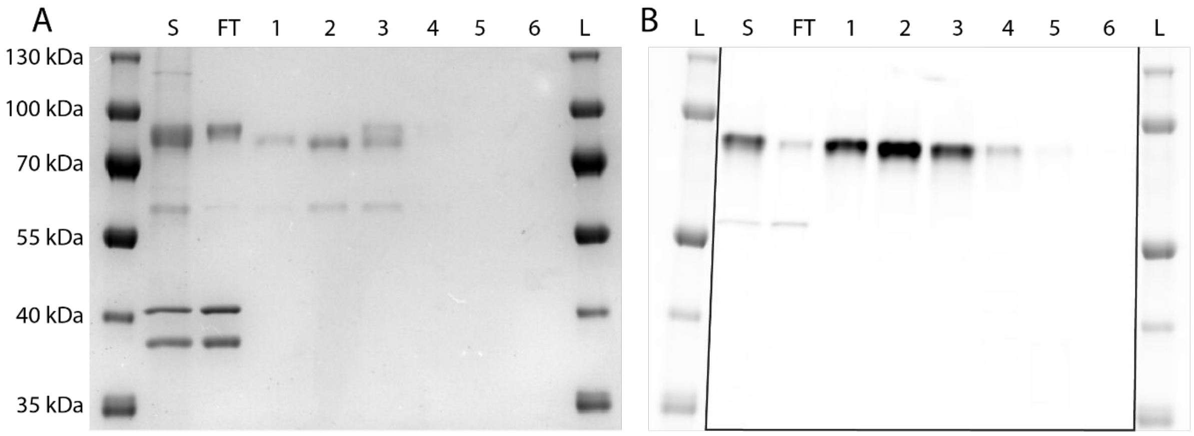

Supplemental Figure 27: SDS-PAGE analysis of the anion-exchange purification fractions from arabinoxylan-grown *C. japonicus* secretome. Panel A is Coomassie stain for total protein, panel B is Cy5 fluorescence measured after staining protein samples with ABP-XyG-Cy5. Contrast in panel B has been increased outside of the black box to enhance the visibility of the ladder bands. Above the gel images, "L" indicates ladder, "S" indicates whole secretome sample loaded onto the column, "FT" indicates flowthrough from the column, and numbers indicate the elution gradient fraction number.

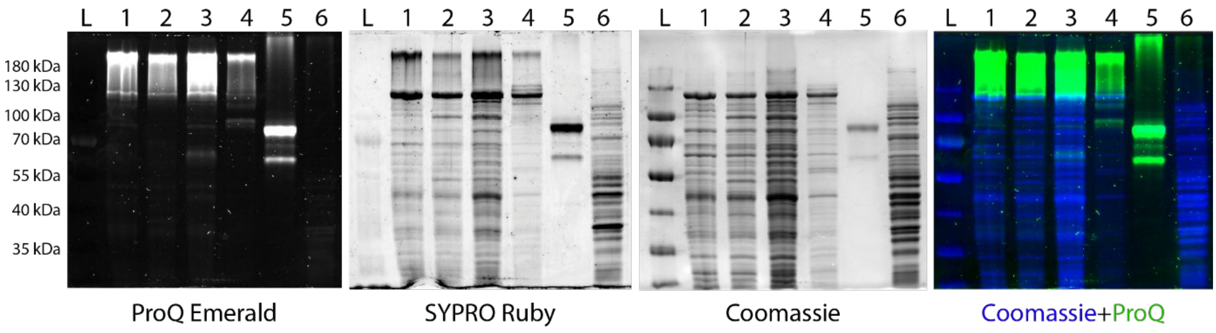

Supplemental Figure 28: Staining SDS-PAGE-separated bacterial samples for glycans. Contrast-optimised images are shown for each stain type (indicated below each panel). Lane contents are: L – Ladder, 1 – glucose-grown *C. japonicus* lysate, 2 – cellobiose-grown *C. japonicus* lysate, 3 – xyloglucan-grown *C. japonicus* lysate, 4 – arabinoxylan-grown *C. japonicus* lysate, 5 –partially purified Cel5B, 6 – *E. coli* lysate.

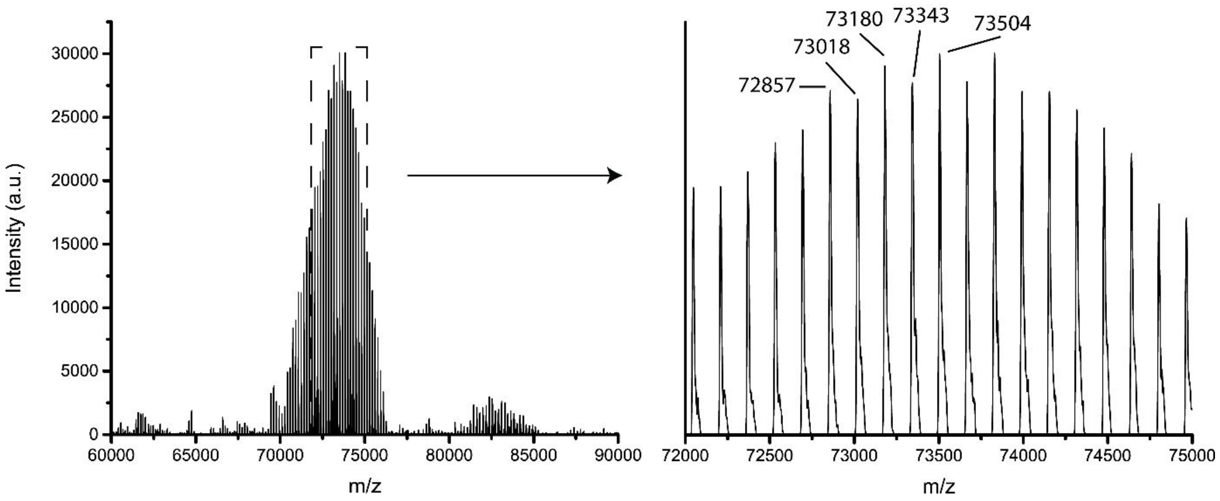

Supplemental Figure 29: Deconvoluted intact mass spectrum of partially purified native Cel5B (same sample as lane 5 in Supplemental Figure 28).

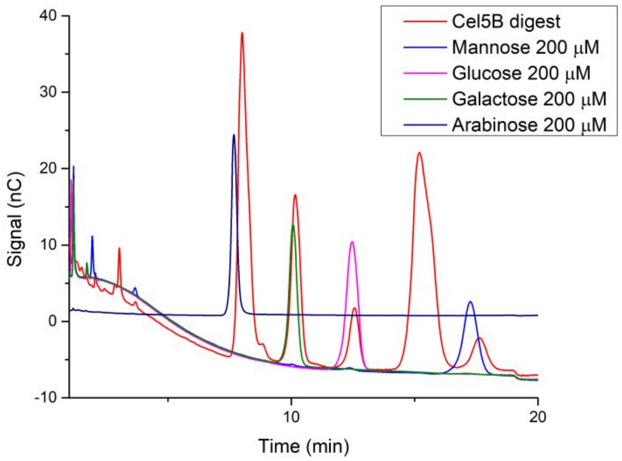

Supplemental Figure 30: HPAEC-PAD analysis of acid-hydrolyzed, partially purified native Cel5B from *C. japonicus*.

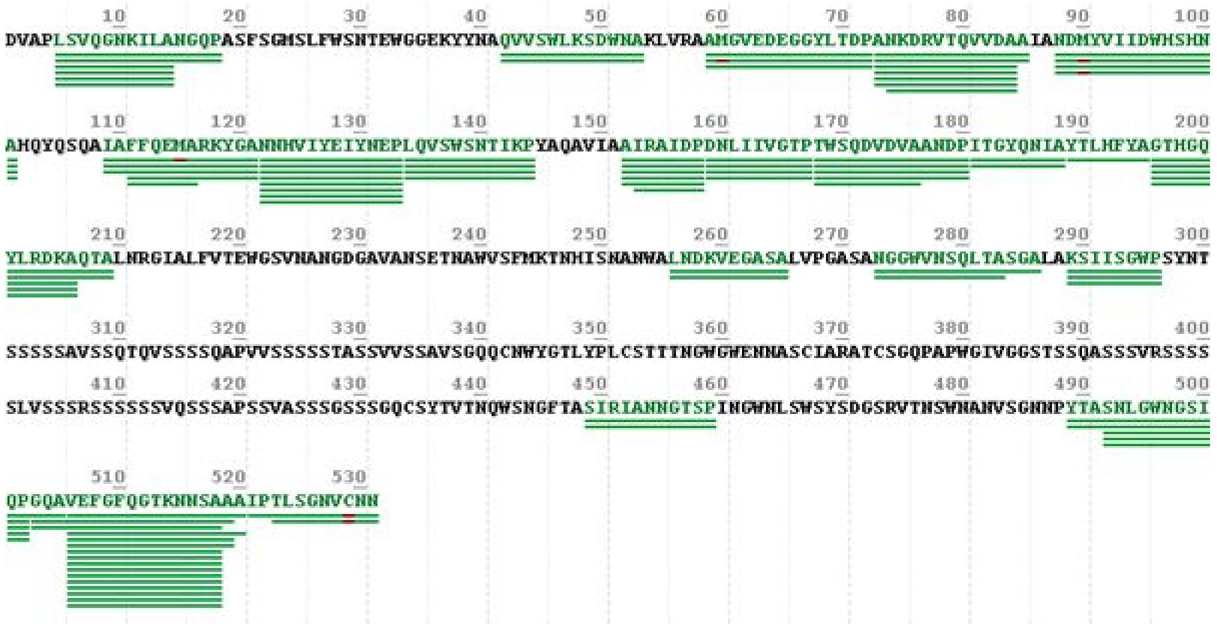

Supplemental Figure 31: Peptide coverage map for native Cel5B digested with Proalanase and analyzed by LC-MS/MS. Peptides were identified using Byonic software. Each horizontal green line represents a detected peptide that mapped onto that region of the amino acid sequence. Red segments indicate modified sites (+16 oxidation for methionine, carbamidomethylation for cysteine).

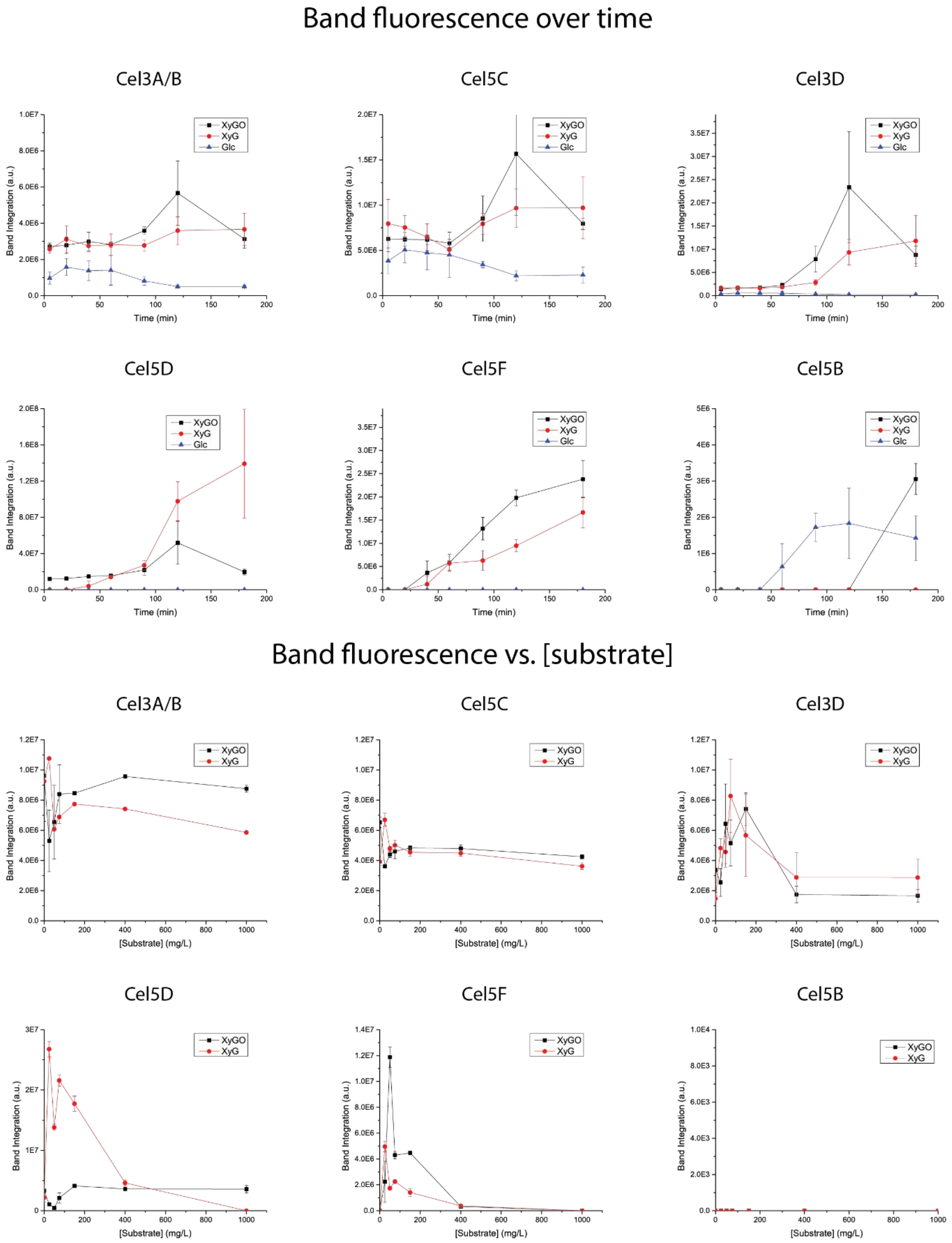

Supplemental Figure 32: Band integrations (n=3) for induction of different glycoside hydrolases by XyG, XyGOs, or glucose at different concentrations or over time.

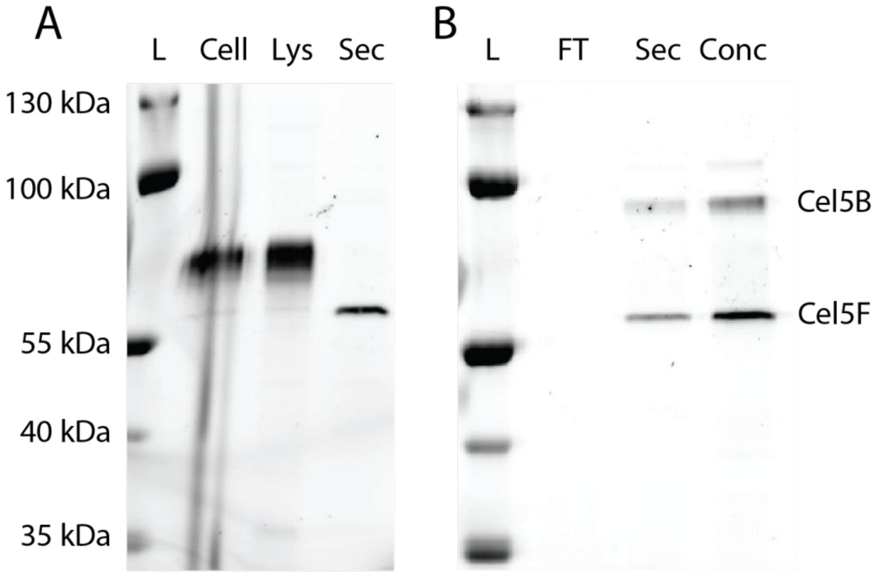

Supplemental Figure 33: Analysis of ABP-XyG-Cy5 reactive bands found in the early xyloglucan oligosaccharides -induced culture. “L” indicates ladder. A) Cy5 fluorescence SDS-PAGE of early xyloglucan oligosaccharide-induced *C. japonicus* culture. Whole cells (“cell”), lysed cells (“Lys”), or cell-free secretome (“Sec”) were stained with ABP-XyG-Cy5. B) Cy5 fluorescence SDS-PAGE of early xyloglucan oligosaccharide -induced *C. japonicus* culture supernatant. “Sec” is the secretome collected from a large-scale xyloglucan oligosaccharide early induction culture, “Conc” is the concentrated secretome used for pulldown, and “FT” is the flowthrough from the centrifugal concentrator.

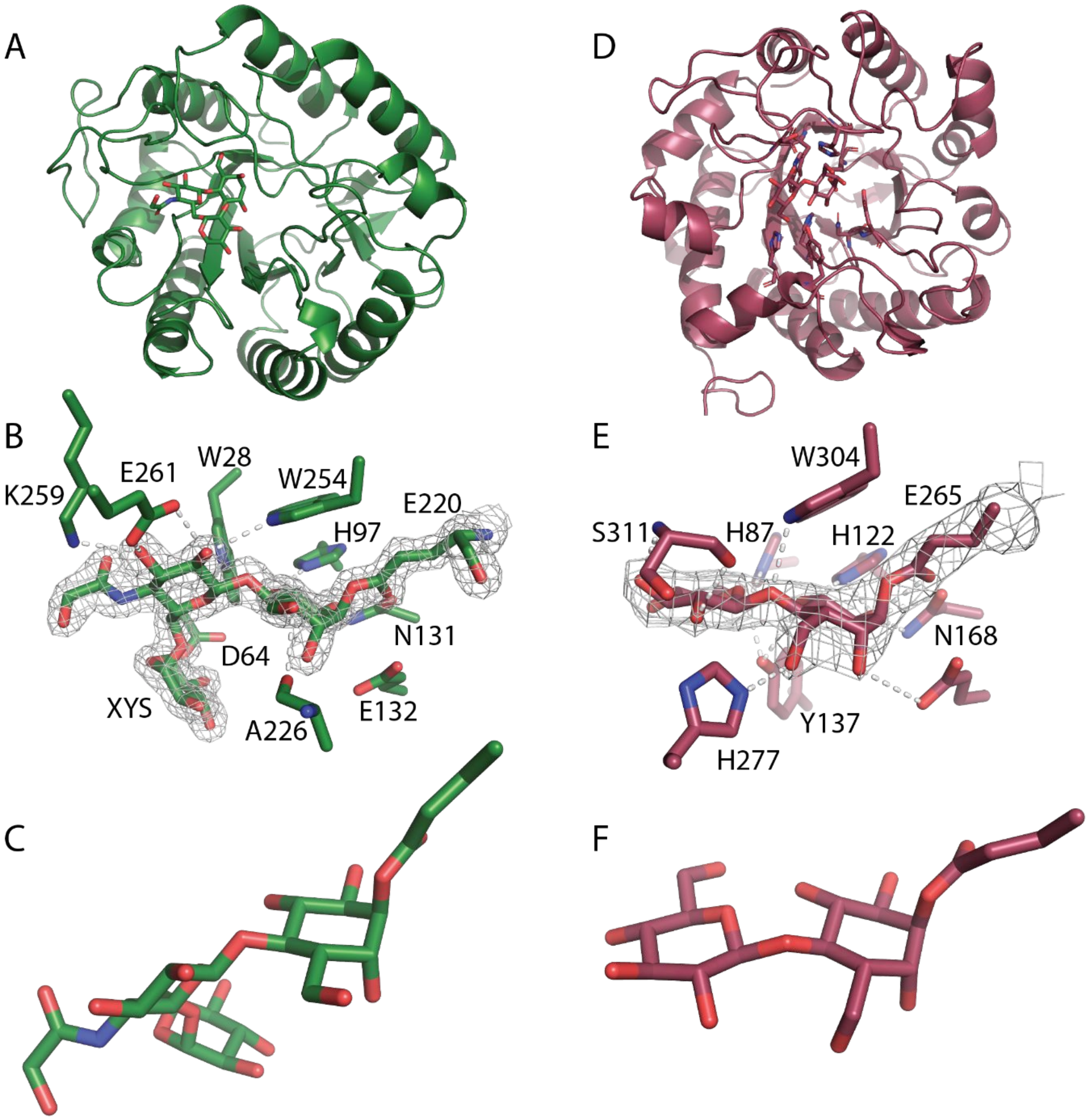

Supplemental Figure 34: Structures of CjCel5B and CjCel5C. A) Overall fold of CjCel5B. B) Active site of CjCel5B. ABP-XyG-N3 covalently linked to the catalytic nucleophile (E220) is shown surrounded by density mesh. Active site residues with hydrogen bonds (white dashed lines) to the ligand are shown as sticks. C) Isolated view of ABP-XyG-N3conformation in the active site of CjCel5B. D) Overall fold of CjCel5C. E) Active site of CjCel5C. ABP-Cel covalently linked to the catalytic nucleophile (E265) is shown surrounded by density mesh. Active site residues with hydrogen bonds (white dashed lines) to the ligand are shown as sticks. F) Isolated view of CB396 conformation in the active site of CjCel5C.

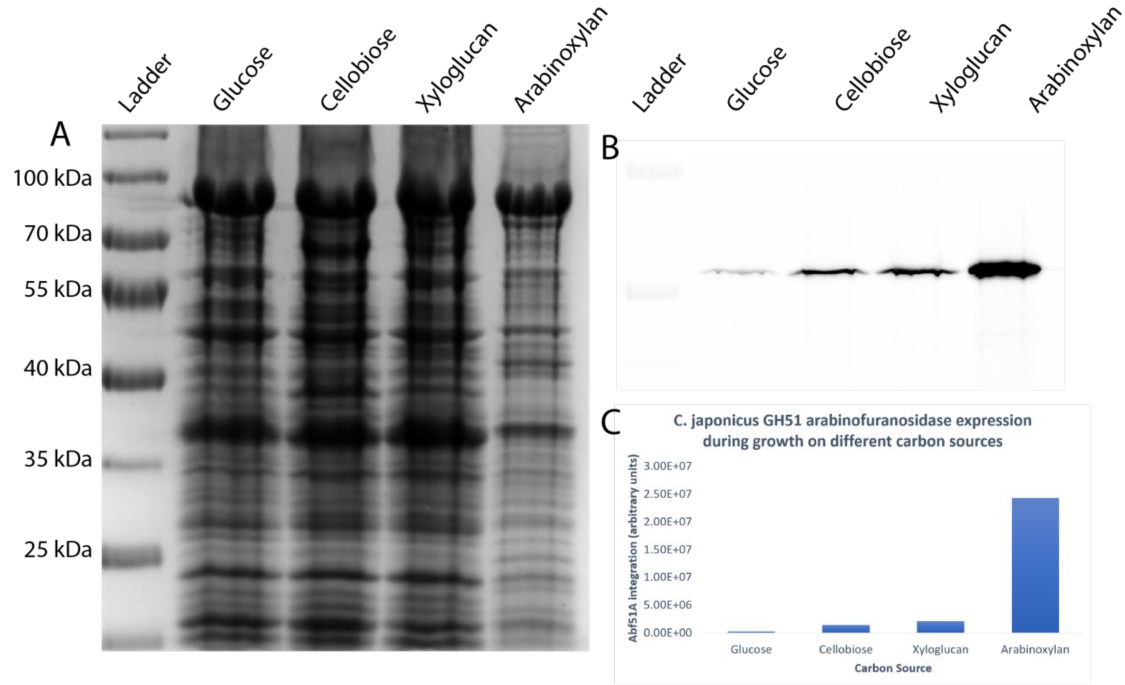

Supplemental Figure 35: ABPP of arabinofuranosidases produced by *C. japonicus* during growth on different carbon sources using ABP-αAraf-Cy5. A) Coomassie stain of protein extracted from *C. japonicus* grown on different carbon sources indicated above each lane. B) Cy5 fluorescence of the gel from panel A. C) Quantitation of the integrated fluorescence of the band observed in each lane.

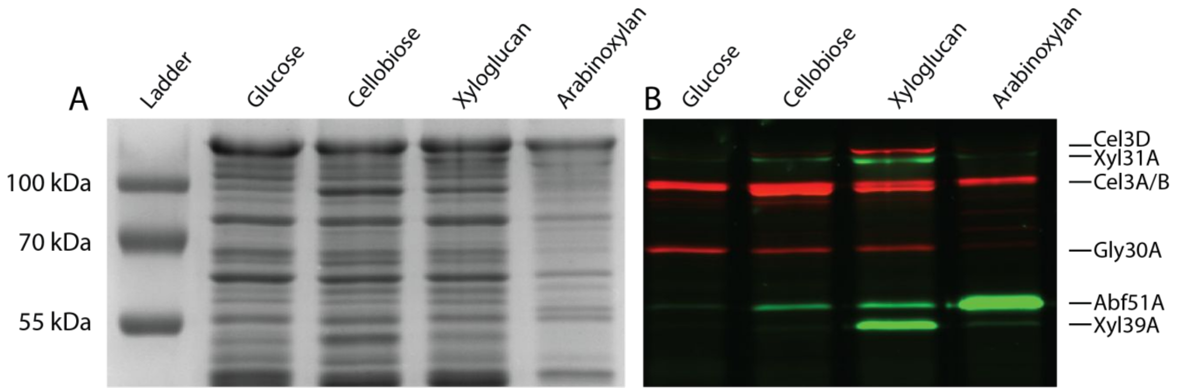

Supplemental Figure 36: Comparative staining of *C. japonicus* lysates with ABP-βXyl-BodipyFL and ABP-βGlc-Cy5. A) Coomassie stain SDS-PAGE separated *C. japonicus* lysates. The carbon source used to grow cells for each lysate is listed above each lane. B) Cy5 (red, ABP- βGlc-Cy5) and BODIPY (green, ABP- βXyl-BodipyFL) fluorescence from the gel shown in panel A. Putative assignments of band identity based on pulldown (see main text) are shown to the right of the image.

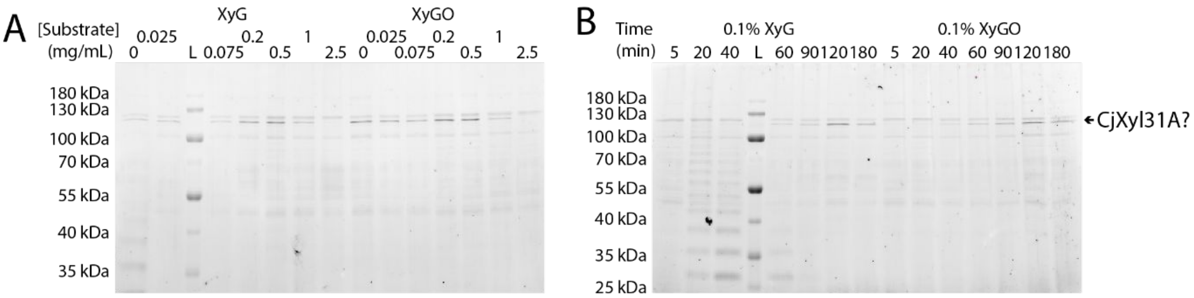

Supplemental Figure 37: Detection of α-xylosidases in induced *C. japonicus* lysates using ABP-αXyl-Cy5. A) Cy5 fluorescence of *C. japonicus* lysates from cultures induced with different concentrations of xyloglucan or xyloglucan oligosaccharides for 3 hours at 30°C. "L" indicate ladder. B) Cy5 fluorescence of *C. japonicus* lysates from cultures induced with 0.1% xyloglucan or xyloglucan oligosaccharides for variable times at 30°C. The putative CjXyl31A band is labelled.

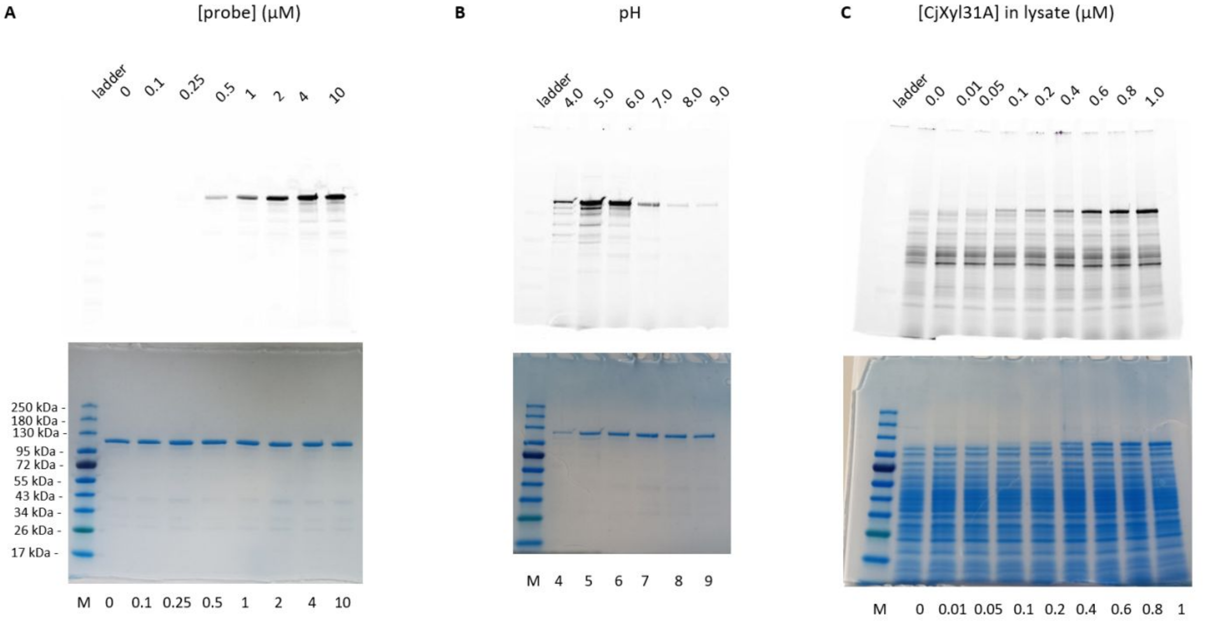

Supplemental Figure 38: (A–C) Probe concentration -, pH - and protein concentration - dependence in *E. coli* doped lysate of *Cj*Xyl31A labelling with ABP-αXyl-Cy5

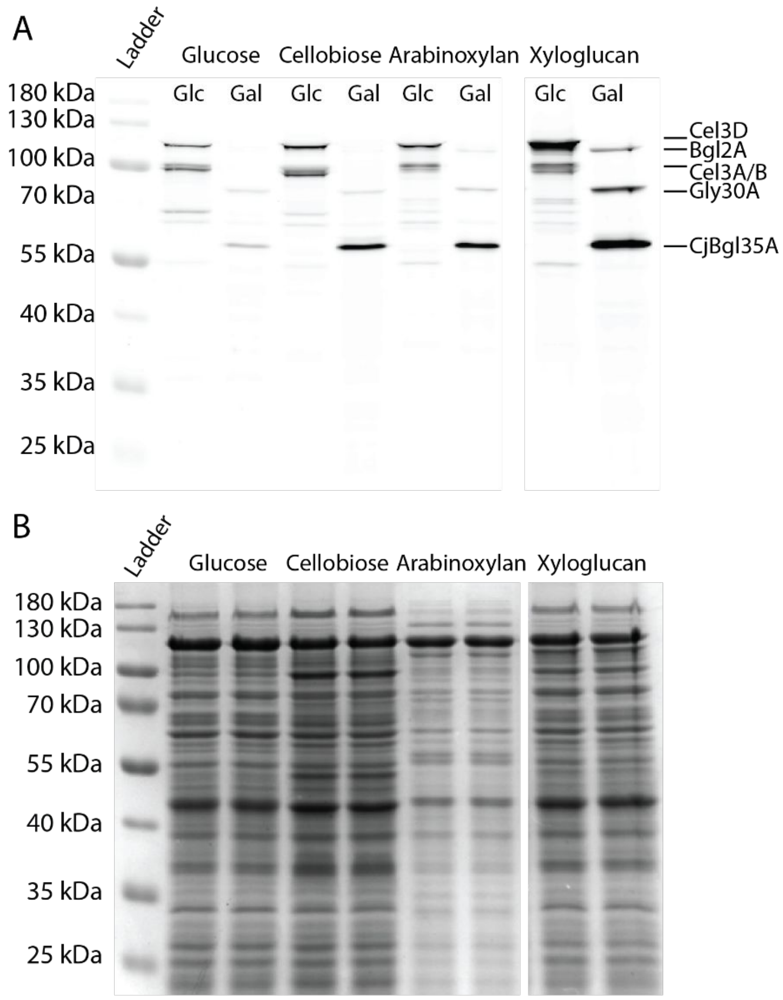

Supplemental Figure 39: Comparative staining of *C. japonicus* lysates with ABP-βGal-Cy5 and ABP-βGlc-Cy5. A) Cy5 fluorescence image following SDS-PAGE separation of lysate proteins following growth on different substrates labelled above each pair of lanes. "Glc" indicates staining with ABP-βGlc-Cy5 and "Gal" indicates staining with ABP-βGal-Cy5. Putative assignments of band identity based on pulldown (see main text) are shown to the right of the image. B) Coomassie stain of the SDS-PAGE gel shown in panel A.

Supplemental Table 1: Data collection and refinement statistics (molecular replacement)

	*Cj*Cel5D	CjCel5B	CjCel5B	CjCel5C	CjCel5C
	Complex with ABP-XyG-N3 (PDB 8OZ1)	Unliganded (PDB 8BQC)	Complex with ABP-XyG-N3 (PDB 8BQA)	Unliganded (PDB 8BN7)	Complex with ABP-Cel (PDB 8BQB)
**Data collection**					
Space group	P 1 2_1 1	P 1 2_1 1	P 1 2_1 1	C 2 2 2_1	C 2 2 2_1
a, *b*, *c* (Å)	52.4, 57.1, 65.9	45.3, 53.5, 63.2	45.5 51.8 64.6	126.3, 175.7, 115.3	126.4, 176.2, 115.7
α, β, γ (°)	90, 98.3, 90	90.0, 109.7, 90.0	90.0 110.4 90.0	90.0, 90.0, 90.0	90.0, 90.0, 90.0
Resolution (Å)	51.78-1.30 (1.32-1.30)	42.09-1.57 (1.60-1.57)	32.92-1.67 (1.70-1.67)	102.6-2.18 (2.22-2.18)	102.9-2.70 (2.75-2.70)
R_{meas}	0.156 (1.246)	0.160 (2.910)	0.198 (2.784)	0.274 (3.926)	0.369 (3.131)
R_{pim}	0.044 (0.542)	0.062 (1.093)	0.108 (1.442)	0.074 (1.044)	0.108 (0.948)
$I / \sigma I$	8.3 (0.9)	7.7 (0.7)	6.6 (0.8)	8.3 (0.9)	6.8 (0.9)
Completeness (%)	97.2 (75.3)	88.5 (100.0)	97.5 (96.7)	91.0 (99.9)	93.8 (99.0)
Redundancy	11.3 (5.0)	6.5 (6.9)	6.1 (6.3)	13.5 (13.9)	11.6 (10.6)
$CC_{1/2}$	0.997 (0.577)	0.998 (0.319)	0.991 (0.322)	0.996 (0.351)	0.989 (0.377)
**Refinement**					
No. reflections	91883 (3497)	35344 (1931)	32027 (1611)	60843 (3330)	33580 (1744)
R_{work} / R_{free}	0.13/0.17 (0.26/0.28)	0.18/0.23 (0.33/0.35)	0.19/0.23 (0.35/0.36)	0.19/0.23 (0.32/0.33)	0.18/0.22 (0.32/0.38)
No. atoms					
Protein	2996	2327	2293	8146	8043
Ligand/ion	76	0	36	0	69
Water	430	248	197	183	22
B-factors					
Protein	18.3	20.0	25.2	41.9	49.4
Ligand/ion	22.0	-	24.8	-	39.9
Water	29.1	26.6	30.9	33.2	32.7
R.m.s. deviations					
Bond lengths (Å)	0.016	0.008	0.009	0.009	0.006
Bond angles (°)	1.90	1.48	1.51	1.63	1.32

*Values in parentheses are for highest-resolution shell.

Supplemental Table 2: Kinetic parameters for the hydrolysis of 4MU-XXXG and 4MU-GGGG by different *endo*-β-glucanases.

**Enzyme**	**Substrate**	**K_M (μM)**	**k_{cat} (min^{-1})**	**K_{cat}/K_M ($M^{-1}s^{-1}$)**
**PpXG5**	4MU-XXXG	1.7+/-0.1	0.31+/-0.01	3000
	6C4MU-XXXG	1.6+/-0.1	0.93+/-0.04	9700
**BoGH5**	4MU-XXXG	81+/-3	1.73+/-0.02	360
	6C4MU-XXXG	20+/-1	32.7+/-0.5	27000
**CjCel5D**	4MU-XXXG	370+/-50	1.1+/-0.2	51
	6C4MU-XXXG	32+/-4	2.5+/-0.1	1300
**BaCel5A**	4MU-GG	28+/-3	0.49+/-0.03	300
**HiCel7B**	4MU-GG	76+/-7	106+/-5	23000[21]
**CjCel5B**	4MU-XXXG	>500	>20	630+/-30
	4MU-GGGG	91+/-4	48+/-2	8800
	4MU-GG	>500	>25	840+/-40
**CjCel5C**	4MU-GG	130+/-10	44+/-2	5600

Supplemental Table 3: Specific activity (1 mg/mL substrate, 37 °C) for the hydrolysis of polysaccharides by CjCel5B and CjCel5C. CMC = carboxymethylcellulose, kGM = konjac glucomannan, bMLG = barley mixed-linkage glucan, cGM = carob galactomannan, tXyG = tamarind xyloglucan. 1 U = 1 micromole of reducing ends formed per minute.

Enzyme	Substrate	Specific activity (U/mg)
**CjCel5B**	CMC	33+/-3
	kGM	4.2+/-0.6
	MLG	47+/-3
	cGM	0.006+/-0.002
	tXyG	0.011+/-0.002
**CjCel5C**	CMC	11.3+/-0.6
	kGM	17+/-3
	MLG	8.6+/-0.6
	cGM	0.023+/-0.002
	tXyG	<0.005

Supplemental Table 4: Specific activity (0.05 mM substrate, 25 °C, pH 7.5 NaPi buffer) for the hydrolysis of 4-methylumbelliferyl monosaccharide substrates by CjXyl39A. 1 U = 1 micromole of free 4MU formed per minute.

Substrate	Specific activity (mU/mg)
4MU-bGlc	0.52+/-0.03
4MU-bGal	1.01+/-0.05
4MU-aAraf	0.014+/-0.002
4MU-bMan	0.0007+/-0.0001
4MU-bXyl	144+/-7

Supplemental References

(1) Attia, M. A.; Nelson, C. E.; Offen, W. A.; Jain, N.; Davies, G. J.; Gardner, J. G.; Brumer, H. In Vitro and in Vivo Characterization of Three *Cellvibrio Japonicus* Glycoside Hydrolase Family 5 Members Reveals Potent Xyloglucan Backbone-Cleaving Functions. *Biotechnol Biofuels* **2018**, *11* (1), 45.

(2) Larsbrink, J.; Izumi, A.; Ibatullin, F. M.; Nakhai, A.; Gilbert, H. J.; Davies, G. J.; Brumer, H. Structural and Enzymatic Characterization of a Glycoside Hydrolase Family 31 α-Xylosidase from *Cellvibrio Japonicus* Involved in Xyloglucan Saccharification. *Biochemical Journal* **2011**, *436* (3), 567–580.

(3) Larsbrink, J.; Rogers, T. E.; Hemsworth, G. R.; McKee, L. S.; Tauzin, A. S.; Spadiut, O.; Klinter, S.; Pudlo, N. A.; Urs, K.; Koropatkin, N. M.; et al. A Discrete Genetic Locus Confers Xyloglucan Metabolism in Select Human Gut Bacteroidetes. *Nature* **2014**, *506* (7489), 498–502.

(4) Gloster, T. M.; Ibatullin, F. M.; Macauley, K.; Eklöf, J. M.; Roberts, S.; Turkenburg, J. P.; Bjørnvad, M. E.; Jørgensen, P. L.; Danielsen, S.; Johansen, K. S.; et al. Characterization and Three-Dimensional Structures of Two Distinct Bacterial Xyloglucanases from Families GH5 and GH12. *Journal of Biological Chemistry* **2007**, *282* (26), 19177–19189.

(5) Davies, G.; Tolley, S.; Wilson, K.; Schülein, M.; Wöldike, H. F.; Dodson, G. Crystallization and Preliminary X-Ray Analysis of a Fungal Endoglucanase I. *J Mol Biol* **1992**, *228* (3), 970–972.

(6) Schülein, M. Enzymatic Properties of Cellulases from Humicola Insolens. *J Biotechnol* **1997**, *57* (1–3), 71–81.

(7) Davies, G. J.; Dauter, M.; Brzozowski, A. M.; Bjørnvad, M. E.; Andersen, K. v.; Schülein, M. Structure of the Bacillus Agaradherans Family 5 Endoglucanase at 1.6 A and Its Cellobiose Complex at 2.0 A Resolution. *Biochemistry* **1998**, *37* (7), 1926–1932.

(8) Almagro Armenteros, J. J.; Tsirigos, K. D.; Sønderby, C. K.; Petersen, T. N.; Winther, O.; Brunak, S.; von Heijne, G.; Nielsen, H. SignalP 5.0 Improves Signal Peptide Predictions Using Deep Neural Networks. *Nat Biotechnol* **2019**, *37* (4), 420–423.

(9) Studier, F. W. Protein Production by Auto-Induction in High Density Shaking Cultures. *Protein Expr Purif* **2005**, *41* (1), 207–234.

(10) Winter, G.; Lobley, C. M. C.; Prince, S. M. Decision Making in Xia2. *Acta Crystallogr D Biol Crystallogr* **2013**, *69* (7), 1260–1273.

(11) Winter, G.; Waterman, D. G.; Parkhurst, J. M.; Brewster, A. S.; Gildea, R. J.; Gerstel, M.; Fuentes-Montero, L.; Vollmar, M.; Michels-Clark, T.; Young, I. D.; et al. DIALS: Implementation and Evaluation of a New Integration Package. *Acta Crystallogr D Struct Biol* **2018**, *74*, 85–97.

(12) Ballard, C.; Keegan, R.; Krissinel, E.; Lebedev, A.; Uski, V.; Waterman, D.; Wojdyr, M. CCP4: A Resource for Macromolecular Crystallography. *Acta Crystallogr A Found Adv* **2014**, *70* (a1), C1723–C1723.

(13) Vagin, A.; Teplyakov, A. Molecular Replacement with MOLREP. *Acta Crystallogr D Biol Crystallogr* **2010**, *66* (1), 22–25.

(14) Emsley, P.; Lohkamp, B.; Scott, W. G.; Cowtan, K. Features and Development of Coot. *Acta Crystallogr D Biol Crystallogr* **2010**, *66* (4), 486–501.

(15) Murshudov, G. N.; Skubák, P.; Lebedev, A. A.; Pannu, N. S.; Steiner, R. A.; Nicholls, R. A.; Winn, M. D.; Long, F.; Vagin, A. A. REFMAC5 for the Refinement of Macromolecular Crystal Structures. *Acta Crystallogr D Biol Crystallogr* **2011**, *67* (4), 355–367.

(16) McGregor, N. G. S.; Artola, M.; Nin-Hill, A.; Linzel, D.; Haon, M.; Reijngoud, J.; Ram, A.; Rosso, M. N.; Van Der Marel, G. A.; Codeé, J. D. C.; et al. Rational Design of Mechanism-Based Inhibitors and Activity-Based Probes for the Identification of Retaining α-l-Arabinofuranosidases. *J Am Chem Soc* **2020**, *142* (10), 4648–4662.

(17) McGregor, N. G. S.; Coines, J.; Borlandelli, V.; Amaki, S.; Artola, M.; Nin-Hill, A.; Linzel, D.; Yamada, C.; Arakawa, T.; Ishiwata, A.; et al. Cysteine Nucleophiles in Glycosidase Catalysis: Application of a Covalent β-l-Arabinofuranosidase Inhibitor. *Angewandte Chemie - International Edition* **2021**, *60* (11), 5754–5758.

(18) Gardner, J. G.; Keating, D. H. Genetic and Functional Genomic Approaches for the Study of Plant Cell Wall Degradation in *Cellvibrio Japonicus*. In *Methods in Enzymology*; Academic Press Inc., 2012; Vol. 510, pp 331–347.

(19) Schröder, S. P.; De Boer, C.; McGregor, N. G. S.; Rowland, R. J.; Moroz, O.; Blagova, E.; Reijngoud, J.; Arentshorst, M.; Osborn, D.; Morant, M. D.; et al. Dynamic and Functional Profiling of Xylan-Degrading Enzymes in Aspergillus Secretomes Using Activity-Based Probes. *ACS Cent Sci* **2019**, *5* (6), 1067–1078.

(20) Schröder, S. P.; Petracca, R.; Minnee, H.; Artola, M.; Aerts, J. M. F. G.; Codée, J. D. C.; van der Marel, G. A.; Overkleeft, H. S. A Divergent Synthesis of L-Arabino- and d-Xylo-Configured Cyclophellitol Epoxides and Aziridines. *European J Org Chem* **2016**, *2016* (28), 4787–4794.

(21) de Boer, C.; McGregor, N. G. S.; Peterse, E.; Schröder, S. P.; Florea, B. I.; Jiang, J.; Reijngoud, J.; Ram, A. F. J.; van Wezel, G. P.; van der Marel, G. A.; et al. Glycosylated Cyclophellitol-Derived Activity-Based Probes and Inhibitors for Cellulases. *RSC Chem Biol* **2020**, *1* (3), 148–155.

(22) Ueno, Y.; Jose, J.; Loudet, A.; Pérez-Bolívar, C.; Anzenbacher, P.; Burgess, K. Encapsulated Energy-Transfer Cassettes with Extremely Well Resolved Fluorescent Outputs. *J Am Chem Soc* **2011**, *133* (1), 51–55.

(23) Lucero, C. G.; Woerpel, K. A. Stereoselective C-Glycosylation Reactions of Pyranoses: The Conformational Preference and Reactions of the Mannosyl Cation. *Journal of Organic Chemistry* **2006**, *71* (7), 2641–2647.

(24) McGregor, N.; Morar, M.; Fenger, T. H.; Stogios, P.; Lenfant, N.; Yin, V.; Xu, X.; Evdokimova, E.; Cui, H.; Henrissat, B.; et al. Structure-Function Analysis of a Mixed-Linkage β-Glucanase/Xyloglucanase from the Key Ruminal Bacteroidetes *Prevotella Bryantii* B14. *Journal of Biological Chemistry* **2016**, *291* (3), 1175–1197.

(25) Larsbrink, J.; Thompson, A. J.; Lundqvist, M.; Gardner, J. G.; Davies, G. J.; Brumer, H. A Complex Gene Locus Enables Xyloglucan Utilization in the Model Saprophyte *Cellvibrio Japonicus*. *Mol Microbiol* **2014**.

(26) Ibatullin, F. M.; Baumann, M. J.; Greffe, L.; Brumer, H. Kinetic Analyses of Retaining Endo-(Xylo)Glucanases from Plant and Microbial Sources Using New Chromogenic Xylogluco-Oligosaccharide Aryl Glycosides. *Biochemistry* **2008**, *47* (29), 7762–7769.

(27) Greffe, L.; Bessueille, L.; Bulone, V.; Bruiner, H. Synthesis, Preliminary Characterization, and Application of Novel Surfactants from Highly Branched Xyloglucan Oligosaccharides. *Glycobiology* **2005**, *15* (4), 437–445.

(28) Chilvers, K. F.; Perry, J. D.; James, A. L.; Reed, R. H. Synthesis and Evaluation of Novel Fluorogenic Substrates for the Detection of Bacterial β-Galactosidase. *J Appl Microbiol* **2001**, *91* (6), 1118–1130.

(29) Rasool, S.; Aziz-ur-Rehman; Abbasi, M. A.; Siddiqui, S. Z.; Shah, S. A. A.; Hassan, S.; Ahmad, I. Synthesis, Structural Elucidation, and Antibacterial Evaluation of Some New Molecules Derived from Coumarin, 1,3,4-Oxadiazole, and Acetamide. *Org Chem Int* **2016**, *2016*, 1–10.
